# Magic Mushrooms? White-Rot Fungal Degradation of Psychoactive Pharmaceuticals in Biosolids

**DOI:** 10.1021/acsenvironau.5c00258

**Published:** 2026-01-29

**Authors:** Kate Burgener, Carsten Prasse

**Affiliations:** † Department of Environmental Health & Engineering, 1466 Johns Hopkins University, Baltimore, Maryland 21218, United States; ‡ Risk Sciences and Public Policy Institute, Johns Hopkins Bloomberg School of Public Health, Baltimore, Maryland 21205, United States

**Keywords:** biodegradation, bioremediation, mycoremediation, antidepressants, sewage sludge, LC-HRMS, detoxification

## Abstract

Biosolids, the solid byproducts of wastewater treatment, are widely applied to soils to enhance nutrient levels and organic matter. However, their use raises environmental and human health concerns due to the presence of anthropogenic organic contaminants. As such, there is a need to develop treatment strategies that can help remove these compounds before biosolids are land applied. This study investigates the potential of two white-rot fungal species to remove nine psychoactive pharmaceuticals from biosolids. Each species degraded eight compounds, achieving removal efficiencies between 48 and 99% after 60 days. *Pleurotus ostreatus* nearly completely (>90%) degraded desvenlafaxine, trazodone, and citalopram, while *Trametes versicolor* achieved over 75% degradation of desvenlafaxine, trazodone, and lamotrigine. Liquid culture (without biosolids) and biosolid experiments tentatively identified 41 fungal transformation products (27 for *P. ostreatus* and 36 for *T. versicolor*), of which many were formed from cleavage, hydroxylation, or demethylation reactions. These findings demonstrate that white-rot fungi can effectively grow on biosolids and degrade sorbed psychoactive pharmaceuticals. Overall, the results highlight mycoaugmentation as a promising and sustainable approach for mitigating pharmaceutical contamination in biosolids prior to land application.

## Graphical abstract

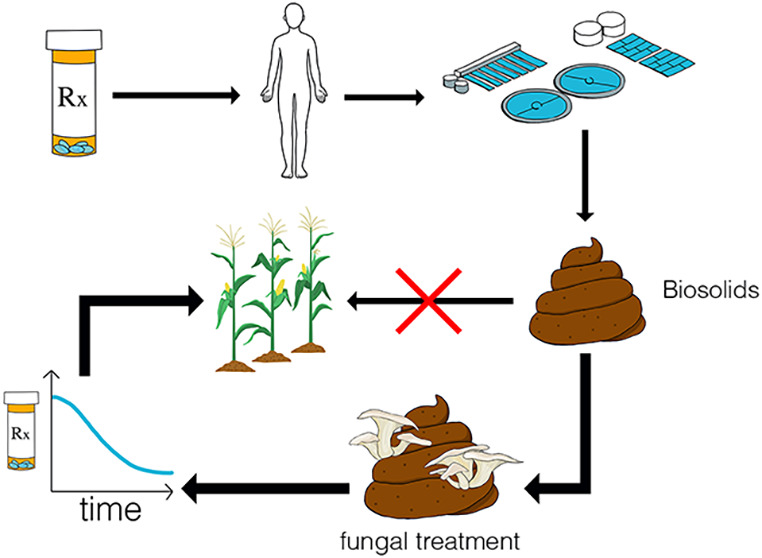

## Introduction

Biosolids are the stabilized product of sewage sludge digestion in water resource recovery facilities. They are rich in organic carbon, nitrogen, and phosphorus, which makes biosolids a good source of nutrients and organic matter to promote plant growth. Roughly 60% of the 4 million dry-metric tons of biosolids produced in the United States during 2023 were applied to lands as agricultural fertilizer, landscaping substrate, and reclamation backfill.^1^ Due to their contact with crops grown for human consumption as well as animal feed, land applied biosolids are subject to the U.S. Clean Water Act.^2^ An amendment to the law in 1987 required the EPA to assess environmental risks associated with sewage sludge management; however, standards for detectable pathogen levels and maximum allowable metal concentrations were not published until 1995. Though not currently regulated, increasing evidence for the presence of anthropogenic organic chemicals in biosolids has led to more scrutiny of the fate of these biosolid-associated organic compounds (BOCs) when biosolids are introduced into the environment. Commonly identified BOCs with known environmental hazards include preservatives (e.g., triclocarban), pharmaceuticals (carbamazepine), biocides (fludioxonil), and surfactants (PFAS).^3^ Research conducted on BOC fate and transport in the environment indicates that leaching into groundwater and uptake into edible plants can occur, highlighting the potential risks for human exposure.^4–11^

Existing approaches for biosolid treatment have shown limited success in removing recalcitrant organic contaminants. Treatment strategies using anaerobic or aerobic microorganisms have been evaluated for BOC degradation; however, anaerobic research generally focuses on liquid cultures with nitrate- or sulfate-reducing conditions and assumes that the degradation mechanisms and pathways exhibited are similar in real-world applications (i.e., in the presence of (bio)­solids).^12–14^ Partial removal of pesticides and pharmaceuticals in aerobic biosolid slurry was demonstrated with biostimulation of native microbes (by adding additional carbon sources) or the inclusion of enzymatic pretreatments.^15^ The effectiveness of biostimulation is defined by the microbial consortia’s compatibility with the stimulating agent, and bacterial degradation requires BOCs to be bioavailable, which is limited if they are strongly sorbed onto biosolid surfaces.^15,16^ Similarly, enzymatic treatments (e.g., using protease, cellulase, or laccase) require at least partial availability of the contaminant and can be limited by suboptimal matrix pH, though research has shown that some organic contaminants do degrade when enzymatic pretreatments are applied to sludge.^15,17,18^ Additional approaches to mitigating organic contaminant load in biosolids involve thermal degradation and ozonation, but high energy requirements limit the feasibility of these processes.^19,20^ Given the heterogenicity of the biosolid matrix and the large amount produced annually, biosolid treatments need to be inexpensive, flexible, and robust.

Over the last 30 years, research examining white-rot fungi has shown that the fungi can degrade a large spectrum of organic compounds even in highly contaminated matrices.^21–23^ White-rot fungi are a class of xylophagous fungi that break down cellulose and lignin in plants; these biomolecules are very stable, complex, and heterogeneous. The fungi degrade these molecules by excreting extracellular enzymes which are nonspecific and thus have been increasingly investigated for different remediation efforts (e.g., paper and pulp waste, contaminated soils, wastewater).^22^ As the mycelial networks grow throughout the matrix, fungal cells release these exoenzymes to break down organic matter that would otherwise not be readily bioavailable, which is particularly promising for the treatment of biosolids where BOCs can be strongly sorbed to organic matter.

Similar to microbial degradation studies, previous research looking at white-rot fungal degradation of BOCs generally only investigated degradation in liquid culture.^24–27^ While these studies provide insights into whether a specific organic compound can be degraded by white-rot fungal enzymes, the results cannot necessarily be extrapolated to the removal under real-world conditions, such as compounds sorbed to biosolids. Contaminant bioavailability, fungal growth, and enzyme production by fungal secondary metabolism are all factors that affect the capability of white-rot fungal degradation.^28^ These considerations make fungal degradation of organic compounds on solids difficult to predict because fungal growth and secondary metabolism are different in liquid culture. However, a limited number of studies have demonstrated that degradation of organic contaminants by white-rot fungi when grown on substrate like cotton stalks, as well as on contaminated matrices like raw sewage sludge and soils, is possible.^29–31^

In the present study, we aim to determine whether white-rot fungi are a treatment strategy for degrading BOCs *in situ* on biosolids. Two white-rot fungi, *Pleurotus ostreatus* and *Trametes versicolor*, were used since both fungi are well characterized and have demonstrated broad degradation potential.^22^ Nine psychoactive chemicals were chosen as model compounds based on their recalcitrance and widespread presence in biosolids (Tables 1 and S1).^32,33^ Three of the compounds (amitriptyline, vilazodone, and trazodone) have never been assessed for white-rot fungal biodegradation, and compounds previously studied in liquid culture (carbamazepine, citalopram, sertraline, desvenlafaxine, fluoxetine, and lamotrigine) were also included to compare our results with previously published data (Table S3). To study the removal of these compounds in biosolids, they were added to the biosolids and then incubated with white-rot fungi for up to 60 days. Compound concentrations were measured over time, and removal efficiencies were calculated. In addition, the compounds were exposed to the white-rot fungi in liquid growth media for 20 days, and concentrations in media were measured over time. Experiments in liquid culture (i.e., in the absence of biosolids) were used to identify fungal metabolites with both nontargeted and semitargeted liquid chromatography-high-resolution mass spectrometry (LC-HRMS) workflows and were used to compare metabolites in both the presence and absence of biosolids.

**Table 1:**
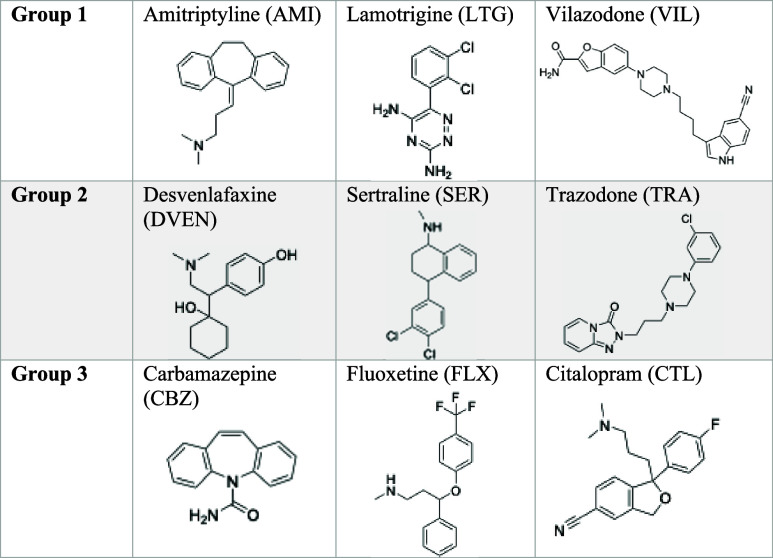
Psychoactive Pharmaceuticals Studied for White-Rot Fungal Degradation Kinetics^a^

a Nine compounds were chosen due to their increased usage around the world, their detection in biosolids, and their persistence in the environment.

## Materials and Methods

### Chemicals

Amitriptyline HCl (AMI; 98%) and Fluoxetine HCL (FLX; 98%) were purchased from Tokyo Chemical Industry (Portland, OR, USA). Carbamazepine (CBZ, 98%) was purchased from Toronto Research Chemicals (Toronto, Ontario, Canada). Trazodone HCl (TRA, 99%), Citalopram HBr (CTL, 98%), and Vilazodone HCl (VIL, 98%) were purchased from MilliporeSigma (St. Louis, MO, USA). Lamotrigine (LTG; 98%), Desvenlafaxine (DVEN; 97%), and Sertraline HCl (SER; 97.5%) were purchased from Thermo Scientific (Waltham, MA, USA). Internal standards Desvenlafazine-d6 succinate H_2_O, Sertraline-d3 HCl, Carbamazepine-d10, Fluoxetine-d5 HCl, Lamotrigine-d3, and Amitriptyline-d6 HCl were purchased from CDN Isotopes (Pointe-Claire, QC, Canada), and Vilazodone-d8 was purchased from MedChem express (Monmouth Junction, NJ, USA).

Stock solutions of psychoactive compounds were prepared in methanol at a concentration of 0.1 mg/mL. Performing individual degradation experiments of nine different compounds by two species of white-rot fungi in two different matrices would be very costly and time-consuming; therefore, the compounds were combined into three groups (Table 1) which were spiked into matrices together. To facilitate the identification of metabolites, compounds with similar moieties were assigned different groups. Functional groups and moieties which were strategically separated are dichlorobenzene (lamotrigine and sertraline), piperazine (vilazodone and trazodone), cyano (vilazodone and citalopram), and tertiary amine (amitriptyline and desvenlafaxine). In Group 3, both fluoxetine and citalopram have at least one fluorine; the strength of C–F bonds suggests that defluorination by enzymatic oxidation is highly unlikely and therefore two compounds with fluorine would likely not impede metabolite identification.

### Fungal Strains, Culture Conditions, and Cell Suspensions

The strain of *P. ostreatus* was Florida F6 (ATCC#58053), and the strain of *T. versicolor* was PAP 52 (ATCC #20869). *P. ostreatus* was grown on glucose peptone media (GPM), which contained 20 g/L glucose, 5 g/L peptone, 2 g/L yeast extract, 1 g/L K_2_HPO_4_, and 0.5 g/L MgSO_4_·7H_2_O (pH 7).^27^ *T. versicolor* was grown on malt extract media (MEM), which contained 20 g/L malt extract (pH 4.5).^34^ Solid cultures were maintained on Petri dishes containing GPM or MEM solidified with agar (1.5 or 2%, respectively). Mycelial cell suspensions of each fungus were prepared by a procedure adapted from Marco-Urrea et al.^34^ Nine 0.5 cm^2^ agar plugs from actively growing culture were added to 150 mL growth media in Erlenmeyer flasks, which were incubated in the dark at 25 °C on an orbital shaker (125 rpm, *r* = 1.9 cm). Following 7 days of growth, the mycelia was homogenized with a tissue homogenizer (Omni International, GA) to obtain a homogeneous cell suspension.

### Biosolid Solid-State Fermentation Experiments

Solid-state experimental procedures were adapted from Rodriguez-Rodriguez et al.^29^ Eight grams of autoclaved biosolids were spiked with a mixture of three compounds (Table 1) which contained 1000 ng/g (dry weight) of each psychoactive compound and were left sitting for an hour to allow compounds to sorb onto biosolids. Biosolids were then transferred to 75 cm^2^ Nunc EasYFlasks with filter caps (Thermo Fisher Scientific, WI). Fifteen grams of previously hydrated (1:2 w/v) and thrice-autoclaved wheat straw were added to each flask, followed by the inoculation of blended mycelial suspension (described above) at 0.25 mL/g solid dry weight. Wheat straw was used as bulking material to provide structural support for the mycelia and increase the carbon/nitrogen ratio of the matrix to encourage secondary metabolism.^35^ Experimental flasks were briefly shaken to make a homogeneous blend and stored in the dark at 25 °C. Samples were kept hydrated with Milli-Q water over the experimental period (approximately 3 mL every 3 days). Samples were sacrificially sampled in triplicate at eight time points (0, 7, 14, 21, 30, 40, 50, and 60 days). Experimental controls included biosolids and wheat straw not inoculated with fungi. For each sampling, the entire contents of the flask was transferred to a 50 mL Falcon tube, lyophilized, and stored at −80 °C until extraction.

### Liquid Degradation Experiments

The homogeneous cell suspension (1 mL) was added to 10 mL of fresh growth media in individual 50 mL Erlenmeyer flasks that were grown for 7 days at the same conditions described above. After 7 days of incubation, a single mycelial mass formed which was added to 10 mL of fresh growth media in 25 cm^2^ Nunc EasYFlask untreated polystyrene culture flasks with filter caps (Thermo Fisher Scientific, WI). A mixture of three compounds, which contained 1000 ng/mL of each psychoactive compound in the group, was added to each experimental flask and stored in the dark at 25 °C. Samples were sacrificially collected at six time points (0, 2, 5, 9, 14, and 20 days) in triplicate. For each sampling, media was sampled; the mass of the fungal culture was measured in dry weight, and the pH of the growth media was recorded. Experimental controls included heat-killed culture and uninoculated samples. Heat-killed culture samples were autoclaved prior to being introduced into the culture flasks and were used to determine whether compound sorption onto the surface of the fungal mass is occurring over time.

### Extraction and Instrumental Analysis

Solid degradation samples were extracted with a methanol solid–liquid extraction procedure and analyzed with LC-HRMS (details in Text S1). For liquid degradation experiments, growth media was diluted and filtered before analysis with LC-HRMS (Text S1). Extracts were analyzed by liquid chromatography with high-resolution Orbitrap mass spectrometry. Details on methodological settings and parameters as well as quantification are detailed in the SI instrumental analysis text and Tables S4 and S5. Vilazodone could not be quantified because the addition of wheat straw to the matrix inhibited extraction in all three extraction methods tested. However, vilazodone was able to be quantified in the liquid fungal cultures and is discussed below in the liquid culture results.

### Data Analysis

Results for liquid and solid-state fermentation were reported as removal efficiency (*R*_e_) relative to abiotic controls

1 $$R_{e}=\left[1-\frac{C\left(t\right)}{C_{c}\left(t\right)}\right]\times 100$$

where the percent removed from the matrix is a function of the compound concentration at the end time point (*C*(*t*)) and the concentration of the compound in the abiotic controls (liquid growth media or biosolids/wheat straw without any fungi, *C*_c_(*t*)). For biosolid experiments, the removal efficiency reported can be wholly attributed to biodegradation by white-rot fungi in the system. In liquid culture, removal efficiency cannot differentiate between degradation, adsorption of, or absorption into the fungal mass because only the concentration in the media was measured over time. The identification of fungal metabolites is an indicator of white-rot fungal degradation (as opposed to sorption removal). The statistical differences between fungal degradation were determined using a two-tailed *t* test (*p* < 0.05) with MATLAB and Statistics Toolbox release 2024b (The Math Works Inc., Natick, Massachusetts).

Raw LC-HRMS data were processed with Thermo Scientific software Compound Discoverer v3.3. Full workflow information is given in Table S6. The output of Compound Discoverer was further filtered with MATLAB using a Peak Rating filter cutoff of 6.5 and a replicate threshold of 2/3. Metabolites were tentatively identified via nontargeted analysis in Compound Discoverer by identifying features that increased over time, comparing features that had MS2 fragments that matched base structure fragments of the parent compounds, and comparing fragmentation spectra to the Thermo’s MzCloud database. Likely metabolite structures were determined using Compound Discoverer’s fragment ion searching (FISh) which predicts *in silico* MS2 fragment structures based on proposed molecular structures. In addition, mass lists were created to facilitate the identification of compounds previously identified in the literature as degradation products and predicted oxidation transformations of the parent compounds. To this end, phase 1 (CYP450) transformation and environmental microbial transformation metabolism predictions were generated using BioTransformer 3.0 (https://biotransformer.ca/). Metabolites were examined and assigned confidence levels based on the Schymanski criteria.^36^

In addition to assigning confidence to the chemical structure, the identified metabolites were also assessed for toxicity using EPA’s cheminformatics Hazard Comparison Module (HCM, https://www.epa.gov/comptox-tools/cheminformatics) to determine if they might be more hazardous than their parent compounds.^37^ The module generates a hazard profile by combining publicly available toxicological data and QSAR-based predictions for multiple toxicity end points. Hazard data are reported by letter score of very high (VH), high (H), medium (M), low (L), and inconclusive (I) for each toxicity end point. A total of ten human and ecological end points were considered for this analysis: “acute mammalian oral toxicity”, “carcinogenicity”, “genotoxicity/mutagenicity”, “endocrine disruption”, “reproductive toxicity”, “developmental toxicity”, “acute aquatic toxicity”, “chronic aquatic toxicity”, “environmental persistence”, and “bioaccumulation”. To compare metabolites, an average hazard score was calculated using

2 $$\mathrm{average}\;\mathrm{hazard}\;\mathrm{score}=\frac{\Sigma \;\mathrm{individual}\;\mathrm{hazard}\;\mathrm{scores}}{\mathrm{number}\;\mathrm{of}\;\mathrm{end}\;\mathrm{points}\;\mathrm{with}\;\mathrm{data}}$$

where letter scores were converted to numerical values 1 to 4 corresponding with low to very high.^3^ Inconclusive and missing scores were not counted. An average quality score (eq 3) for each of the metabolites was calculated by converting the hazard data source authorities into numerical scores 1, 2, and 3 for QSAR-based model predictions, screening data, and authoritative data, respectively, and averaging across the available end points

3 $$\mathrm{average}\;\mathrm{quality}\;\mathrm{score}=\frac{\Sigma \;\mathrm{individual}\;\mathrm{quality}\;\mathrm{scores}}{\mathrm{number}\;\mathrm{of}\;\mathrm{end}\;\mathrm{points}\;\mathrm{with}\;\mathrm{data}}$$

where a higher average quality score indicates a greater confidence in the data used to determine the average hazard score. A compound with an authoritative toxicity source will have a higher average quality score than a compound with a toxicity end point determined by QSAR modeling.

To account for the quality of the toxicity data in comparison of hazard scores, we calculated a quality-adjusted hazard score (eq 4) for each metabolite.

4 quality−adjustedhazardscore=averagehazardscore×averagequalityscore

This score encompasses both the degree of predicted toxicity and the source of the predicted toxicity. In addition, a completeness score (eq 5) was also calculated

5 $$\mathrm{completeness}\;\mathrm{score}=\frac{\mathrm{number}\;\mathrm{of}\;\mathrm{end}\;\mathrm{points}\;\mathrm{with}\;\mathrm{data}}{\mathrm{number}\;\mathrm{of}\;\mathrm{end}\;\mathrm{points}\;\mathrm{searched}}$$

where a higher completeness score indicates fewer missing data for the toxicity end points considered. A compound with many missing or inconclusive values may have a high average hazard score due to a low number of end points with data; however, if many end points are not accounted for, it becomes difficult to truly compare toxicity; therefore, the completeness score is used to qualitatively assess the impact of the quality-adjusted average hazard scores.

## Results and Discussion

### Removal of Pharmaceuticals by White-Rot Fungi Grown on Biosolids

Of the nine psychoactive compounds spiked onto the biosolids, eight were detected and quantified and are discussed further in more detail. Despite detecting vilazodone in liquid (as discussed below), we were unable to detect it in experiments with biosolids, possibly due to matrix interferences. Of the psychoactive compounds quantified, all were substantially removed from the biosolids over a 60-day period by both fungal species (Figure 1). Removal efficiencies, as opposed to relative removal, account for the compound’s behavior in abiotic controls over time, which is why they were used to report results. The average removal efficiencies (*R*_e_) were similar (within 5%) among *P. ostreatus* and *T. versicolor* for desvenlafaxine (*R*_e_ = 99 and 96%), lamotrigine (*R*_e_ = 79 and 80%), and fluoxetine (*R*_e_ = 48 and 51%). *P. ostreatus* had greater removal efficiencies than *T. versicolor* for trazodone (*R*_e_ = 94 and 82%), citalopram (*R*_e_ = 93 and 77%), amitriptyline (*R*_e_ = 76 and 63%), sertraline (*R*_e_ = 74 and 65%), and carbamazepine (*R*_e_ = 73 and 64%).

**Figure 1:**
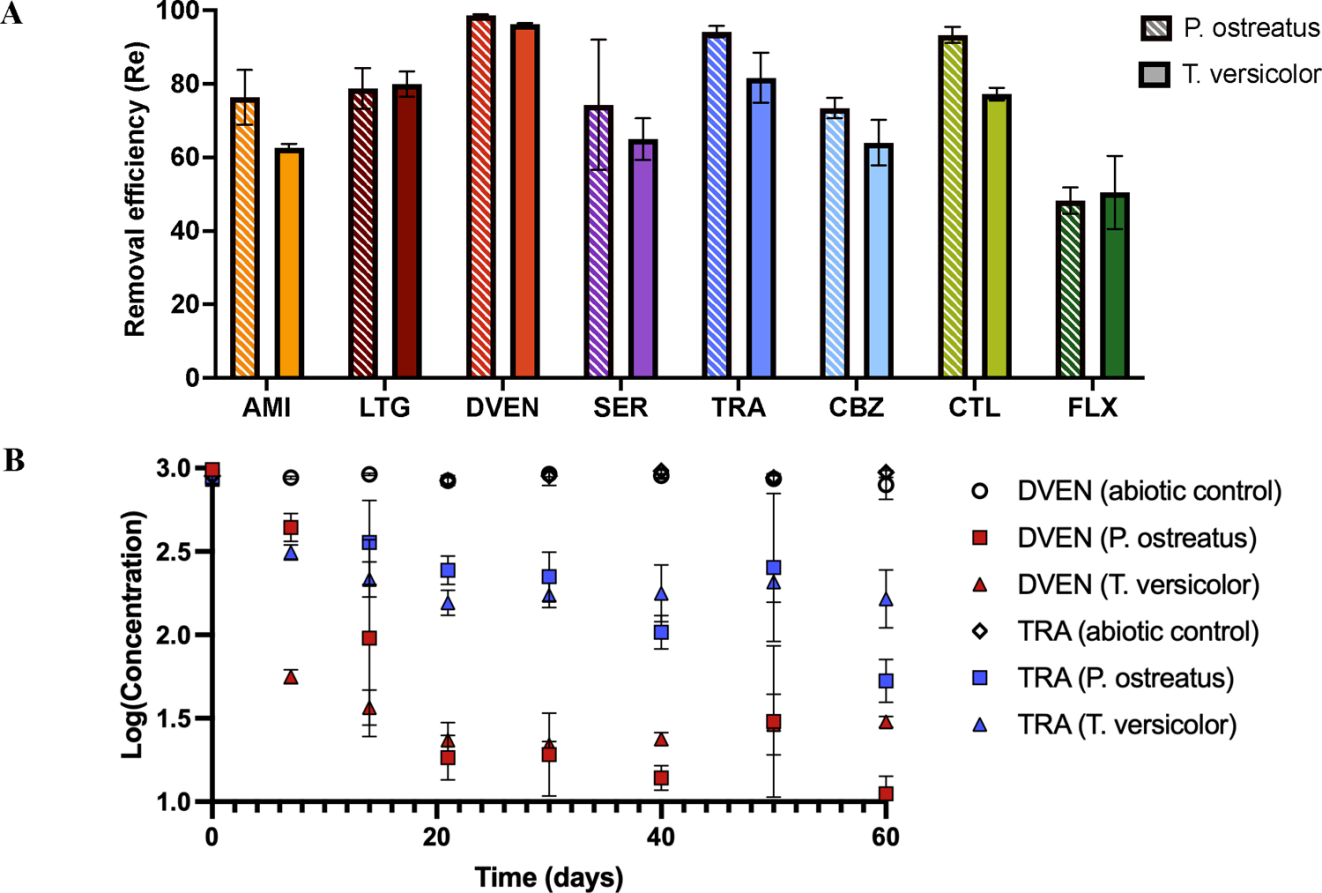
Removal efficiency of psychoactive pharmaceuticals sorbed to biosolids in the presence of white-rot fungi. Degradation of amitriptyline (AMI), lamotrigine (LTG), desvenlafaxine (DVEN), sertraline (SER), trazodone (TRA), carbamazepine (CBZ), citalopram (CTL), and fluoxetine (FLX) were quantified over a period of 60 days. (A) Overall removal efficiencies for *P. ostreatus* and *T. versicolor* show greater than 45% degradation for all compounds. (B) Over 95% of desvenlafaxine and 75% of trazodone were degraded by *P. ostreatus* and *T. versicolor* within 21 days of the 60-day experiment. Abiotic controls were biosolids without fungi. The removal efficiency values are means and error bars are standard deviations.

The calculated removal efficiency is reported as percent removal, which considers the behavior of the compounds in abiotic controls. Because the control experiments allow us to account for any abiotic degradation occurring in the absence of fungi, the reported degradation of the pharmaceuticals can be attributed to white-rot fungi. Additionally, the presence of psychoactive transformation products in fungal samples provides confidence that white-rot fungal enzymes are primarily responsible for the degradation of these compounds (see discussion below). Time-point concentrations for each individual compound are shown in Figure S1. The removal efficiencies of lamotrigine, sertraline, carbamazepine, and fluoxetine were not statistically significant (*p* = 0.05) between white-rot fungal species, though *P. ostreatus* had a slightly greater average percent degradation than *T. versicolor* for all compounds after 60 days except for fluoxetine. Initially, compounds exposed to *T. versicolor* degraded faster, but by day 30 degradation plateaued at similar concentrations for both species. *P. ostreatus* had statistically greater removal efficiencies for amitriptyline, desvenlafaxine, trazodone, and citalopram. Overall, these results suggest that both *P. ostreatus* and *T. versicolor* can transform psychoactive compounds present in biosolids, though *P. ostreatus* may be better suited for mycoaugmentation of biosolids based on its greater removal of the pharmaceuticals and its high tolerance for different growth conditions.

None of the compounds have been previously studied for degradation by white-rot fungi in biosolids, but carbamazepine has been investigated for degradation on other solid substrates, including cotton stalks and raw sewage sludge.^30^ Golan-Rozen et al. found that 82% of carbamazepine was transformed by *P. ostreatus* over a 60-day period of growth on cotton stalks. For biosolids, we observed that the average percent degradation of carbamazepine was 73% by *P. ostreatus*. Rodriguez-Rodriguez et al. grew *T. versicolor* on a mixture of sewage sludge and wheat straw; after the culture had been established, they spiked carbamazepine into the system and quantified degradation over 4 days.^29^ They found that approximately 48% of the carbamazepine degraded, which is comparative to the percent degradation we observed at 14 days (47%) (Figure S1); additionally, the average degradation found in our study by *T. versicolor* over the duration of 60 days was 64% which is greater than previously reported in liquid or solid media. It is important to consider that the *T. versicolor* culture used by Rodriguez-Rodriguez et al. had already matured before carbamazepine was added to the system. To more realistically determine whether compounds sorbed to biosolids are bioavailable to white-rot fungi, our sampling started at the same time the fungi were inoculated into the biosolid matrix.

### Comparison of Degradation between Biosolids and Liquid Culture

Liquid culture degradation experiments used to facilitate the identification of fungal metabolites also allowed for a comparison of the degradation behavior in the presence and absence of biosolids. The removal efficiency of the nine psychoactive pharmaceuticals by *P. ostreatus* in liquid growth media at 20 days ranged from 20 to 100%. The compounds with the highest average removal were trazodone (*R*_e_ = 100%), lamotrigine (*R*_e_ = 94%), desvenlafaxine (*R*_e_ = 94%), and vilazodone (*R*_e_ = 93%). *P. ostreatus* partially removed sertraline (*R*_e_ = 83%), fluoxetine (*R*_e_ = 58%), amitriptyline (*R*_e_ = 53%), citalopram (*R*_e_ = 37%), and carbamazepine (*R*_e_ = 20%). This is the first time amitriptyline, trazodone, and vilazodone have been studied for removal by white-rot fungi, so there are no previous removal efficiencies to compare results to. However, carbamazepine (48–99%), desvenlafaxine (100%), lamotrigine (96%), sertraline (46–94%), citalopram (0–50%), and fluoxetine (0–20%) have all been demonstrated to have been at least partially degraded by *P. ostreatus* in liquid media.^25,26,38,39^ The observed degradation of desvenlafaxine lamotrigine, sertraline, and citalopram by *P. ostreatus* is similar to previous studies.^25,26,38,39^ The removal efficiency of carbamazepine was far lower than previous liquid experiments, and the removal of fluoxetine was greater than any removal previously reported.^26,27,30,38^ The high variance in results indicates that for degradation in liquid media, experimental conditions such as initial concentration, pH, growth media, time, and even white-rot fungal species strains can all impact removal efficiency.

For *T. versicolor*, the removal efficiencies of the pharmaceuticals in liquid media after 20 days ranged from 28.4 to 99.6% (Figure 2). Highest removals were observed for trazodone (*R*_e_ = 100%), desvenlafaxine (*R*_e_ = 98%), and vilazodone (*R*_e_ = 97%). Over half of sertraline (*R*_e_ = 83%) and fluoxetine (*R*_e_ = 52%) were removed; *T. versicolor* partially degraded (≤50%) amitriptyline (*R*_e_ = 50%), carbamazepine (*R*_e_ = 48%), citalopram (*R*_e_ = 40%), and lamotrigine (*R*_e_ = 28%) from the liquid media system. Degradation of both carbamazepine and desvenlafaxine was comparable to previous research using *T. versicolor*.^39,40^ All other compounds have never been investigated for degradation by this white-rot fungal species.

**Figure 2:**
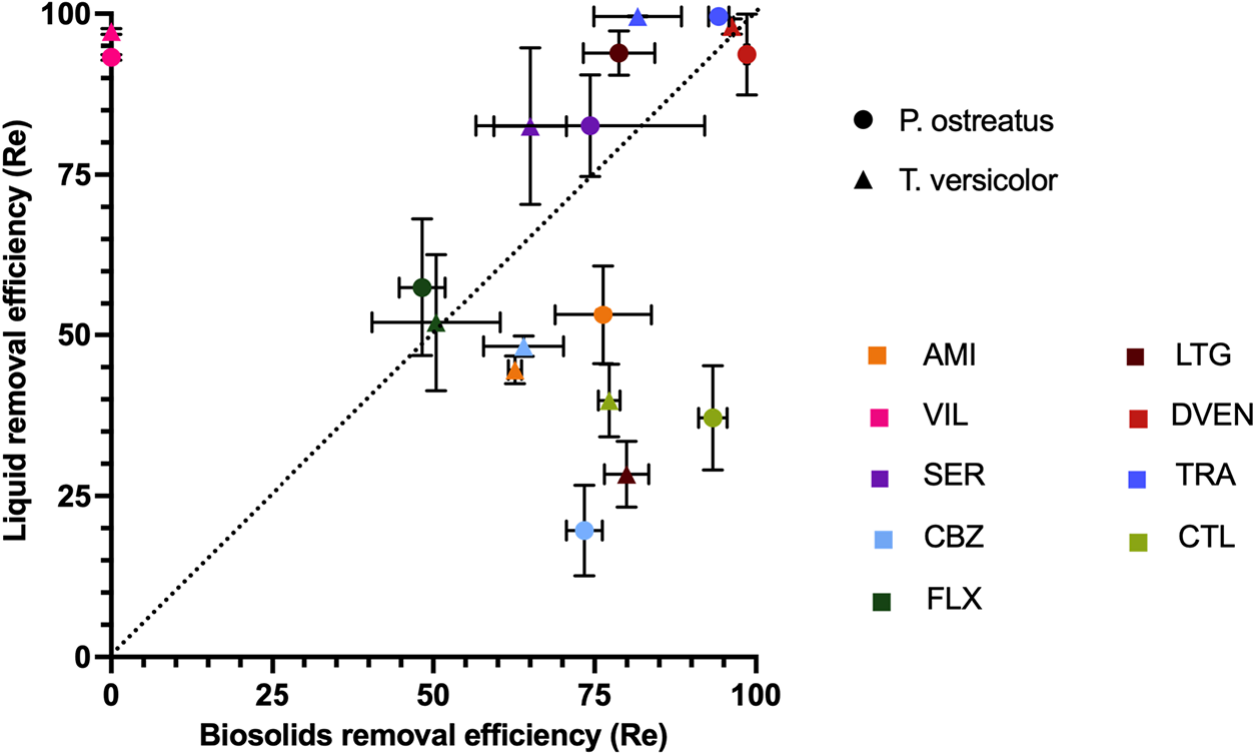
Psychoactive pharmaceutical removal in liquid culture and on biosolids when exposed to white-rot fungi. The final removal efficiencies for liquid and solid culture were compared for all psychoactive pharmaceutical compounds exposed to *P. ostreatus* or *T. versicolor*. Amitriptyline (AMI), lamotrigine (LTG), desvenlafaxine (DVEN), sertraline (SER), trazodone (TRA), carbamazepine (CBZ), citalopram (CTL), and fluoxetine (FLX) were all quantified in both matrices while vilazodone (VIL) was not detected in biosolid samples. Amitriptyline, carbamazepine, and citalopram were degraded more on biosolids than in liquid by both species of fungi. Differences in percent removal between liquid and biosolids demonstrate the importance of performing degradation experiments with relevant matrices. The dashed line is a slope of 1. Data values are means and error bars are standard deviations of the replicate samples.

Comparing the percent removal of each compound between liquid growth media and biosolids shows significant differences for most compounds (Figures 2 and S8). Amitriptyline, carbamazepine, and citalopram exhibited greater removal in biosolids than in liquid culture samples by both *P. ostreatus* and *T. versicolor*. Average removal efficiencies of sertraline and trazodone were greater in liquid growth media than biosolids for both fungi. Lamotrigine exposed to *P. ostreatus* was removed 15% more in liquid samples, whereas in *T. versicolor* samples, 52% more lamotrigine was removed in biosolids than in liquid samples. Both desvenlafaxine and fluoxetine had similar removal efficiencies between liquid and biosolids for both white-rot fungal species. Vilazodone could not be quantified in the biosolids, so no direct comparisons can be made between matrices. However, the high removal of vilazodone in liquid culture indicates that it is generally degradable by white-rot fungi.

Data from this study indicate that liquid media experiments may not be the best indicator of how biodegradable a compound is in solid matrices, given how different removal efficiencies were between matrices. We originally hypothesized that compound degradation would be lower when spiked onto biosolids because of reduced bioavailability toward fungal enzymes when adsorbed to biosolids/straw. However, no clear trends were observed between physicochemical properties such as p*K*_a_, log *K*_OC_, log *K*_OW_ (Table S2), and removal efficiency to explain why psychoactive compounds were degraded in different amounts between solid and liquid matrices when exposed to the same fungal culture. For sertraline, which has the highest log *K*_OC_ and log *K*_OW_ of the investigated compounds, an increased degradation was observed in liquid vs solid samples (increase of 8 and 18% in experiments with *P. ostreatus* and *T. versicolor*, respectively). In contrast, citalopram (which has the second largest log *K*_OC_) and amitriptyline (which has the second largest log *K*_OW_) both degraded more in biosolids vs liquid samples (increase of 56 and 37% for citalopram and increase of 23 and 18% for amitriptyline in experiments with *P. ostreatus* and *T. versicolor*, respectively). These results indicate that sorption to biosolids is not the only factor influencing degradation.

Differences in the fungal growth behavior of mycelia grown in liquid and solid media affect enzyme distribution within the system. White-rot fungi grown in stationary liquid media form a mass at the media–air interface; the degradation of compounds spiked into the media relies on either the diffusion of extracellular enzymes into the matrix or the diffusion of the compound into the fungal mass for intracellular enzymatic degradation.^41^ In solid culture, white-rot fungal mycelia spread throughout the matrix, excreting enzymes to break down the bulk material (wheat, cotton, straw, and others), so spiked compounds would need to be adjacent to mycelia to be degraded. The production of extracellular enzymes depends on the solid substrate the fungi are grown on which vary in lignin, cellulose, and hemicellulose composition.^35^ Additionally, the state of the bulk substrate can affect the amount of enzyme produced, for example, the use of ground straw versus cut straw has been demonstrated to induce significant differences in enzyme production.^42^ For compounds such as carbamazepine and citalopram, which had greatly differing percent removals between biosolids and liquid media, it is possible that the enzymes that metabolize the parent compound were produced by the fungi in higher quantities in biosolids over the liquid matrix. Indeed, in the liquid growth media, all of the sugar and proteins that the fungi require to grow are readily accessible, whereas in the biosolids, the fungi need to break down the substrate for carbon and nitrogen, encouraging enzyme production.^40^ Future work should thus focus on measuring specific enzymatic activities in both matrices over time to see how nutrient availability affects white-rot fungal enzyme production and subsequent compound degradation.

### Elucidation of Fungal Metabolites

We used nontargeted analysis combined with computational metabolite prediction tools to tentatively identify metabolites in liquid media and biosolids to determine whether fungal degradation mechanisms were consistent between both matrices for the nine psychoactive pharmaceuticals. A total of 41 metabolites were identified among the samples (Tables 2 and S7–S9). Compounds with the most metabolites were desvenlafaxine (10), amitriptyline (8), trazodone (8), and carbamazepine (8); three or fewer metabolites each for citalopram, sertraline, vilazodone, and fluoxetine were identified, and none were identified for lamotrigine. Overall, 27 metabolites were identified in experiments with *P. ostreatus* and 36 were found for *T. versicolor*. Proposed degradation pathways and graphs of the products over time in both matrices can be found in the Supporting Information (Figures S9–S16 and Tables S10–S16). Over half of the metabolites overlapped between the two white-rot fungi, indicating similarities in the biodegradation mechanisms of *P. ostreatus* and *T. versicolor*. As expected, the most prevalent transformations include hydroxylation, cleavage, demethylation, and *N*-oxide formation. These are all known transformation reactions of extracellular enzymes that white-rot fungi produce.^22^ Of the 41 metabolites, 35 were found in liquid media, while 15 were found in biosolid experiments.

**Table 2:**
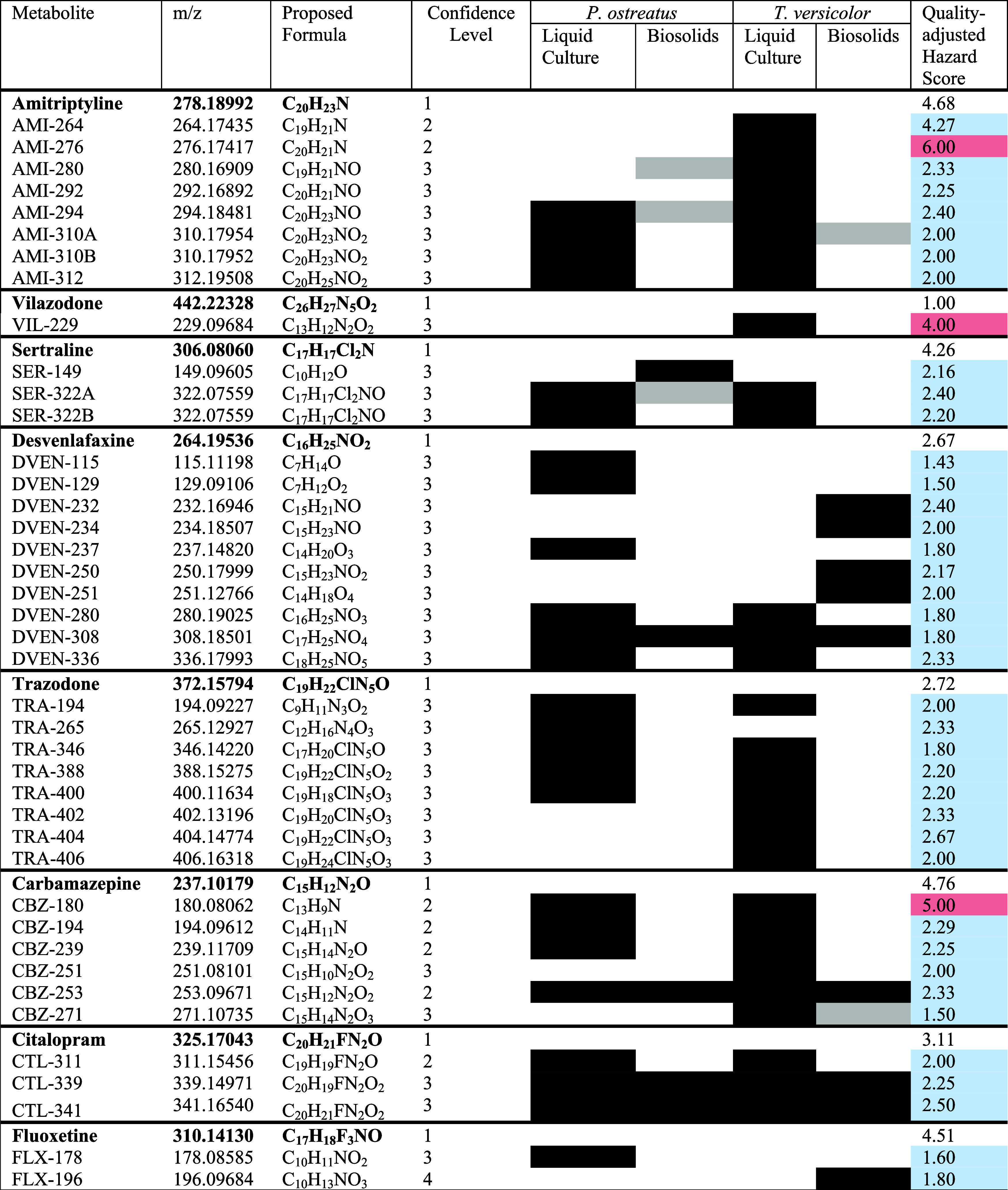
Metabolites of Psychoactive Pharmaceuticals in Liquid and Biosolids by White-Rot Fungi *P. ostreatus* and *T. versicolor* ^a^

a Measured m/z, chemical formula, nontargeted confidence level, presence in different samples, and quality-adjusted hazard scores are given for each metabolite. Compound presence filled with black indicate MS2 data while gray filling indicates that there were no MS2 data measured for the metabolite in that matrix. Quality-adjusted hazard scores filled with blue had lower scores than their parent compound while scores filled with red had greater values than parent compounds.

#### Desvenlafaxine

Among the ten desvenlafaxine metabolites, three were found exclusively in *P. ostreatus* samples, four were found only in *T. versicolor* samples, and three were found in both fungal species samples. Five metabolites were only found in liquid culture samples, four were found exclusively in biosolids, and one was found in samples from both matrices. The tentatively identified structures of all of the metabolites and the proposed transformation pathways are shown in Figure 3. The results indicate that initial fungal metabolic reactions involved demethylation, oxidations (hydroxylation and carboxylation), and C–C cleavage, which are known white-rot fungal transformations.^41^ Demethylation of the tertiary amine group was limited to products from *T. versicolor* grown on biosolids; however, this product has been reported previously by both *P. ostreatus* and *T. versicolor* in liquid biodegradation experiments.^39^ Metabolites with aliphatic C–C cleavage appeared only in *P. ostreatus* liquid samples. DVEN-308 was the only product found in both matrices and transformed by both species of fungi; carboxylation reactions of aromatic groups in lignin have been reported by white-rot fungi grown on wheat straw.^43^ DVEN-250 is the only white-rot fungal metabolite of desvenlafaxine previously identified, though DVEN-250 and DVEN-280 have been identified as metabolites of desvenlafaxine in humans.^39,44^

**Figure 3:**
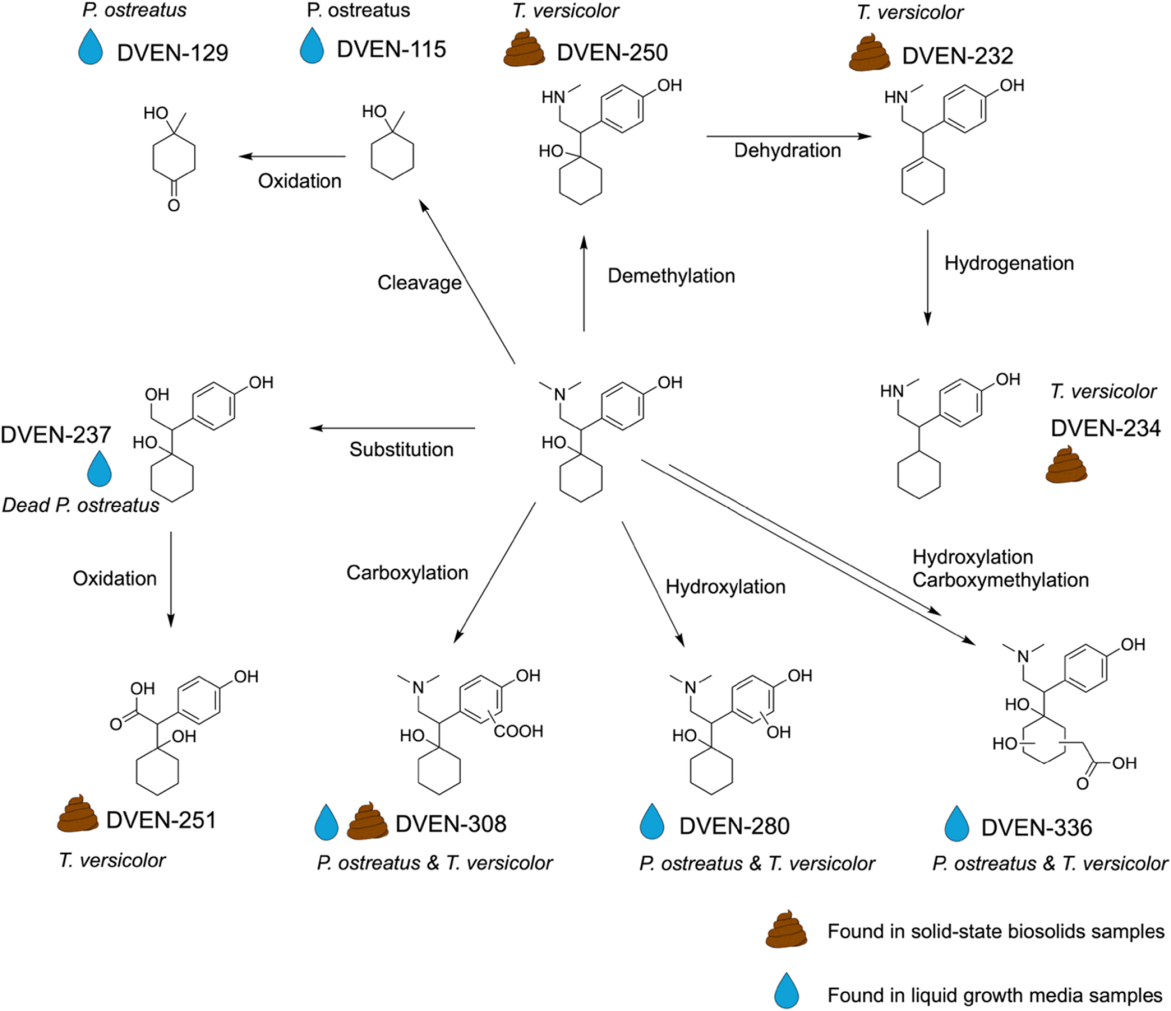
Proposed transformation pathway of desvenlafaxine (DVEN) by white-rot fungi, *P. ostreatus* and/or *T. versicolor*. Tentative metabolites of DVEN in liquid and biosolid degradation experiments were identified. DVEN-250 has been previously reported as a transformation product of DVEN by white-rot fungi. Metabolites DVEN-280, DVEN-250, and DVEN-251 were predicted products by BioTransformer 3.0. Compounds DVEN-232, DVEN-234, DVEN-308, and DVEN-336 have not been reported previously.

#### Amitriptyline

Eight metabolites were identified from amitriptyline degradation, all of which were found in *T. versicolor* liquid samples, and five of which were found in *P. ostreatus* liquid samples (Figure S9 and Table S10). Three metabolites were found in biosolid samples from both species of fungi. Metabolic reactions observed were demethylation, hydroxylation, *N*-oxidation, hydrogenation, and dehydration. The tertiary amine group on amitriptyline was metabolized by both fungi via demethylation (AMI-264) or *N*-oxide formation (AMI-310-enol, AMI-310-ketone and AMI-312); tertiary amine demethylation and *N-*oxide formation have been reported transformations in biodegradation studies of other compounds.^39,45^ The addition of an oxygen on the aliphatic chain (AMI-294 and AMI-280) also occurred in samples exposed to both species of white-rot fungi. AMI-264 (nortriptyline) is a known CYP450 oxidation product of amitriptyline in mammalian metabolism but has not been identified as a white-rot fungal metabolite;^46^ all other metabolites have not been reported before.

#### Trazodone

In the liquid media samples spiked with trazodone, five metabolites were detected for both fungi, two were found only in *T. versicolor* samples, and one was exclusive to *P. ostreatus* (Figure S12 and Table S13). Identification of metabolites indicates the relevance of both oxidation (hydroxylation, ketone addition) and reduction (cleavage, oxygen elimination) reactions. Detection of TRA-388 and TRA-400 suggests that the addition of oxygen to the piperazine structure is a common first metabolic step. Even though previous studies investigating the mammalian metabolism of trazodone observed oxidation on either the chlorinated ring or the pyridine (such as TRA-406), the MS2 fragmentation spectra from TRA-388, TRA-400, TRA-402, and TRA-404 all support oxidation on the piperazine ring. A similar metabolism pathway has been shown on the piperazine structure of ketoconazole in mammals.^47^ Additionally, the cleavage of the piperazine ring has been observed in ofloxacin degradation by *T. versicolor*.^48^ Of the eight compounds detected, only the presence of TRA-406, which indicates hydroxylation on the pyridine, is a known transformation product of trazodone in human metabolism.^49^

#### Carbamazepine

Six metabolites of carbamazepine were identified; all were present in samples exposed to *T. versicolor* and four were found in *P. ostreatus* samples (Figure S13 and Table S14). Of all identified metabolites, only two products (CBZ-253 and CBZ-271) were found in biosolids. The identification of metabolites indicates the relevance of hydroxylation, epoxidation, hydrogenation, and formamide cleavage reactions. CBZ-180 (acridine), CBZ-252 (carbamazepine-10,11-epoxide), and CBZ-271 (10,11-dihydroxycarbamazepine) have been previously reported metabolites of both *T. versicolor* and *P. ostreatus*.^30,50^ CBZ-194 (iminostilbene) is a known degradation product of carbamazepine by *T. versicolor*, and CBZ-251 is a metabolite reported from *P. ostreatus*.^30,50^ CBZ-239 (10,11-dihydrocarbamazepine), which was observed only in autoclaved fungal control samples, indicates potential abiotic hydrogenation in media.

#### Citalopram

All identified metabolites of citalopram degradation were observed in samples from both white-rot fungal species. Metabolism of the tertiary amine group was observed for all identified metabolites, which included *N*-oxide formation (CTL-341), demethylation (CTL-311), and formylation (CTL-339). CTL-311 (desmethylcitalopram) and CTL-341 are known products of oxidation in mammalian metabolism, and CTL-311 has additionally been found in liquid *P. ostreatus* biodegradation experiments.^51–53^ CTL-311 was only found in liquid media, while CTL-339 and CTL-341 were also identified in biosolid samples.

#### Sertraline

Three major sertraline metabolites were found (Figure S11 and Table S12). Isomers SER-322A/B were found in the liquid media of both fungi and measured in *P. ostreatus* samples grown on biosolids. SER-149 was only found in *P. ostreatus* biosolid samples. Metabolic reactions involved hydroxylation and dichlorobenzene cleavage. Kózka et al. proposed a structure where the oxidation by white-rot fungal enzymes occurred on the aromatic ring; however, the fragmentations of SER-322A/B support the oxidation of either the second or third carbon of the cyclohexane (Figures S32–S33).^51^

Despite parent compound degradation in both matrices, several of the identified metabolites were unique to either liquid media or biosolids. For example, trazodone metabolites were found in liquid media samples, but none were detected in biosolid samples, despite both fungal species exhibiting a greater than 75% removal. All trazodone metabolites have much lower predicted organic carbon partitioning coefficients (log *K*_OC_) than trazodone itself (Table S8) which indicates they are less likely to sorb onto biosolid surfaces and, therefore, are more bioavailable for fungi to degrade further into smaller compounds that may not have been detected by the analytical approach. Additionally, the presence of metabolites unique to biosolid samples suggests alternative metabolic pathways to those observed in liquid culture. Enzyme production and yield are influenced heavily by growth conditions and substrate, which is why studying degradation in realistic conditions is important for predicting large-scale fungal degradation.^54^

A few of the transformation products were present in abiotic (no fungi) or dead fungal controls. DVEN-237, CBZ-239, and FLX-196 were found only in autoclaved fungal controls. As these compounds were not also present in the abiotic controls, the dead fungal mass must be influencing the transformation. Fungal cells are covered with negatively charged mannoproteins that, perhaps, interact with parent compounds, transforming them, and then shedding them as the dead mass withers away over time.^55^ AMI-276, AMI-294, CBZ-194, and CTL-339 were present in abiotic, living, and dead fungal samples. This indicates the involvement of abiotic oxidation or reduction reactions. However, besides CBZ-194, the relative peak areas of the compounds in the living fungal samples are much greater than the controls, which indicates the importance of fungal metabolism for their formation.

For successful fungal treatment of biosolids, not only do the pollutant concentrations need to decrease in the contaminated matrix but also the metabolites should be less harmful than their parent compound. In order to assess whether the fungal metabolites are less toxic than their parent compounds, we utilized the EPA’s CompTox Chemical Dashboard and calculated quality-adjusted average hazard scores for the 41 metabolites and their parent compounds (Tables 2 and S17). It is important to note that the majority of results were predicted using QSAR models, which increases the uncertainty of their toxicity. We addressed this by using a quality-adjusted hazard score, which takes into account the higher uncertainty associated with predicted hazard values. However, many of the metabolites found are not well-studied or are novel, so it is not surprising that the majority of their hazard scores are based on *in silico* QSAR modeling. All metabolites from desvenlafaxine, trazodone, citalopram, sertraline, and fluoxetine had quality-adjusted average hazard scores lower than that of their parent compound. One metabolite each of amitriptyline (AMI-276), carbamazepine (CBZ-180), and vilazodone (VIL-229) had greater quality-adjusted average hazard scores than their parent. Compared to amitriptyline, AMI-276 had far less hazard screening end points which is reflected in its low completeness score (Table S17). Similarly, the low hazard and completeness scores of vilazodone make it difficult to compare to VIL-229; only one of the ten searched toxicity end points returned a value for vilazodone. CBZ-180 (acridine) had a greater quality-adjusted average hazard score than carbamazepine and was the only metabolite to have a “very high” genotoxicity/mutagenicity score. Overall, the majority of the metabolites have lower predicted toxicity than their parent compounds, which indicates that fungal metabolism of psychoactive pharmaceuticals results in detoxification.

### Limitations and Environmental Implications

Liquid degradation experiments are, in general, faster and cheaper than solid-state fermentation. However, a major shortcoming to many white-rot fungal degradation studies using liquid culture is that they only sample the concentration in the liquid media over time, which does not differentiate degradation and sorption to the fungal mass. To account for this, heat-killed control samples (autoclaved fungi) can be used to discern between active absorption across fungal cell walls and adsorption onto the surface of fungal cell walls. Indeed, in this study, the final concentrations for some compounds in liquid media with living fungi were not different from the heat-killed controls. Amitriptyline, vilazodone, sertraline, and citalopram in *P. ostreatus* samples and sertraline and fluoxetine in *T. versicolor* samples appear to be removed from the liquid media at least partially by sorption to the fungal mass. These data can give an estimate to the compounds’ behaviors when exposed to active fungi. However, autoclaving uses steam heat to induce irreversible coagulation of the cells, and the physicochemical properties of the resulting fungal corpse are unknown, which increases the uncertainty of using autoclaved controls as models for living fungal biosorption. Future studies involving liquid culture should also extract the fungal mass to calculate each compound’s mass balance and determine true degradation. When investigating the degradation potential of white-rot fungi in a solid matrix (biosolids, sewage sludge, soil, and others), using solid-state fermentation culture provides a more realistic treatment condition and increases the confidence in the measured percent removal of these compounds. This study used autoclaved biosolids as a matrix for fungal growth in order to determine the maximum potential of fungal degradation of the psychoactive pharmaceuticals. However, biosolids are rich with native microbes, which can compete with white-rot fungi and potentially limit their growth. Thus, future studies should compare compound degradation by fungi grown on autoclaved biosolids and nonautoclaved biosolids to determine the effects that native microbes have on white-rot fungal treatment.

The results of the degradation experiments on biosolids over 60 days indicate that the concentration of the psychoactive compounds plateaued by day 40, which suggests that an incubation period of 2 months is sufficient for their degradation. Prior inoculation of the bulk substrate with white-rot fungi before mixing with biosolids would likely increase fungal degradation rates, as mycelial permeation in biosolids and enzymatic activity would be immediate. Future work should investigate multiple different classes of biosolid pollutants to determine time thresholds for degradation. The results further suggest a detoxification of the parent compounds as indicated by their metabolites’ overall lower quality-adjusted average hazard scores. Metabolites are also, on average, more bioavailable (lower log *K*_OC_) than their parent compound which means that they would be more accessible to soil microbes for further degradation. These results show a promising mycoaugmentation strategy for the degradation of biosolid-associated organic compounds.

## Conclusions

The use of white-rot fungi for the abatement of organic contaminants in solid matrices is advantageous because their extracellular enzymes are capable of degrading compounds that are recalcitrant to degradation by other microorganisms. Fungi can grow to penetrate dense solids, are ubiquitous in the environment, and are a low-cost strategy to degrade pollutants in solid matrices. The aim of this work was to determine whether white-rot fungi can degrade psychoactive compounds in biosolids before an environmental application. The results from this study highlight that *P. ostreatus* and *T. versicolor* are effective at degrading these compounds in biosolids, which is important as some of them have been previously reported to persist in soils. Indeed, apart from fluoxetine exposed to *P. ostreatus*, the concentration of each compound decreased by over 50% in the biosolids when white-rot fungi were present. Trazodone and desvenlafaxine were almost completely degraded by both white-rot fungal species. In addition, this study compares psychoactive compound degradation by the fungi grown in liquid media and biosolids. Amitriptyline, lamotrigine, carbamazepine, and citalopram all exhibited significant differences in removal efficiency between liquid and biosolid cultures. Identification of fungal metabolites in both liquid and biosolid samples indicates that while some transformation products were consistent between both matrices, others were matrix-dependent and were only found in either biosolids or liquid culture samples. Similarly, while the majority of the metabolites were produced by both fungi, 18 were species dependent. Overall, this work demonstrates that white-rot fungi can remove organic contaminants adsorbed on biosolids and highlights the necessity of using relevant matrices to assess degradation potential.

## Supplementary Material



## Notes

Summary of psychoactive pharmaceutical physicochemical properties, detection in biosolids, and previously reported degradation by white-rot fungi; high-resolution mass spectrometry data analysis details; solid-state fermentation kinetic curves; liquid culture kinetic curves; physicochemical properties of proposed metabolites; proposed transformation pathways and kinetic curves of psychoactive pharmaceutical metabolites; hazard screening of parent and metabolites; and MS2 fragmentation spectra of metabolites (DOCX)
