# Supplementary material for: Magic Mushrooms? White-Rot Fungal Degradation of Psychoactive Pharmaceuticals in Biosolids
Source: ACS Environ Au. 2026 Jan 29;6(2):346–57. doi: 10.1021/acsenvironau.5c00258 (PMC13003354; doi:10.1021/acsenvironau.5c00258)
Supplement: Supplementary file 1 [file vg5c00258_si_001.docx]

Supporting Information for:

Magic Mushrooms? White-Rot Fungal Degradation of Psychoactive Pharmaceuticals in Biosolids

Kate Burgener ^1^, Carsten Prasse ^1,2,*^

^1^ Department of Environmental Health & Engineering, Johns Hopkins University, Baltimore, MD, 21218, USA

^2^ Risk Sciences and Public Policy Institute, Johns Hopkins Bloomberg School of Public Health, Baltimore, MD, 21205, USA

^*^ Address correspondence to cprasse1@jhu.edu

Number of pages: 51

Number of figures: 47

Number of tables: 17

**Table of Contents**

| Background ……………………………………………………………………………………….. | 3 |
| --- | --- |
| Data Analysis ………………….………………………………………………………………….. | 5 |
| Instrument Analysis ……………………………………………………...……………………….. | 6 |
| Solid-state fermentation results …………………………………………………….……………... | 8 |
| Liquid degradation results…………………………………………………………………………. | 9 |
| Metabolite results and proposed structures .………………………………………………………. | 16 |
| Metabolite hazard scores ………………………………………………………………………….. | 40 |
| Metabolite FISh diagrams ……………………………………………………………………........ | 41 |
| References ………………………………………………………………………………………… | 52 |

**Table S1. Detection and quantification of psychoactive pharmaceuticals in biosolids.** For each of the nine psychoactive pharmaceuticals studied, their previously quantified concentration in biosolids is listed.

| **Compound** | **Concentration in Biosolids (ng/g)** |
| --- | --- |
| Amitriptyline | 20.7^1^ |
| Lamotrigine | 31^2^ |
| Vilazodone | - |
| Desvenlafaxine | 47.3^3^ |
| Sertraline | 373.27 - 1,037^1,3^ |
| Trazodone | 90.2^1^ |
| Carbamazepine | 23.7 - 163^1,4,5^ |
| Citalopram | 81.9 - 232^1,3^ |
| Fluoxetine | 12.5 – 326.2^1,3,5^ |

**Table S2. Chemical and physical properties of psychoactive pharmaceuticals.** pKa and Log K_ow_ values are from Drugbank; unless otherwise cited, Log K_OC_ and safety hazards are from Pubchem. *Log K_OC_ was predicted with EPIsuite (https://episuite.dev/EpiWebSuite/#/).

| **Compound** | **Abbreviation** | **Common Name** | **Pharmaceutical use** | **CAS number** | **Monoisotopic mass** | **pKa** | **Log K_OC_** | **Log K_OW_** | **Safety Hazard** |
| --- | --- | --- | --- | --- | --- | --- | --- | --- | --- |
| Amitriptyline | AMI | Elavil | TCA | 549-18-8 | 277.183050 | 9.4 | 3.55 | 4.92 | Corrosive, Acute Toxic, Irritant, Health Hazard, Environmental Hazard |
| Lamotrigine | LTG | Lamictal | Anticonvulsant | 84057-84-1 | 255.007851 | 5.7 | 3.28 | 1.93 | Acute Toxic |
| Vilazodone | VIL | Viibryd | Antidepressant (SPARI) | 163521-12-8 | 441.216475 | 14.19/8.6 | Unknown | 4.21 |  |
| Desvenlafaxine | DVEN | Pristiq | SNRI | 93413-62.8 | 263.188529 | 10.11/8.87 | 3.23 | 2.6 | Irritant |
| Sertraline | SER | Zoloft | SSRI | 79617-96-2 | 305.073805 | 9.16 | 5.7^6^ | 5.51 | Irritant, Health Hazard |
| Trazodone | TRA | Desryel, Oleptro, Raldesy | SARI | 25332-39-2 | 371.151288 | 6.74 | 3.21* | 2.68 | Irritant |
| Carbamazepine | CBZ | Carbatrol, Carnexiv, Epitol, Equetro, Tegratol | Anticonvulsant | 298-46-4 | 236.094963 | 15.96/-3.8 | 2.71 | 2.77 | Irritant, Health Hazard |
| Citalopram | CTL | Celexa | SSRI | 59729-32-7 | 324.163792 | 9.78 | 5.63 | 3.76 | Irritant, Health Hazard, Environmental Hazard |
| Fluoxetine | FLX | Prozac, Sarafem, Symbyax | SSRI | 56296-78-7 | 309.134049 | 9.8 | 4.72^7^ | 4.05 | Corrosive, Acute Toxic, Health Hazard, Environmental Hazard |

| **Compound**  **Table S3. Previously reported degradation of psychoactive pharmaceuticals by white-rot fungi.** Degradation by white-rot fungi has been demonstrated for five of the compounds studied. Different species, growth conditions, and sample times all affect the amount removed. | **White-rot fungi** | **% Removed** | **Initial concentration** | **Growth Media** | **Last Sample** | **Citation** |
| --- | --- | --- | --- | --- | --- | --- |
| **Amitriptyline** | - | - | - | - | - | - |
| **Lamotrigine** | *P. ostreatus* | 96% | 1 and 10 µg/L | Glucose Peptone Media | 20 days | ^8^ |
| **Vilazodone** | - | - | - | - | - | - |
| **Desvenlafaxine** | *P. ostreatus* | 100% | 5 mg/L | Synthetic medium | 15 days | ^9^ |
|  | *T. versicolor* | 100% | 5 mg/L | Synthetic medium | 15 days | ^9^ |
|  | *G. lucidium* | 100% | 5 mg/L | Synthetic medium | 15 days | ^9^ |
| **Sertraline** | *P. ostreatus* | 45.5% | 20µg/mL | Corn steep liquor | 48hr | ^10^ |
|  | P. ostreatus NRRL 2366 strain | 93.7% | 2.5 µg/mL and 100 ng/mL | Sabouraud Dextrose Broth | 96 hr | ^11^ |
|  | A. mellea NBRC 7037 | 31% | 2.5 µg/mL and 100 ng/mL | Sabouraud Dextrose Broth | 96 hr | ^11^ |
|  | P. *[chrysosporium](https://www.sciencedirect.com/topics/pharmacology-toxicology-and-pharmaceutical-science/chrysosporium)* IFO 31249 | 44% | 2.5 µg/mL and 100 ng/mL | Sabouraud Dextrose Broth | 96 hr | ^11^ |
| **Trazodone** | - | - | - | - | - | - |
| **Carbamazepine** | *Bjerkandera adusta* (ATTC 90940) | 100% | 1 mg/L | Kirk Medium | 14 days | ^12^ |
|  | *Phanerochaete chrysosporium* (ATTC 24725) | 100% | 1 mg/L | Kirk Medium | 14 days | ^12^ |
|  | *Bjerkandera* sp. R1, a fungus isolated in a Chilean forest | 100% | 1 mg/L | Kirk Medium | 14 days | ^12^ |
|  | *T. versicolor* | 57% | 10 mg/L | Kirk Medium | 7 days | ^13^ |
|  | P. chrysosporium ME-446 | 0% | 10mg/L | Malt extract medium | 7 days | ^13^ |
|  | G. lucidum (Leysser) Karsten FP-58537-Sp | 46% | 10mg/L | Kirk Medium | 7 days | ^13^ |
|  | I. lacteus (AX1) | 0% | 10mg/L | Kirk Medium | 7 days | ^13^ |
|  | *P. ostreatus PC9* | 99% | 8.8 mg/L | Glucose peptone media | 25 days | ^14^ |
|  | *P. ostreatus* Florida F6 | 62% | 10 mg/L | Glucose peptone media | 30 days | ^15^ |
|  | *P. ostreatus* Florida N001 | 48% | 10 mg/L | Glucose peptone media | 30 days | ^15^ |
|  | *P. ostreatus* PC9 | 99% | 1 µg/L | Glucose peptone media | 30 days | ^15^ |
| **Citalopram** | *P. ostreatus* | 0% | 20µg/mL | Corn steep liquor | 48 hr | ^10^ |
|  | P. ostreatus NRRL 2366 strain | 50% | 2.5 µg/mL and 100 ng/mL | Sabouraud Dextrose Broth | 96 hr | ^11^ |
|  | A. mellea NBRC 7037 | 0% | 2.5 µg/mL and 100 ng/mL | Sabouraud Dextrose Broth | 96 hr | ^11^ |
|  | P. [chrysosporium](https://www.sciencedirect.com/topics/pharmacology-toxicology-and-pharmaceutical-science/chrysosporium) IFO 31249 | 3% | 2.5 µg/mL and 100 ng/mL | Sabouraud Dextrose Broth | 96 hr | ^11^ |
|  | *Bjerkandera adusta* (ATTC 90940) | 100% | 1 mg/L | Kirk Medium | 14 days | ^12^ |
|  | *Phanerochaete chrysosporium* (ATTC 24725) | 100% | 1 mg/L | Kirk Medium | 14 days | ^12^ |
|  | *Bjerkandera* sp. R1, a fungus isolated in a Chilean forest | 100% | 1 mg/L | Kirk Medium | 14 days | ^12^ |
| **Fluoxetine** | *P. ostreatus* | 20% | 20µg/mL | Corn steep liquor | 48 hr | ^10^ |
|  | P. ostreatus NRRL 2366 strain | 0% | 2.5 µg/mL and 100 ng/mL | Sabouraud Dextrose Broth | 96 hr | ^11^ |
|  | A. mellea NBRC 7037 | 17% | 2.5 µg/mL and 100 ng/mL | Sabouraud Dextrose Broth | 96 hr | ^11^ |
|  | P. [chrysosporium](https://www.sciencedirect.com/topics/pharmacology-toxicology-and-pharmaceutical-science/chrysosporium) IFO 31249 | 23% | 2.5 µg/mL and 100 ng/mL | Sabouraud Dextrose Broth | 96 hr | ^11^ |
|  | *Bjerkandera adusta* (ATTC 90940) | 23% | 1 mg/L | Kirk Medium | 14 days | ^12^ |
|  | *Phanerochaete chrysosporium* (ATTC 24725) | 25% | 1 mg/L | Kirk Medium | 14 days | ^12^ |
|  | *Bjerkandera* sp. R1, a fungus isolated in a Chilean forest | 46% | 1 mg/L | Kirk Medium | 14 days | ^12^ |

**Text S1. Methods**

**Extraction**

Biosolid Experiments

Five grams of each sample were weighed out in 50 mL Falcon tubes. 100 µL of internal standard were added to each sample and left to dry for one hour. 20 mL of methanol were added to each sample which was vortexed and then sonicated for 30 minutes. Samples were centrifuged at 4˚C for 20 minutes at 3,000 x g. 3 mL of supernatant were removed and an additional 10 mL of methanol were added to the samples. Each sample was vortexed, sonicated, and centrifuged again; 5 mL of supernatant were then taken from the centrifuged samples. 10 additional mL of methanol were added to samples which were vortexed, sonicated, and centrifuged a third time. 10 mL of supernatant were taken from the samples. The 18 mL of supernatant were dried down with a gentle stream of air to 10 mL which was then centrifuged at 3,000 x g at 4˚C for 15 minutes to remove any additional solids. Samples were dried down to 1 mL of supernatant, after which 1 mL of MilliQ water was added. Samples were dried down to a final volume of 1 mL and then centrifuged a final time for at 20,000 x g at 4˚C for 15 minutes. The samples were then filtered with 0.2 µm PTFE filter into autosampler vials to be analyzed with liquid chromatography high-resolution mass spectrometry (LC-HRMS).

Liquid Experiments

The sampled growth media was centrifuged at 20,000 x g at 4˚C for 10 minutes. 500 µL of supernatant were added to 500 µL of MilliQ water and 100 ng internal standard. Samples were then filtered with 0.2 µm PTFE filter into autosampler vials to be analyzed with LC-HRMS.

**Instrument Analysis and Quantification**

Chromatographic separation was performed with an UltiMate 3000 RSLCnano system (Thermo Scientific) with a Synergi Hydro-RP column (150 mm x 1mm, 4µm; Phenomenex, Rorance, CA) and a KrudKatcher ULTRA in-line filter. Aqueous mobile phase was 1mM ammonium fluoride in MQW and organic mobile phase was 0.1% formic acid in methanol. Samples were analyzed with heated electrospray ionization (H-ESI) using a Q-Exactive HF Orbitrap HRMS (Thermo Scientific) with data acquired in full-scan/data-dependent MS2 with positive and negative polarity switching.

**Table S4. Liquid chromatography conditions for separation of extracted samples.** Column temperature was 45˚C, autosampler temperature was 10˚C, and injection volume was 5µL.

| Time (min) | Solvent A (%) | Solvent B (%) | Flow (µL/min) |
| --- | --- | --- | --- |
| 0 | 90 | 10 | 75 |
| 1 | 90 | 10 | 75 |
| 16 | 2 | 98 | 75 |
| 20 | 2 | 98 | 75 |
| 20.1 | 2 | 98 | 150 |
| 22 | 2 | 98 | 150 |
| 22.1 | 90 | 10 | 150 |
| 29.4 | 90 | 10 | 150 |
| 29.5 | 90 | 10 | 75 |
| 30 | 90 | 10 | 75 |

**Table S5. Mass spectrometer H-ESI source and full scan/dd-MS2 settings.**

| **Heated-electrospray ionization source settings** | | | |
| --- | --- | --- | --- |
| Sheath gas | | 20 | |
| Aux gas | | 5 | |
| Sweep gas | | 0 | |
| Spray voltage (\|kV\|) | | 3.0 (pos), 2.5 (neg) | |
| Capillary temperature (℃) | | 300 | |
| S-lens RF level | | 60 | |
| Aux gas heater temperature (℃) | | 150 | |
|  | | | |
| **Full scan/data dependent-MS^2^ settings** | | | |
|  | Full MS | dd-MS^2^ | dd Settings |
| Runtime (min) | 2.0-28.0 |  |  |
| Resolution | 60,000 | 15,000 | - |
| AGC target | 3E+06 | 2E+05 | - |
| Max IT (ms) | 100 | 50 | - |
| Scan range (m/z) | 100-1000 | - | - |
| Loop Count | - | 10 | - |
| Isolation window (m/z) | - | 0.7 | - |
| NCE stepped collision energy | - | 30, 65, 100 | - |
| Minimum AGC target | - | - | 1E+04 |
| Intensity threshold | - | - | 2E+05 |
| Apex trigger (s) | - | - | 1-3 |
| Exclude isotopes | - | - | On |
| Dynamic exclusion (s) | - | - | 10 |

**Table S6. Compound Discoverer data workflow settings.**

| Node | Parameter |  |
| --- | --- | --- |
| Align Retention Times | Alignment model | Adaptive curve |
|  | Maximum Shift (min) | 1 |
|  | Mass Tolerance | 5 ppm |
| Detect Compounds | Min Peak Intensity | 250000 |
|  | Chromatographic S/N Threshold | 5 |
|  | Ions | [2M+H]+1, [2M+K]+1, [2M+Na]+1, [2M-H]-1, [M+H]+1, [M+K]+1, [2M+Na]+1,  [M-H]-1 |
| Group Compounds | RT tolerance (min) | 0.75 |
|  | Area Contribution | 3 |
|  | CV Contribution | 0 |
|  | FWHM to Base Contribution | 7 |
|  | Jaggedness Contribution | 5 |
|  | Modality Contribution | 5 |
|  | Zig-Zag Index Contribution | 5 |
|  | Peak Rate Threshold | 6 |
|  | Number of Files | 3 |
| Fill Gaps | Mass Tolerance | 5 ppm |
|  | S/N Threshold | 1.5 |
| Apply QC Correction | Min QC Coverage (%) | 50 |
|  | Max QC Area RSD (%) | 50 |
|  | Max Corrected QC Area RSD (%) | 30 |
| Mark Background | Max Sample/Blank | 5 |
|  | Max Blank/Sample | 0 |
| Normalize Areas | Normalization Type | Constant Median |

Quantification of the psychoactive pharmaceuticals was achieved with matrix match calibration curves in which the calibrators (0, 10, 25, 50, 100, 250, 500, and 1,000 ng/g (or ng/mL)) were spiked into liquid growth media or solid wheat/biosolids matrix and extracted using the same procedure described above. Internal standards were used to account for extraction and mass spectrometry discrepancies. Matching deuterated standards were used for amitriptyline, lamotrigine, vilazodone, desvenlafaxine, sertraline, carbamazepine, and fluoxetine; carbamazepine-d10 was also used as an internal standard for trazodone, and fluoxetine-d5 was used as a standard for citalopram.

**A**

**B**

**C**

**D**

**E**

**F**

**G**

**H**

**Figure S1. Biodegradation of psychoactive compounds sorbed onto biosolids exposed to white-rot fungi *P. ostreatus* or *T. versicolor*.** Biosolids were spiked with psychoactive compounds in three groups: amitriptyline (AMI), vilazodone (VIL), and lamotrigine (LTG) (group 1), desvenlafaxine (DVEN), sertraline (SER), and trazodone (TRA) (group 2), and carbamazepine (CBZ), citalopram (CTL), and fluoxetine (FLX) (group 3). Each group of compounds in biosolids were exposed to either *P. ostreatus* or *T. versicolor* for 60 days. Samples were sacrificially sampled in triplicate at each of the eight time points and the concentration of each compound was quantified. Controls are biosolids spiked with the compounds without the presence of any fungi. Symbols represent the mean concentration in ng/g and error bars are the standard deviations of the triplicate samples.

**A**

**B**

**C**

**D**

**E**

**Figure S2. Quantified results from Group 1 biodegradation by *P. ostreatus* in liquid culture.** Amitriptyline (AMI) (**A**), lamotrigine (LTG) (**B**), and vilazodone (VIL) (**C**) were spiked into *P. ostreatus* cultures at an initial concentration of 1,000 ng/mL. Samples were sacrificially sampled in triplicate at each of the six timepoints over 20 days of exposure. Abiotic Controls were glucose peptone growth media spiked with Group 1 compounds in the absence of fungi, Dead P. ostreatus controls were Group 1 compounds exposed to autoclaved fungi, and P. ostreatus Controls were fungi culture not spiked with Group 1 compounds. In addition to collecting growth media, the pH of the liquid media (**D**) and the mass of the fungi (**E**) were recorded. Symbols represent the mean value at each time point and error bars are the standard deviation.

**A**

**B**

**D**

**C****c**

**E****c**

**Figure S3. Quantified results from Group 2 biodegradation by *P. ostreatus* in liquid culture.** Desvenlafaxine (DVEN) (**A**), sertraline (SER) (**B**), and trazodone (TRA) (**C**) were spiked into *P. ostreatus* cultures at an initial concentration of 1,000 ng/mL. Samples were sacrificially sampled in triplicate at each of the six timepoints over 20 days of exposure. Abiotic Controls were growth media spiked with Group 2 compounds in the absence of fungi, Dead P. ostreatus controls were Group 2 compounds exposed to autoclaved fungi, and P. ostreatus Controls were fungi culture not spiked with Group 2 compounds. In addition to collecting growth media, the pH of the liquid media (**D**) and the mass of the fungi (**E**) were recorded. Symbols represent the mean value at each time point and error bars are the standard deviation.

**A****c**

**B****c**

**C****c**

**D****c**

**E****c**

**Figure S4. Quantified results from Group 3 biodegradation by *P. ostreatus* in liquid culture.** Carbamazepine (CBZ) (**A**), citalopram (CTL) (**B**), and fluoxetine (FLX) (**C**) were spiked into *P. ostreatus* cultures at an initial concentration of 1,000 ng/mL. Samples were sacrificially sampled in triplicate at each of the six timepoints over 20 days of exposure. Abiotic Controls were growth media spiked with Group 3 compounds in the absence of fungi, Dead P. ostreatus controls were Group 3 compounds exposed to autoclaved fungi, and P. ostreatus Controls were fungi culture not spiked with Group 3 compounds. In addition to collecting growth media, the pH of the liquid media (**D**) and the mass of the fungi (**E**) were recorded. Symbols represent the mean value at each time point and error bars are the standard deviation.

**A****c**

**B****c**

**C****c**

**D****c**

**E****c**

**Figure S5. Quantified results from Group 1 biodegradation by *T. versicolor* in liquid culture.** Amitriptyline (AMI) (**A**), lamotrigine (LTG) (**B**), and vilazodone (VIL) (**C**) were spiked into *T. versicolor* cultures at an initial concentration of 1,000 ng/mL. Samples were sacrificially sampled in triplicate at each of the six timepoints over 20 days of exposure. Abiotic Controls were growth media spiked with Group 1 compounds in the absence of fungi, Dead T. versicolor controls were Group 1 compounds exposed to autoclaved fungi, and T. versicolor Controls were fungi culture not spiked with compounds. In addition to collecting growth media, the pH of the liquid media (**D**) and the mass of the fungi (**E**) were recorded. Symbols represent the mean value at each time point and error bars are the standard deviation.

**A****c**

**B****c**

**D****c**

**C****c**

**E****c**

**Figure S6. Quantified results from Group 2 biodegradation by *T. versicolor* in liquid culture.** Desvenlafaxine (DVEN) (**A**), sertraline (SER) (**B**), and trazodone (TRA) (**C**) were spiked into *T. versicolor* cultures at an initial concentration of 1,000 ng/mL. Samples were sacrificially sampled in triplicate at each of the six timepoints over 20 days of exposure. Abiotic Controls were growth media spiked with Group 2 compounds in the absence of fungi, Dead T. versicolor controls were Group 2 compounds exposed to autoclaved fungi, and T. versicolor Controls were fungi culture not spiked with compounds. In addition to collecting growth media, the pH of the liquid media (**D**) and the mass of the fungi (**E**) were recorded. Symbols represent the mean value at each time point and error bars are the standard deviation.

**A****c**

**B****c**

**D****c**

**C****c**

**E****c**

**Figure S7. Quantified results from Group 3 biodegradation by *T. versicolor* in liquid culture.** Carbamazepine (CBZ) (**A**), citalopram (CTL) (**B**), and fluoxetine (FLX) (**C**) were spiked into *T. versicolor* cultures at an initial concentration of 1,000 ng/mL. Samples were sacrificially sampled in triplicate at each of the six timepoints over 20 days of exposure. Abiotic Controls were growth media spiked with Group 3 compounds in the absence of fungi, Dead T. versicolor controls were Group 3 compounds exposed to autoclaved fungi, and T. versicolor Controls were fungi culture not spiked with compounds. In addition to collecting growth media, the pH of the liquid media (**D**) and the mass of the fungi (**E**) were recorded. Symbols represent the mean value at each time point and error bars are the standard deviation.

**Figure S8. Psychoactive pharmaceutical removal in liquid culture and on biosolids when exposed to white-rot fungi.** The final removal efficiency for liquid and solid culture were compared for all psychoactive pharmaceutical compounds exposed to *P. ostreatus* or *T. versicolor*. Amitriptyline (AMI), lamotrigine (LTG), desvenlafaxine (DVEN), sertraline (SER), trazodone (TRA), carbamazepine (CBZ), citalopram (CTL), and fluoxetine (FLX) were all quantified in both matrices while vilazodone (VIL) was not detected in biosolid samples. Differences in percent removal between liquid and biosolids demonstrate the importance of performing degradation experiments with relevant matrices. Data values are means and error bars are standard deviations.

**Table S7. Proposed metabolites found in liquid and solid fungal degradation experiments.** Metabolite properties were measured including the m/z of their most abundant MS2 fragmentation. Confidence level scores were given based on matches to known compound fragmentation in spectral libraries and FISh scores. Also recorded for each metabolite is if it was a predicted product of CYP450 or environmental metabolism by Biotransformer 3.0.

| **Metabolite** | **Common Name** | **m/z** | **Molecular Mass** | **Formula** | **RT (min)** | **5 Most Abundant Fragments** | | | | | **Confidence Level** | **Predicted TP** | **Previously Detected TP Mass in WRF Biodeg Experiments** |
| --- | --- | --- | --- | --- | --- | --- | --- | --- | --- | --- | --- | --- | --- |
| AMI-264 | Nortriptyline | 264.17435 | 263.16707 | C19H21N | 14.94 | 91.055 | 105.070 | 233.132 | 117.070 | 264.174 | 2 | Y |  |
| AMI-276 | Cyclobenzaprine | 276.17417 | 275.16689 | C20H21N | 14.08 | 58.066 | 276.175 | 84.081 | 215.086 | 231.116 | 2 | N |  |
| AMI-280 |  | 280.16909 | 279.16182 | C19H21NO | 14.55 | 219.117 | 237.127 | 178.078 | 204.094 | 191.085 | 3 | Y |  |
| AMI-292 |  | 292.16892 | 291.16164 | C20H21NO | 13.96 | 58.066 | 292.167 | 91.055 | 204.093 | 72.045 | 3 | Y |  |
| AMI-294 |  | 294.18481 | 293.17753 | C20H23NO | 14.03 | 58.066 | 91.055 | 294.186 | 72.081 | 178.078 | 3 | Y |  |
| AMI-310A |  | 310.17954 | 309.17224 | C20H23NO2 | 11.47 | 310.180 | 58.066 | 191.086 | 178.078 | 205.101 | 3 | Y |  |
| AMI-310B |  | 310.17952 | 309.17224 | C20H23NO2 | 12.42 | 310.180 | 58.066 | 191.086 | 91.055 | 178.078 | 3 | Y |  |
| AMI-312 |  | 312.19508 | 311.18781 | C20H25NO2 | 12.02 | 58.066 | 294.184 | 312.196 | 178.077 | 91.055 | 3 | Y |  |
| VIL-229 |  | 229.09684 | 228.08982 | C13H12N2O2 | 11.68 | 169.040 | 211.087 | 141.045 | 143.060 | 69.034 | 3 | Y |  |
| SER-149 | 1-tetralol | 149.09605 | 148.08878 | C10H12O | 14.97 | 149.097 | 91.055 | 93.070 | 131.085 | 79.055 | 3 | N | ^11^ |
| SER-322A |  | 322.07559 | 321.0681 | C17H17Cl2NO | 13.00 | 238.054 | 202.078 | 273.023 | 203.086 | 115.054 | 3 | Y | ^16^ |
| SER-322B |  | 322.07559 | 321.06832 | C17H17Cl2NO | 14.01 | 238.054 | 202.077 | 273.022 | 203.085 | 322.201 | 3 | Y | ^16^ |
| DVEN-115 | 1-methylcyclohexanol | 115.11198 | 114.1047 | C7H14O | 9.86 | 55.054 | 69.071 | 97.102 | 115.055 | 53.039 | 3 | N |  |
| DVEN-129 | 4-hydroxy-4-methylcyclohexanone | 129.09106 | 128.08378 | C7H12O2 | 10.10 | 67.070 | 83.086 | 55.055 | 87.081 | 111.081 | 3 | N |  |
| DVEN-232 |  | 232.16946 | 231.16197 | C15H21NO | 16.47 | 91.055 | 67.055 | 232.169 | 79.055 | 176.107 | 3 | N |  |
| DVEN-234 |  | 234.18507 | 233.17779 | C15H23NO | 18.72 | 234.184 | 107.086 | 91.055 | 79.055 | 150.091 | 3 | N |  |
| DVEN-237 |  | 237.1482 | 236.14092 | C14H20O3 |  | 57.071 | 81.070 | 85.065 | 109.065 | 219.138 | 3 | Y |  |
| DVEN-250 | N,O-didesmethylvenlafaxine | 250.17999 | 249.17271 | C15H23NO2 | 13.19 | 91.055 | 176.107 | 250.181 | 232.169 | 67.055 | 3 | Y |  |
| DVEN-251 |  | 251.12766 | 250.1204 | C14H18O4 | 10.71 | 91.055 | 205.122 | 79.055 | 187.117 | 233.117 | 3 | Y |  |
| DVEN-280 |  | 280.19025 | 279.18298 | C16H25NO3 | 6.49 | 280.191 | 58.066 | 55.019 | 67.055 | 81.070 | 3 | Y |  |
| DVEN-308 |  | 308.18501 | 307.17773 | C17H25NO4 | 10.58 | 58.066 | 290.175 | 151.039 | 308.186 | 245.117 | 3 | N |  |
| DVEN-336 |  | 336.17993 | 335.17266 | C18H25NO5 | 13.30 | 58.066 | 318.132 | 227.106 | 151.039 | 336.143 | 3 | N |  |
| TRA-194 | 2-(3-hydroxypropyl)-2H,3H-[1,2,4]triazolo[4,3-a]pyridine-3-one | 194.09227 | 193.08516 | C9H11N3O2 | 8.16 | 176.082 | 148.050 | 96.045 | 194.092 | 78.034 | 3 | Y |  |
| TRA-265 |  | 265.12927 | 264.12199 | C12H16N4O3 | 8.35 | 176.081 | 148.050 | 96.045 | 237.134 | 74.060 | 3 | N |  |
| TRA-346 |  | 346.1422 | 345.13492 | C17H20ClN5O | 11.73 | 176.082 | 148.061 | 96.045 | 219.124 | 346.143 | 3 | Y |  |
| TRA-388 |  | 388.15275 | 387.14547 | C19H22ClN5O2 | 13.48 | 176.082 | 148.051 | 96.045 | 205.109 | 388.154 | 3 | Y |  |
| TRA-400 |  | 400.11634 | 399.10907 | C19H18ClN5O3 | 13.30 | 176.082 | 96.045 | 148.051 | 209.048 | 265.074 | 3 | N |  |
| TRA-402 |  | 402.13196 | 401.12468 | C19H20ClN5O3 | 13.21 | 356.127 | 176.082 | 148.051 | 96.045 | 182.037 | 3 | N |  |
| TRA-404 |  | 404.14774 | 403.14046 | C19H22ClN5O3 | 11.01 | 176.082 | 148.051 | 96.045 | 154.042 | 368.128 | 3 | Y |  |
| TRA-406 | Triazolopyridinone dihydrodiol | 406.16318 | 405.1559 | C19H24ClN5O3 | 10.31 | 406.164 | 136.051 | 210.087 | 164.046 | 82.029 | 3 | N |  |
| CBZ-180 | Acridine | 180.08062 | 179.07362 | C13H9N | 12.66 | 180.081 | 85.029 | 69.034 | 127.039 | 145.049 | 2 | N | ^14^ |
| CBZ-194 | Iminostilbene | 194.09612 | 193.08919 | C14H11N | 14.30 | 194.096 | 193.089 | 179.073 | 167.072 | 91.054 | 2 | N |  |
| CBZ-239 | 10,11-dihydrocarbamazepine | 239.11709 | 238.10981 | C15H14N2O |  | 239.118 | 194.096 | 180.081 | 91.054 | 225.044 | 2 | Y |  |
| CBZ-251 |  | 251.08101 | 250.07375 | C15H10N2O2 | 12.87 | 180.080 | 251.081 | 208.075 | 91.054 | 178.064 | 3 | N | ^14^ |
| CBZ-253 | Carbamazepine 10,11-epoxide | 253.09671 | 252.08943 | C15H12N2O2 | 12.66 | 180.081 | 210.091 | 254.081 | 182.097 | 167.073 | 2 | Y | ^14^ |
| CBZ-271 |  | 271.10735 | 270.10007 | C15H14N2O3 | 11.89 | 180.081 | 210.092 | 254.081 | 182.096 | 167.073 | 3 | N | ^14^ |
| CTL-311 | Desmethylcitalopram | 311.15456 | 310.14729 | C19H19FN2O | 12.84 | 109.045 | 262.101 | 116.049 | 311.153 | 293.144 | 2 | Y | ^16^ |
| CTL-339 |  | 339.14971 | 338.14312 | C20H19FN2O2 | 12.40 | 339.149 | 258.070 | 276.081 | 261.057 | 58.066 | 3 | Y | ^16^ |
| CTL-341 |  | 341.1654 | 340.15877 | C20H21FN2O2 | 11.78 | 109.045 | 305.145 | 58.066 | 238.065 | 323.155 | 3 | Y | ^16^ |
| FLX-178 |  | 178.08585 | 177.07857 | C10H11NO2 | 9.54 | 160.076 | 132.081 | 115.054 | 178.086 | 142.065 | 3 | N | ^16^ |
| FLX-196 |  | 196.09684 | 195.08956 | C10H13NO3 | 14.10 |  |  |  |  |  | 4 | N | ^16^ |

| **Metabolite** | **Log K_OW_** | **Log K_OC_** | **Log D5.5** | **Log D7.4** |
| --- | --- | --- | --- | --- |
| AMI-264 | **4.51** | 5.33 | 0.489 | 1.74 |
| AMI-276 | 4.73 | 5.31 | 1.35 | 2.92 |
| AMI-280 | 3.85 | 4.23 |  |  |
| AMI-292 | 3.57 | 4.39 |  |  |
| AMI-294 | 4.06 | 4.20 |  |  |
| AMI-310 (enol) | 1.00 | 4.35 |  |  |
| AMI-310 (ketone) | 0.73 | 4.54 |  |  |
| AMI-312 | 0.72 | 4.35 |  |  |
| VIL-229 | 0.59 | 1.31 |  |  |
| SER-148 | **1.98** | 2.08 | 2.16 | 2.16 |
| SER-322A | 3.75 | 4.13 |  |  |
| SER-322B | 4.30 | 4.10 |  |  |
| DVEN-115 | 2.09 | 1.21 | 1.80 | 1.80 |
| DVEN-129 | 0.04 | 0.29 | 0.72 | 0.72 |
| DVEN-232 | 3.93 | 4.41 |  |  |
| DVEN-234 | 4.01 | 4.41 |  |  |
| DVEN-237 | 2.68 | 2.12 |  |  |
| DVEN-250 | 2.51 | 3.27 | -1.39 | -0.211 |
| DVEN-251 | 2.13 | 2.01 |  |  |
| DVEN-280 | 2.24 | 3.36 |  |  |
| DVEN-308 | 3.45 | 2.31 |  |  |
| DVEN-336 | -1.94 | 1.47 |  |  |
| TRA-194 | 0.18 | 1.20 |  |  |
| TRA-265 | -1.61 | 1.00 |  |  |
| TRA-346 | 2.57 | 3.92 |  |  |
| TRA-388 | 1.67 | 2.89 |  |  |
| TRA-400 | -0.09 | 3.15 |  |  |
| TRA-402 | 1.16 | 2.24 |  |  |
| TRA-404 | 0.24 | 1.74 |  |  |
| TRA-406 | 0.76 | 1.79 |  |  |
| CBZ-180 | **3.40** | **4.18** | 3.24 | 3.37 |
| CBZ-194 | 4.06 | 3.43 | 3.46 | 3.46 |
| CBZ-239 | 2.46 | 3.12 | 2.41 | 2.41 |
| CBZ-251 | 1.86 | 2.06 |  |  |
| CBZ-253 | 0.95 | 2.51 | 1.70 | 1.69 |
| CBZ-271 | -0.21 | 1.00 |  |  |
| CTL-311 | 3.53 | 4.47 | -0.114 | 0.923 |
| CTL-339 | 2.77 | 3.33 |  |  |
| CTL-341 | 0.85 | 4.59 |  |  |
| FLX-178 | -0.29 | 1.00 |  |  |
| FLX-196 | -0.79 | 1.14 |  |  |

**Table 8. Partitioning coefficients of metabolites.** The octanol-water and organic carbon-water partitioning coefficients for each metabolite were found with EPI Suite. Bolded numbers are experimental values while non-bolded numbers are estimated values.

**Table S9. Presence of metabolites in experimental matrices and conditions.** Detection of each metabolite is highlighted below. Green cells indicate that MS2 data was collected, yellow cells are those where MS2 data was not measured in that condition/matrix.

| Metabolite | *P. ostreatus* | | | | *T. versicolor* | | | |
| --- | --- | --- | --- | --- | --- | --- | --- | --- |
|  | Abiotic Liquid Culture | *P. ostreatus* Liquid Culture | Dead *P. ostreatus* Liquid Culture | *P. ostreatus* on biosolids | Abiotic Liquid Culture | *T. versicolor* Liquid Culture | Dead *T. versicolor* Liquid Culture | *T. versicolor* on biosolids |
| AMI-264 |  |  |  |  |  |  |  |  |
| AMI-276 |  |  |  |  |  |  |  |  |
| AMI-280 |  |  |  | No MS2 |  |  |  |  |
| AMI-292 |  |  |  |  |  |  |  |  |
| AMI-294 |  |  |  | No MS2 |  |  |  |  |
| AMI-310 (enol) |  |  |  |  |  |  |  |  |
| AMI-310 (ketone) |  |  |  |  |  |  |  | No MS2 |
| AMI-312 |  |  |  |  |  |  |  |  |
| VIL-229 |  |  |  |  |  |  |  |  |
| SER-148 |  |  |  |  |  |  |  |  |
| SER-322A |  |  |  | No MS2 |  |  |  |  |
| SER-322B |  |  |  |  |  |  |  |  |
| DVEN-115 |  |  |  |  |  |  |  |  |
| DVEN-129 |  |  |  |  |  |  |  |  |
| DVEN-232 |  |  |  |  |  |  |  |  |
| DVEN-234 |  |  |  |  |  |  |  |  |
| DVEN-237 |  |  |  |  |  |  |  |  |
| DVEN-250 |  |  |  |  |  |  |  |  |
| DVEN-251 |  |  |  |  |  |  |  |  |
| DVEN-280 |  |  |  |  |  |  |  |  |
| DVEN-308 |  |  |  |  |  |  |  |  |
| DVEN-336 |  |  |  |  |  |  |  |  |
| TRA-194 |  |  |  |  |  |  |  |  |
| TRA-265 |  |  |  |  |  |  |  |  |
| TRA-346 |  |  |  |  |  |  |  |  |
| TRA-388 |  |  |  |  |  |  |  |  |
| TRA-400 |  |  |  |  |  |  |  |  |
| TRA-402 |  |  |  |  |  |  |  |  |
| TRA-404 |  |  |  |  |  |  |  |  |
| TRA-406 |  |  |  |  |  |  |  |  |
| CBZ-180 |  |  |  |  |  | No MS2 |  |  |
| CBZ-194 |  |  |  |  |  |  |  |  |
| CBZ-239 |  |  |  |  |  |  |  |  |
| CBZ-251 |  |  |  |  |  |  |  |  |
| CBZ-253 |  |  |  |  |  |  |  |  |
| CBZ-271 |  |  |  |  |  |  |  | No MS2 |
| CTL-311 |  |  |  |  |  |  |  |  |
| CTL-339 |  |  |  |  |  |  |  |  |
| CTL-341 |  |  |  |  |  |  |  |  |
| FLX-178 |  |  |  |  |  |  |  |  |
| FLX-196 |  |  |  |  |  |  |  | No MS2 |


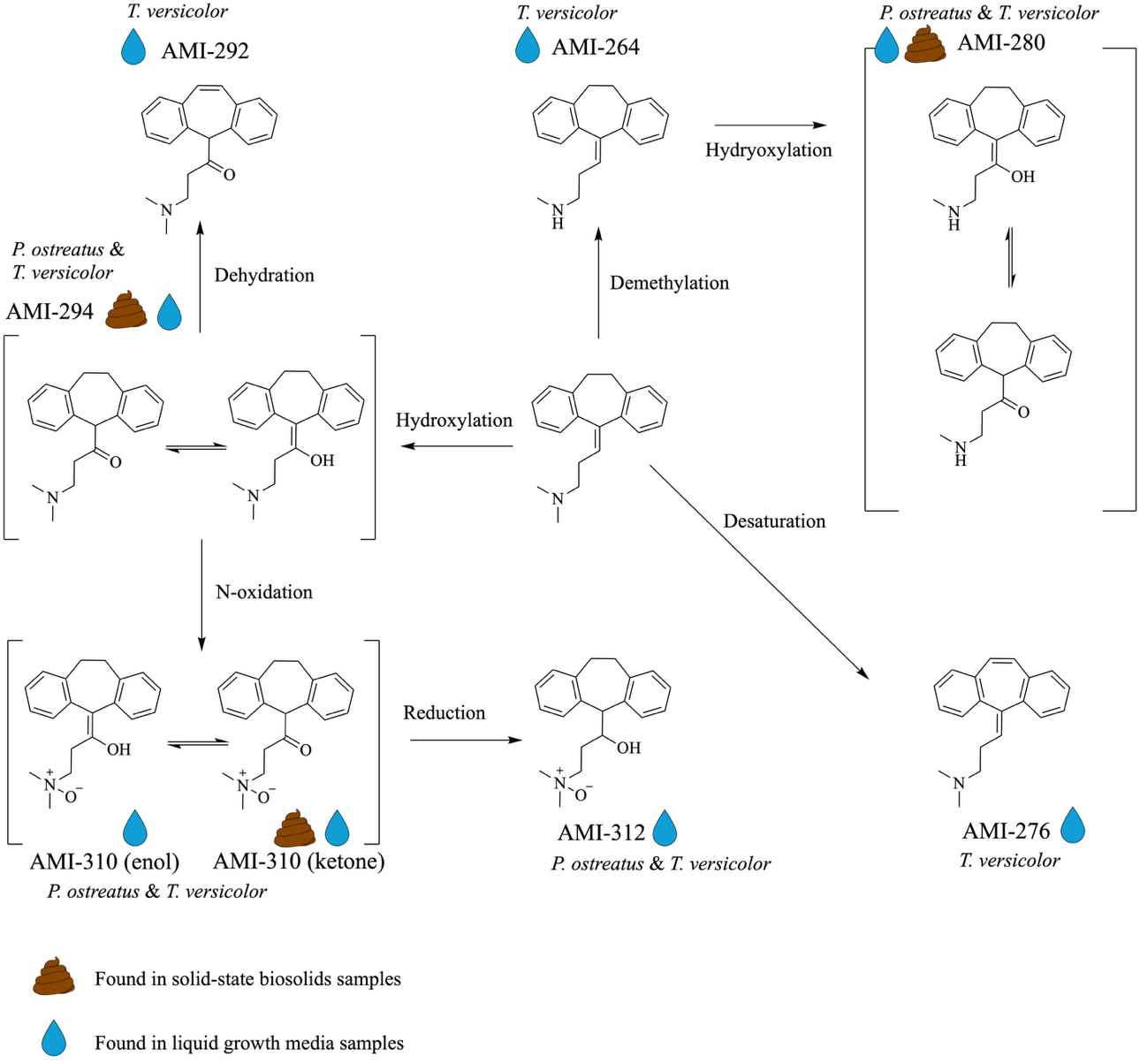


**Figure S9. Proposed transformation pathway of Amitriptyline (AMI) by white-rot fungi *P. ostreatus* and/or *T. versicolor*.** Metabolites AMI-264 (nortriptyline) and AMI-276 (cyclobenzaprine) matched to known spectra in ThermoFisher’s mzcloud library. Compounds AMI-264, AMI-280, AMI-310 (enol and ketone), and AMI-312 were predicted metabolites by Biotransformer 3.0. Compounds AMI-276, AMI-292, and AMI-293 have never been reported as metabolites. Bracketed structures indicate tautomers, where AMI-294 and AMI-280 peaks were indistinguishable so no determination between ketone and enol could be made.

**Table S10. Normalized peak areas of amitriptyline (AMI) metabolites overs time.**

| **TP** | ***P. ostreatus* liquid** | ***T. versicolor* liquid** | ***P. ostreatus* and/or *T. versicolor* solid** |
| --- | --- | --- | --- |
| AMI-264 |  |  |  |
| AMI-276 |  |  |  |
| AMI-280 |  |  |  |
| AMI-292 |  |  |  |
| AMI-294 |  |  |  |
| AMI-310 (enol) |  |  |  |
| AMI-310 (ketone) |  |  |  |
| AMI-312 |  |  |  |


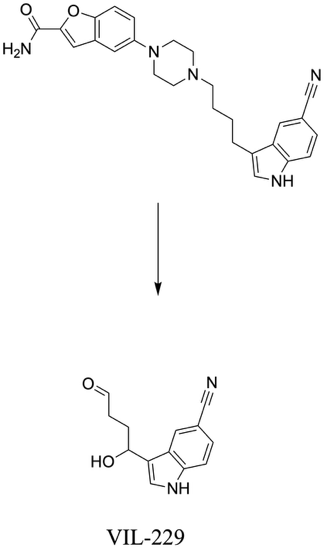


**B.**

**A.BB**

**Figure S10. Proposed transformation pathway of vilazodone (VIL) by white-rot fungi *T. versicolor*.** **A.** Proposed transformation of VIL found in liquid *T. versicolor* samples. Metabolites VIL-229 was a predicted metabolite of vilazodone by Biotransformer 3.0. **B.** Normalized peak area of VIL-229 over time when exposed to *T. versicolor*.

**Table S11. Normalized peak areas of desvenlafaxine metabolites overs time.** While DVEN-308 was detected in both matrices by both fungal species, many of the metabolites were matrix or species dependant.

| **TP** | ***P. ostreatus* liquid** | ***T. versicolor* liquid** | ***P. ostreatus* and/or *T. versicolor* solid** |
| --- | --- | --- | --- |
| DVEN-115 |  |  |  |
| DVEN-129 |  |  |  |
| DVEN-232 |  |  |  |
| DVEN-234 |  |  |  |
| DVEN-237 |  |  |  |
| DVEN-250 |  |  |  |
| DVEN-251 |  |  |  |
| DVEN-280 |  |  |  |
| DVEN-308 |  |  |  |
| DVEN-336 |  |  |  |


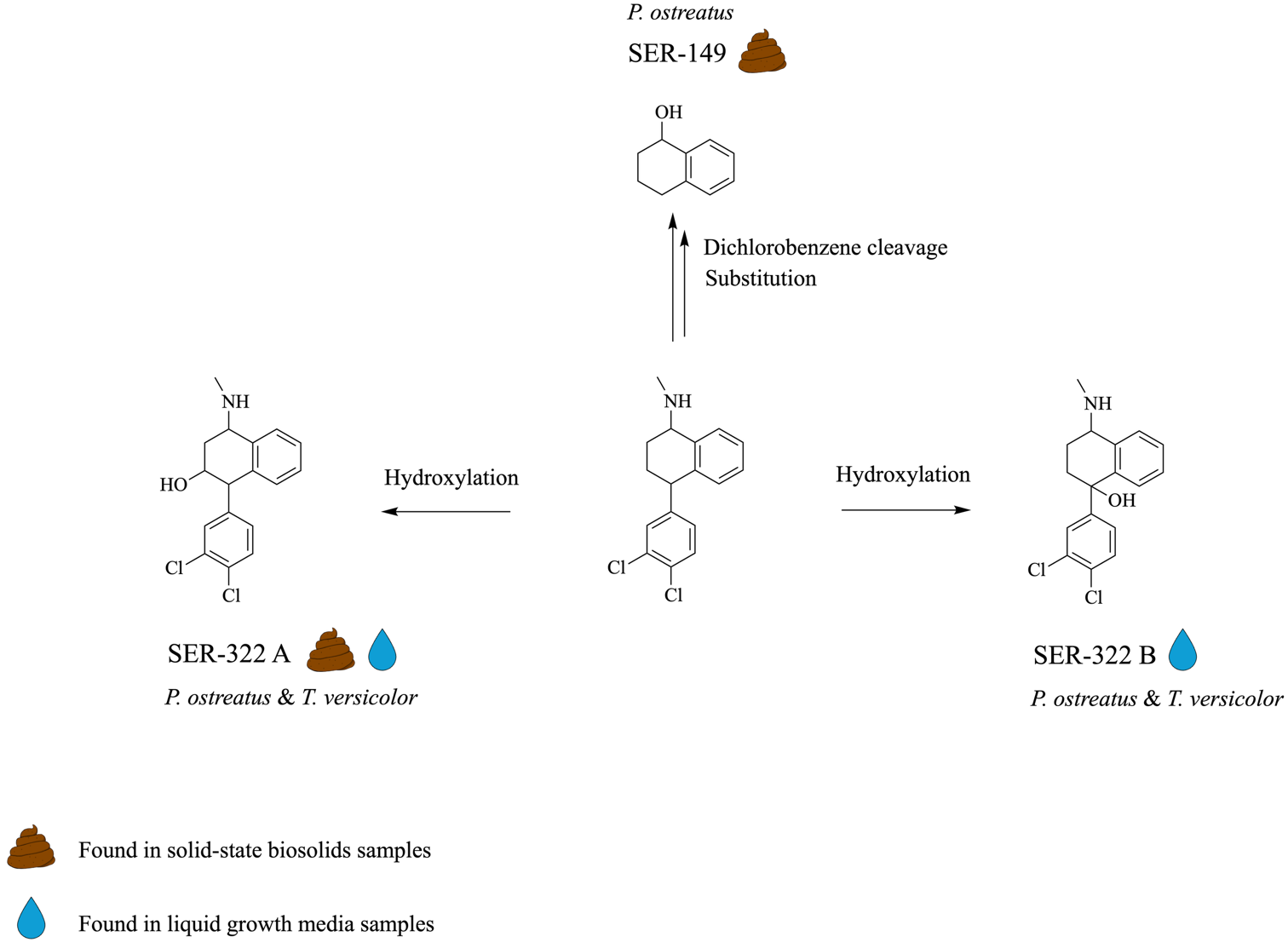


**Figure S11. Proposed transformation pathway of sertraline (SER) by white-rot fungi *P. ostreatus* and/or *T. versicolor*.** Metabolites SER-322 A and B were predicted metabolites by Biotransformer 3.0. A metabolite of sertraline by *P. ostreatus* with the same mass as SER-322A/B was reported by Kózka et al. though they proposed a structure with an oxidation on the benzene ring. SER-149 has been reported as a sertraline byproduct of *P. chrysosporium* degradation by Kózka et al.

**Table S12. Normalized peak areas of sertraline metabolites overs time.** Ser-149 was only found in solid-state *P. ostreatus* samples but SER-322A/B increased over time in all sample matrices.

| **TP** | ***P. ostreatus* liquid** | ***T. versicolor* liquid** | ***P. ostreatus* and/or *T. versicolor* solid** |
| --- | --- | --- | --- |
| SER-149 |  |  |  |
| SER-322A |  |  |  |
| SER-322B |  |  |  |


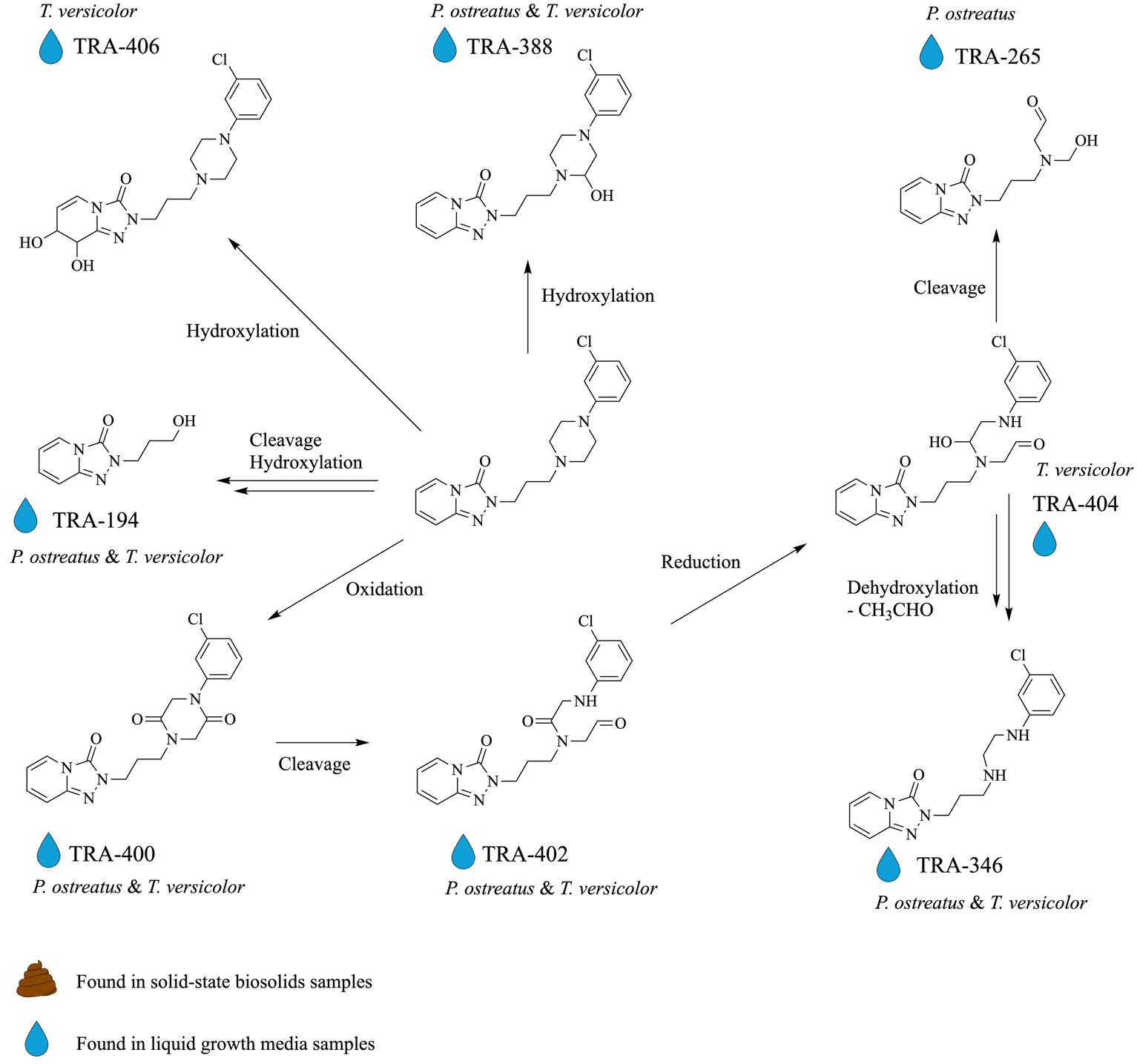


**Figure S12. Proposed transformation pathway of trazodone (TRA) by white-rot fungi *P. ostreatus* and/or *T. versicolor*.** Metabolites TRA-346, TRA-388, and TRA-404 were predicted trazodone metabolites by Biotransformer 3.0. The rest of metabolites have never been reported.

**Table S13. Normalized peak areas of trazodone metabolites overs time.** No metabolites were identified in solid-state samples.

| **TP** | ***P. ostreatus* liquid** | ***T. versicolor* liquid** |
| --- | --- | --- |
| TRA-194 |  |  |
| TRA-265 |  |  |
| TRA-346 |  |  |
| TRA-388 |  |  |
| TRA-400 |  |  |
| TRA-402 |  |  |
| TRA-404 |  |  |
| TRA-406 |  |  |


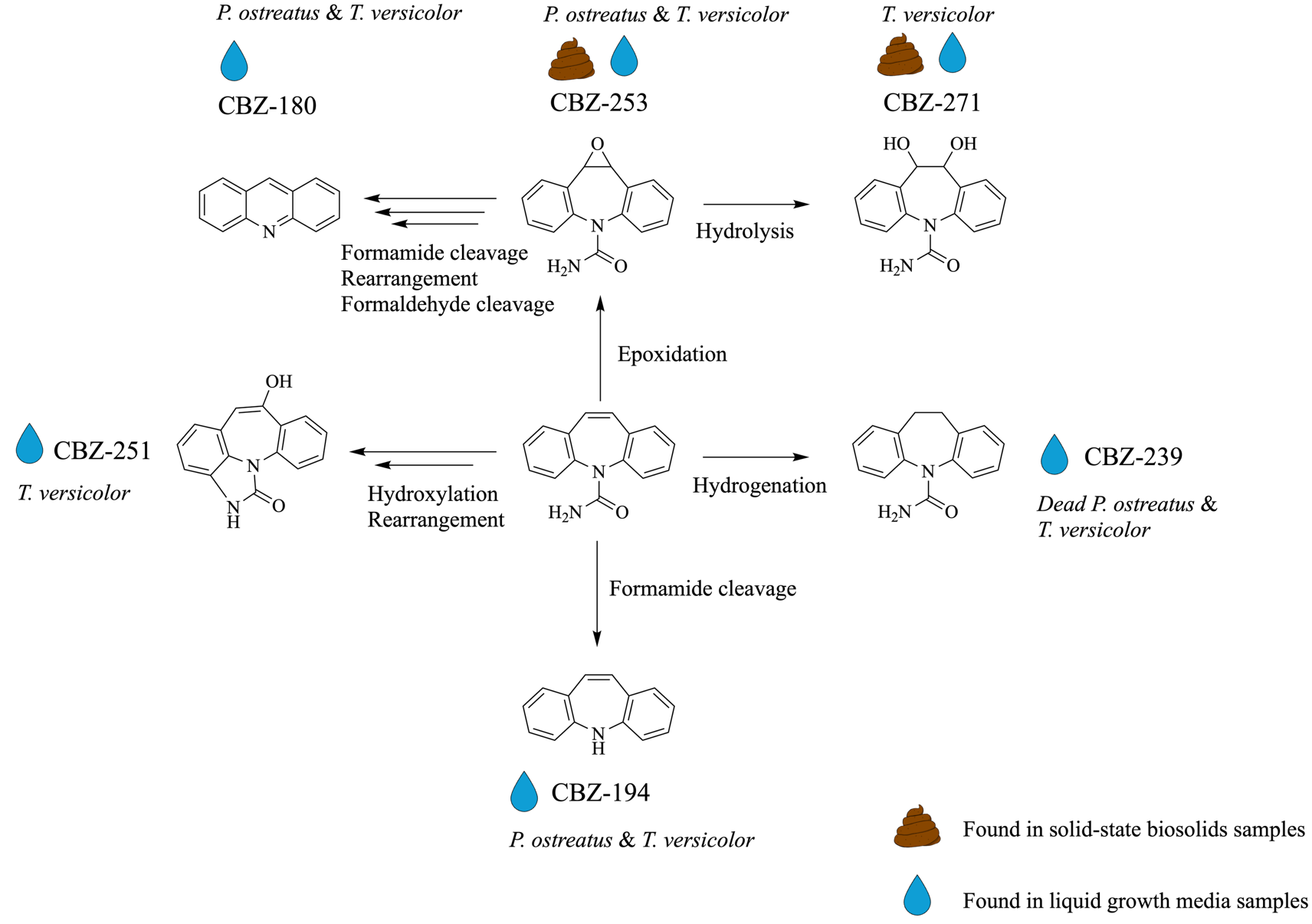


**Figure S13. Proposed transformation pathway of carbamazepine (CBZ) by white-rot fungi *P. ostreatus* and/or *T. versicolor*.** Metabolites CBZ-180, CBZ-194, CBZ-253, and CBZ-239 were all matched to known spectra in Thermo’s mzcloud library.

**Table S14. Normalized peak areas of carbamazepine metabolites overs time.**

| **TP** | ***P. ostreatus* liquid** | ***T. versicolor* liquid** | ***P. ostreatus* and/or *T. versicolor* solid** |
| --- | --- | --- | --- |
| CBZ-180 |  |  |  |
| CBZ-194 |  |  |  |
| CBZ-239 |  |  |  |
| CBZ-251 |  |  |  |
| CBZ-253 |  |  |  |
| CBZ-271 |  |  |  |


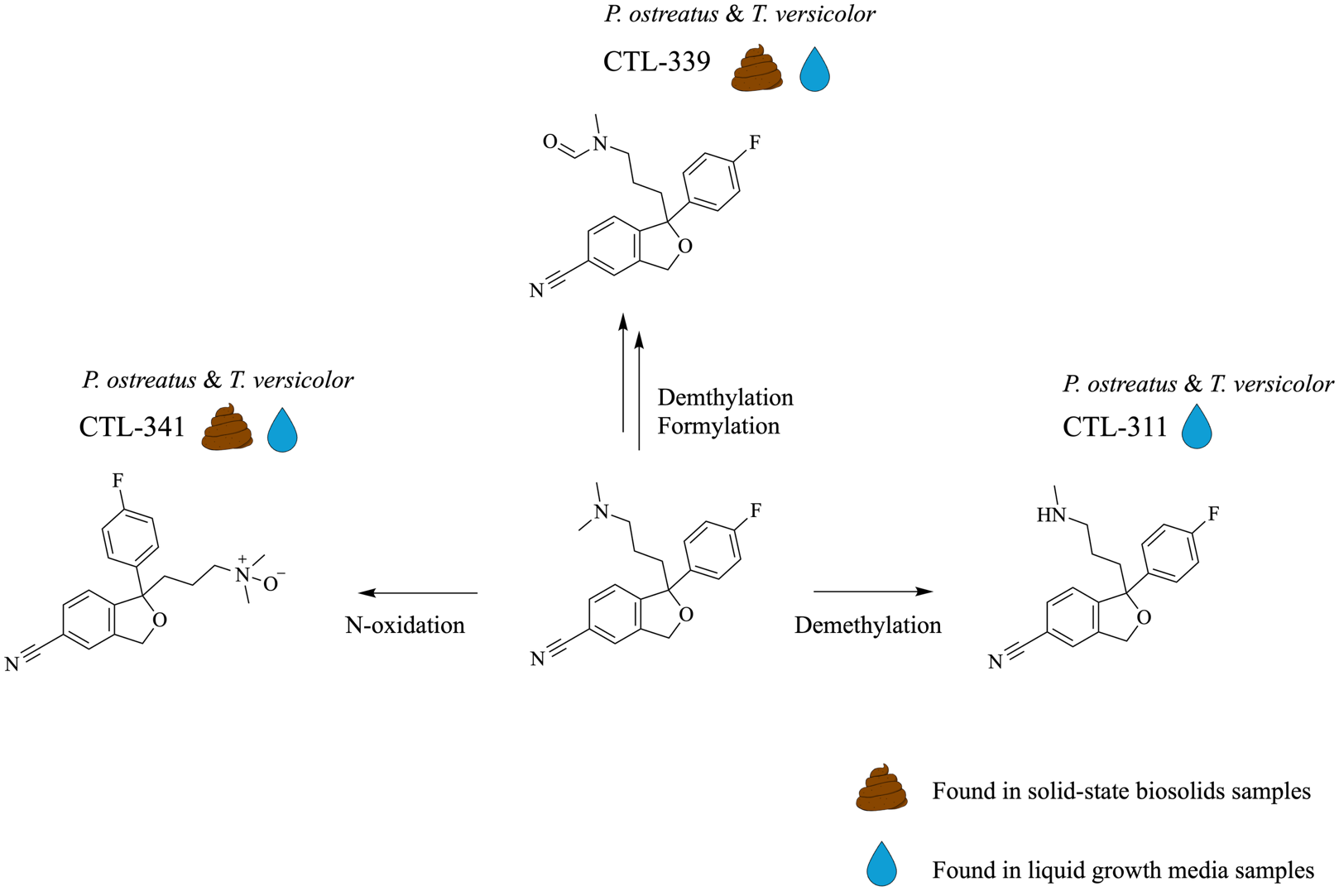


**Figure S14. Proposed transformation pathway of citalopram (CTL) by white-rot fungi *P. ostreatus* and *T. versicolor*.** Fragmentation from metabolite CTL-311 (desmethylcitalopram) matched to known spectra in Thermo’s mzCloud library.

**Table S15. Normalized peak areas of citalopram metabolites overs time.**

| **TP** | ***P. ostreatus* liquid** | ***T. versicolor* liquid** | ***P. ostreatus* and/or *T. versicolor* solid** |
| --- | --- | --- | --- |
| CTL-311 |  |  |  |
| CTL-339 |  |  |  |
| CTL-341 |  |  |  |


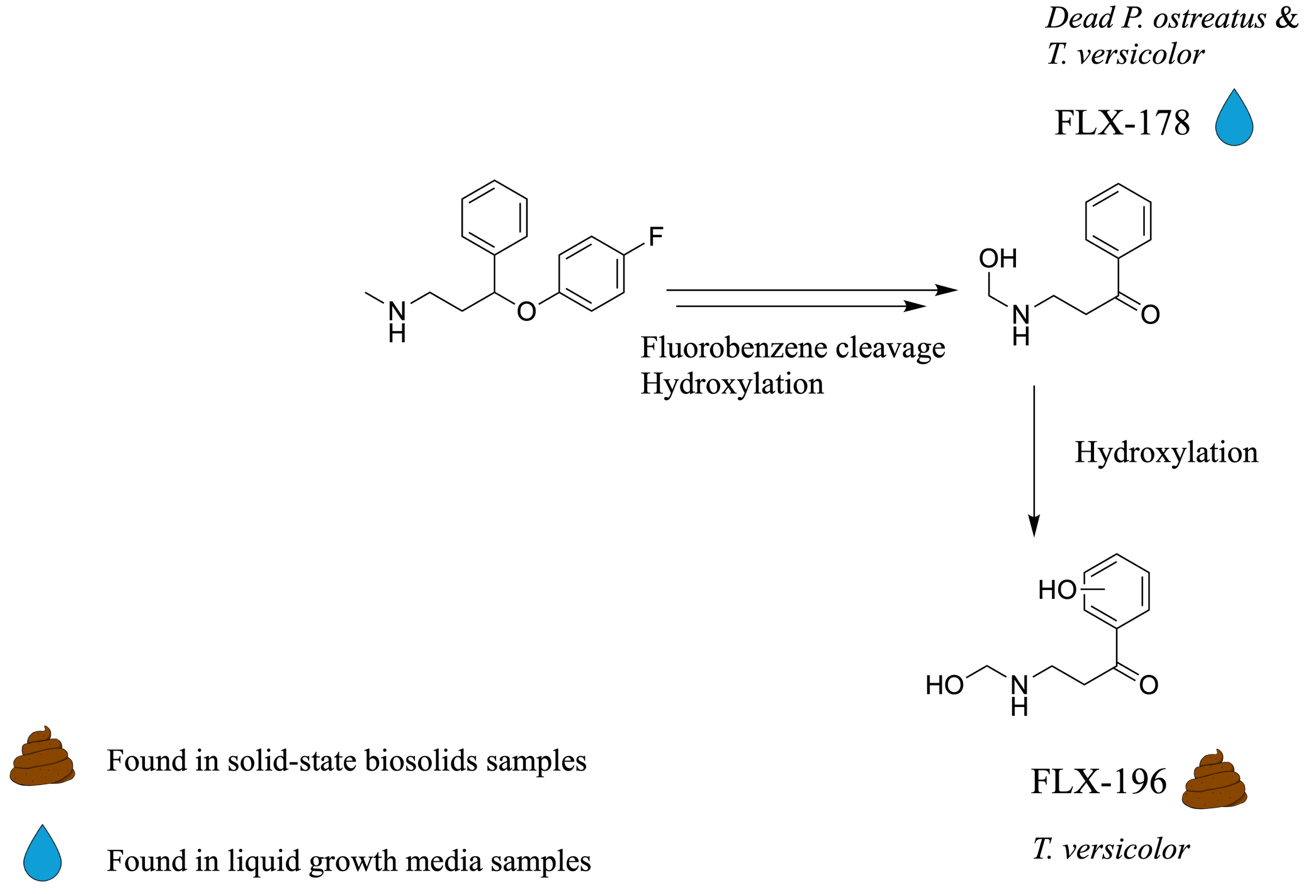


**Figure S15. Proposed transformation pathway of fluoxetine (FLX) by white-rot fungi *P. ostreatus* and/or *T. versicolor*.**

**Table S16. Normalized peak areas of fluoxetine metabolites over time.**

| **TP** | ***P. ostreatus* liquid** | ***T. versicolor* liquid** | ***P. ostreatus* and/or *T. versicolor* solid** |
| --- | --- | --- | --- |
| FLX-178 |  |  |  |
| FLX-196 |  |  |  |

**Table S17. Hazard screening of parent and metabolites.** Compounds were assessed using EPA′s Cheminformatics Hazard Comparison Module (HCM, <https://www.epa.gov/comptox-tools/cheminformatics>). Human health effects, ecotoxicity, and environmental fate end points were predicted and a letter score was given for each end point: very high (VH), high (H), medium (M), low (L), and I (inconclusive). Bolded values indicate scores were assigned based on authoritative measures of toxicity from recognized experts. Scores reported in standard font were assigned via screening data, usually from toxicity reports that have been estimated by an organization not considered authoritative. Finally, scores italicized are predicted scores from QSAR models and are therefore ^[[1]](#footnote-1)^the least authoriative.^17^ Many of these metabolites are not well-studied or novel, so it is not surprising that the majority of their hazard scores are based on *in silico* QSAR modeling. Cheminformatics could not generate any information on vilazodone which is why there are no scores.

| **Metabolite** | **Human Health Effects** | | | | | | **Ecotoxicity** | | | **Fate** | **Average Hazard Score** | **Average Quality Score** | **Quality-adjusted Hazard Score** | **Completeness Score** |
| --- | --- | --- | --- | --- | --- | --- | --- | --- | --- | --- | --- | --- | --- | --- |
|  | Acute Mammalian Toxicity: Oral | Carcinogenicity | Genotoxicity Mutagenicity | Endocrine Disruption | Reproductive | Developmental toxicity | Acute aquatic toxicity | Chronic Aquatic toxicity | Persistence | Bioaccumulation |  |  |  |  |
| **Amitriptyline** | H |  | L | *H* | *M* | H | VH | *VH* |  | H | 2.88 | 1.63 | 4.68 | 0.8 |
| AMI-264 | M |  | H | *L* | *M* | H | H | *VH* |  | H | 2.63 | 1.63 | 4.27 | 0.8 |
| AMI-276 | H |  |  |  |  |  |  |  |  |  | 3.00 | 2.00 | 6.00 | 0.1 |
| AMI-280 | *M* |  | *L* | *H* |  | *H* | *VH* |  |  | *L* | 2.33 | 1.00 | 2.33 | 0.6 |
| AMI-292 | *I* |  | *L* |  |  | *H* | *VH* |  |  | *L* | 2.25 | 1.00 | 2.25 | 0.4 |
| AMI-294 | *H* |  | *L* |  |  | *H* | *VH* |  |  | *L* | 2.40 | 1.00 | 2.40 | 0.5 |
| AMI-310A | *M* |  | *L* |  |  | *H* | *I* |  |  | *I* | 2.00 | 1.00 | 2.00 | 0.3 |
| AMI-310B | *M* |  | *L* |  |  | *H* | *I* |  |  | *I* | 2.00 | 1.00 | 2.00 | 0.3 |
| AMI-312 | *M* |  | *L* |  |  | *H* | *I* |  |  | *I* | 2.00 | 1.00 | 2.00 | 0.3 |
| **Vilazodone** | *I* |  |  | *L* |  | *I* | *I* |  |  | *I* | 1.00 | 1.00 | 1.00 | 0.1 |
| VIL-229 | *I* |  | *H* |  |  | *H* | *H* |  |  | *I* | 4.00 | 1.00 | 4.00 | 0.3 |
| **Sertraline** | *M* |  | *L* | *H* |  | *H* | **VH** | **VH** |  | *M* | 2.71 | 1.57 | 4.26 | 0.7 |
| SER-148 | M |  | *L* |  |  | *H* | *M* |  |  | *L* | 1.80 | 1.20 | 2.16 | 0.5 |
| SER-322A | *M* |  | *L* |  |  | *H* | *VH* |  |  | *M* | 2.40 | 1.00 | 2.40 | 0.5 |
| SER-322B | *M* |  | *L* |  |  | *H* | *H* |  |  | *M* | 2.20 | 1.00 | 2.20 | 0.5 |
| **Desvenlafaxine** | *M* |  | *L* | *H* |  | *H* | *H* |  |  | *L* | 2.67 | 1.00 | 2.67 | 0.6 |
| DVEN-115 | *L* |  | *L* | *L* |  | *H* | *L* | *M* |  | *L* | 1.43 | 1.00 | 1.43 | 0.7 |
| DVEN-129 | *M* |  | *L* | *L* |  | *H* | *L* |  |  | *L* | 1.50 | 1.00 | 1.50 | 0.6 |
| DVEN-232 | *M* |  | *H* |  |  | *H* | *H* |  |  | *L* | 2.40 | 1.00 | 2.40 | 0.5 |
| DVEN-234 | *M* |  | *L* |  |  | *H* | *H* |  |  | *L* | 2.00 | 1.00 | 2.00 | 0.5 |
| DVEN-237 | *M* |  | *L* |  |  | *H* | *M* |  |  | *L* | 1.80 | 1.00 | 1.80 | 0.5 |
| DVEN-250 | *M* |  | *L* | *H* |  | *H* | *H* |  |  | *L* | 2.17 | 1.00 | 2.17 | 0.6 |
| DVEN-251 | *M* |  | *L* |  |  | *H* | *H* |  |  | *L* | 2.00 | 1.00 | 2.00 | 0.5 |
| DVEN-280 | *M* |  | *L* |  |  | *H* | *M* |  |  | *L* | 1.80 | 1.00 | 1.80 | 0.5 |
| DVEN-308 | *M* |  | *L* |  |  | *H* | *M* |  |  | *L* | 1.80 | 1.00 | 1.80 | 0.5 |
| DVEN-336 | *I* |  | *L* |  |  | *H* | *H* |  |  | *I* | 2.33 | 1.00 | 2.33 | 0.3 |
| **Trazodone** | M |  | *H* | *H* |  | *H* | *M* |  |  | *L* | 2.33 | 1.17 | 2.72 | 0.6 |
| TRA-194 | *I* |  | *H* |  |  | *H* | *L* |  |  | *L* | 2.00 | 1.00 | 2.00 | 0.4 |
| TRA-265 | *I* |  | *H* |  |  | *H* | *L* |  |  | *I* | 2.33 | 1.00 | 2.33 | 0.3 |
| TRA-346 | *M* |  | *L* |  |  | *H* | *M* |  |  | *L* | 1.80 | 1.00 | 1.80 | 0.5 |
| TRA-388 | *M* |  | *H* |  |  | *H* | *M* |  |  | *L* | 2.20 | 1.00 | 2.20 | 0.5 |
| TRA-400 | *M* |  | *H* |  |  | *H* | *M* |  |  | *L* | 2.20 | 1.00 | 2.20 | 0.5 |
| TRA-402 | *I* |  | *H* |  |  | *H* | *L* |  |  | *I* | 2.33 | 1.00 | 2.33 | 0.3 |
| TRA-404 | *I* |  | *H* |  |  | *H* | *M* |  |  | *I* | 2.67 | 1.00 | 2.67 | 0.3 |
| TRA-406 | *M* |  | *H* |  |  | *H* | *L* |  |  | *L* | 2.00 | 1.00 | 2.00 | 0.5 |
| **Carbamazepine** | M | *H* | *H* | *L* |  | **H** | **M** | **VH** |  | L | 2.38 | 2.00 | 4.76 | 0.8 |
| CBZ-180 | M |  | VH | H |  | *L* | **H** | **H** | *M* | M | 2.50 | 2.00 | 5.00 | 0.8 |
| CBZ-194 | *M* |  | *L* | *L* |  | *H* | *VH* | *VH* |  | *L* | 2.29 | 1.00 | 2.29 | 0.7 |
| CBZ-239 | *M* | *H* | *H* | *L* | *M* | *H* | *H* |  |  | *L* | 2.25 | 1.00 | 2.25 | 0.8 |
| CBZ-251 | *M* |  | *L* |  |  | *H* | *H* |  |  | *L* | 2.00 | 1.00 | 2.00 | 0.5 |
| CBZ-253 | *M* |  | *H* | *L* |  | *H* | *VH* |  |  | *L* | 2.33 | 1.00 | 2.33 | 0.6 |
| CBZ-271 | *L* |  | *L* | *L* |  | *H* | *M* |  |  | *L* | 1.50 | 1.00 | 1.50 | 0.6 |
| **Citalopram** | *M* |  | *L* | *H* |  | *H* | **H** |  |  | *M* | 2.33 | 1.33 | 3.11 | 0.6 |
| CTL-311 | *I* |  | *L* | *L* |  | *H* | *H* |  |  | *M* | 2.00 | 1.00 | 2.00 | 0.5 |
| CTL-339 | *I* |  | *L* |  |  | *H* | *H* |  |  | *M* | 2.25 | 1.00 | 2.25 | 0.4 |
| CTL-341 | *H* |  | *H* | *L* |  | *H* | *I* |  |  | *I* | 2.50 | 1.00 | 2.50 | 0.4 |
| **Fluoxetine** | M |  | *L* | *L* |  | H | **VH** | **VH** |  | *M* | 2.43 | 1.86 | 4.51 | 0.7 |
| FLX-178 | *L* |  | *L* |  |  | *H* | *M* |  |  | *L* | 1.60 | 1.00 | 1.60 | 0.5 |
| FLX-196 | *M* |  | *L* |  |  | *H* | *M* |  |  | *L* | 1.80 | 1.00 | 1.80 | 0.5 |

**Figure S16. FISh coverage of AMI-280 MS^2^ fragmentation.** Fragment with m/z of 178 is a C_14_H_10_ backbone fragment seen in other amitriptyline metabolite MS^2^ (Figure S21).


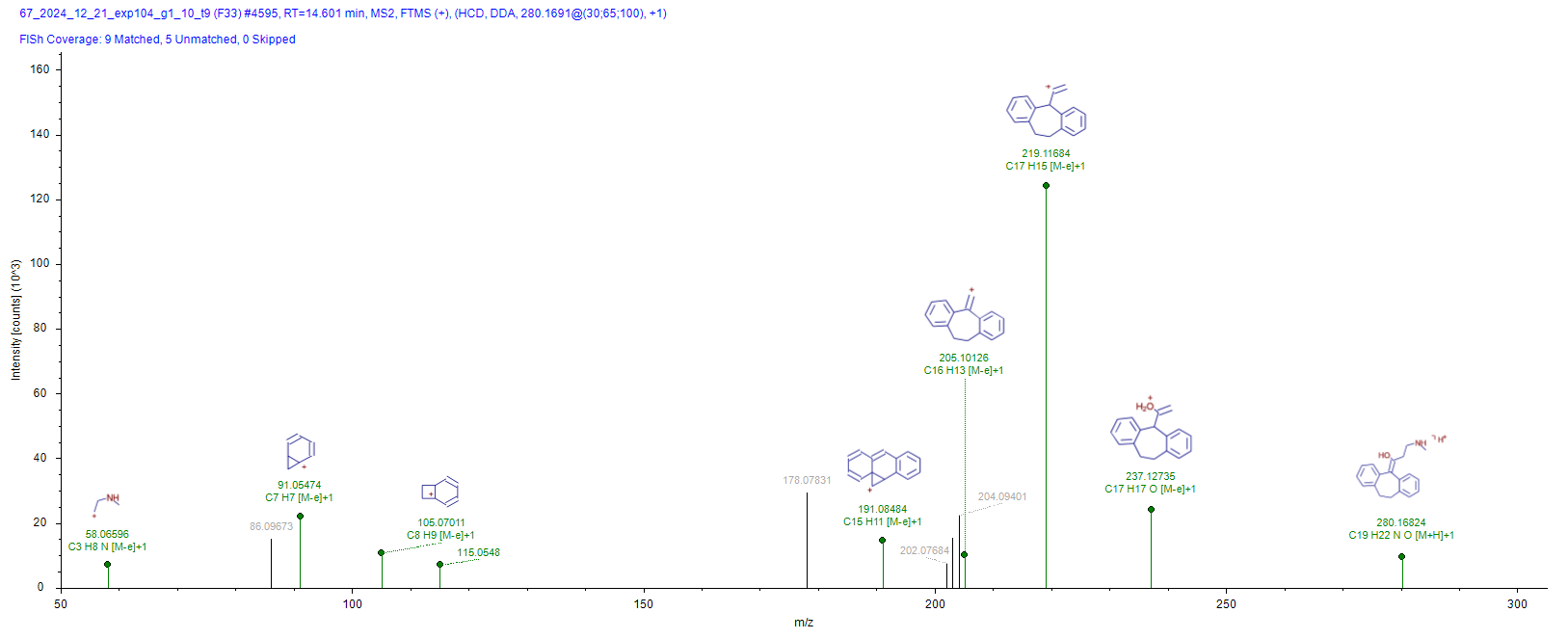


**Figure S17. FISh coverage of AMI-292 MS^2^ fragmentation.**


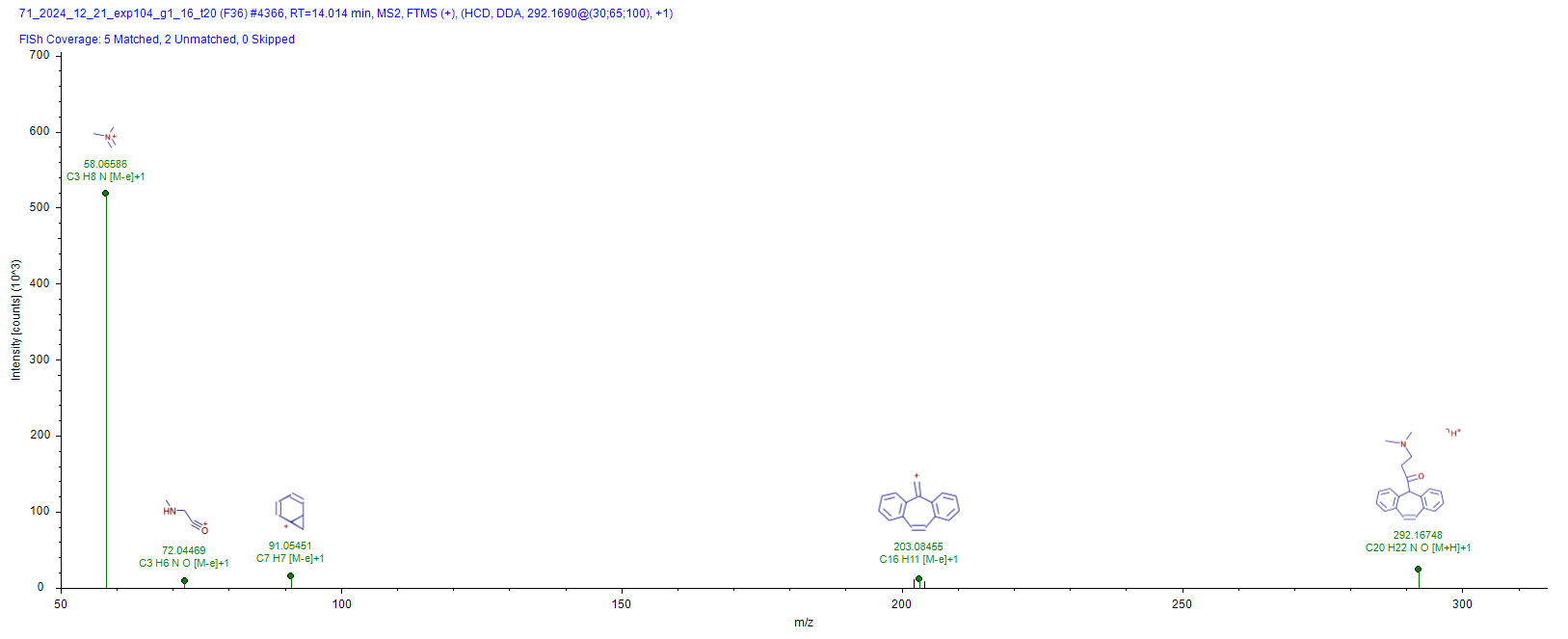

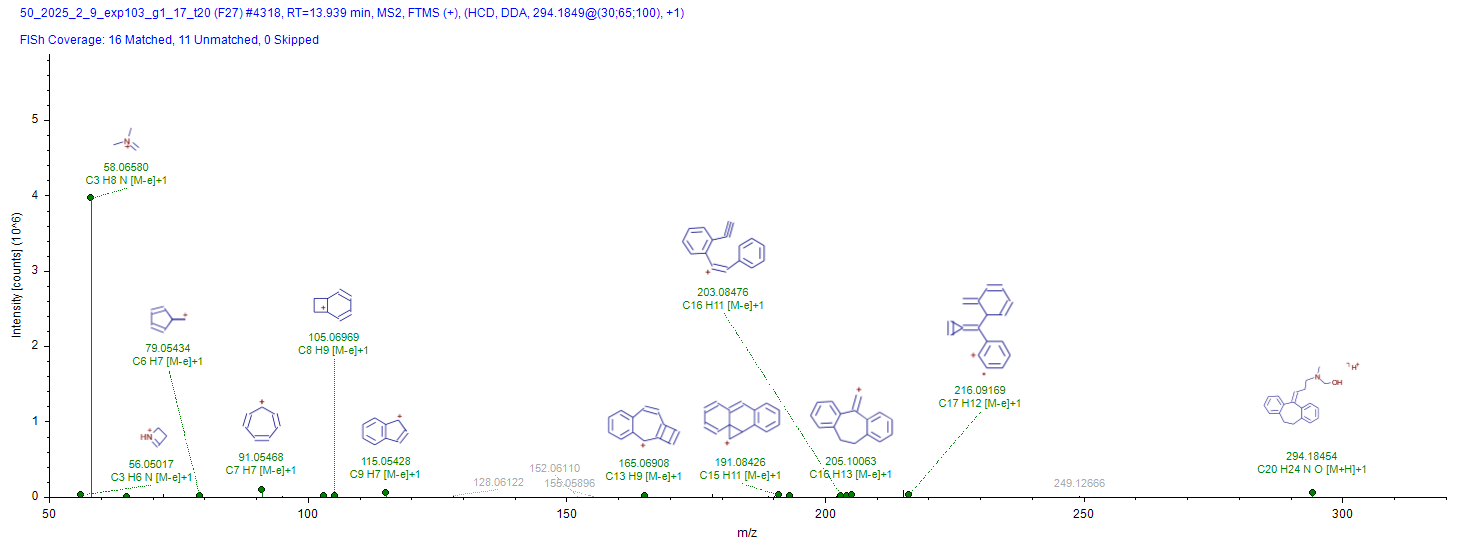


**Figure S18. FISh coverage of AMI-294 MS^2^ fragmentation.**


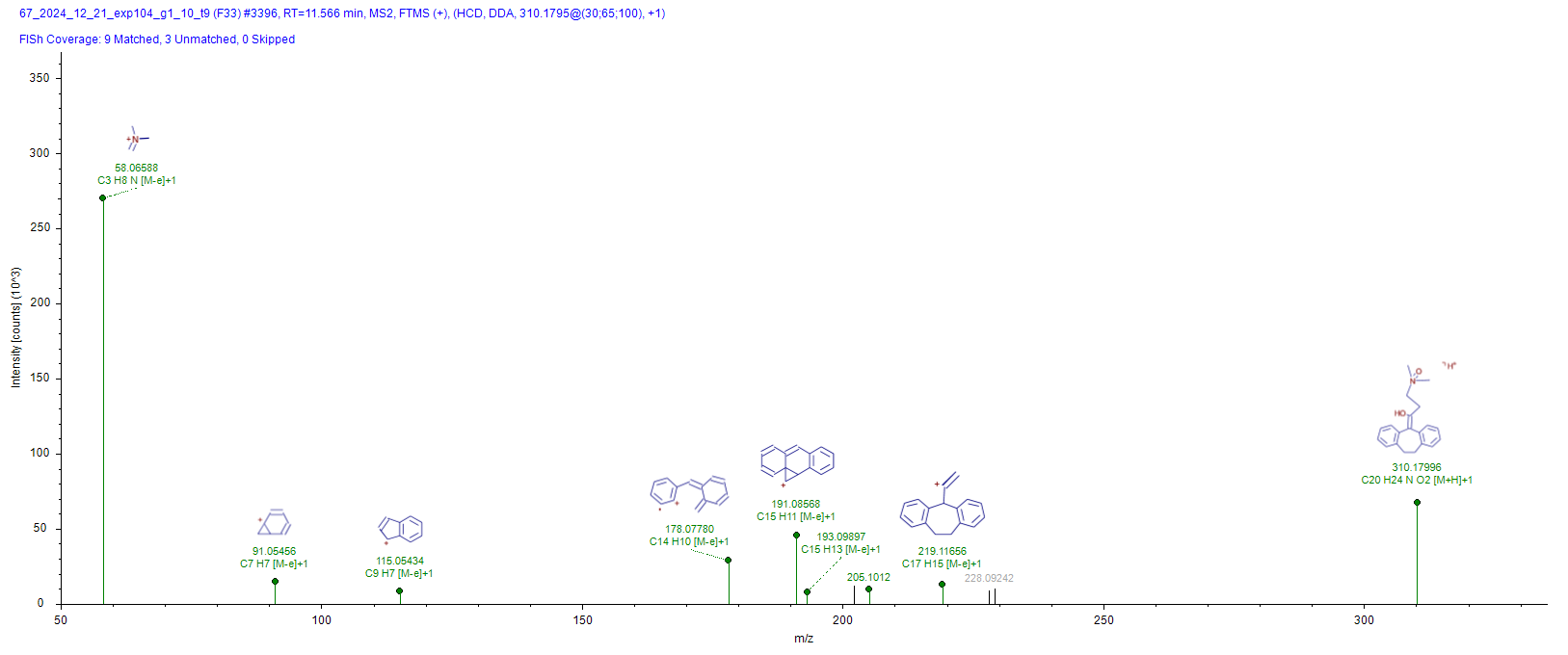


**Figure S19. FISh coverage of AMI-310A MS^2^ fragmentation.**


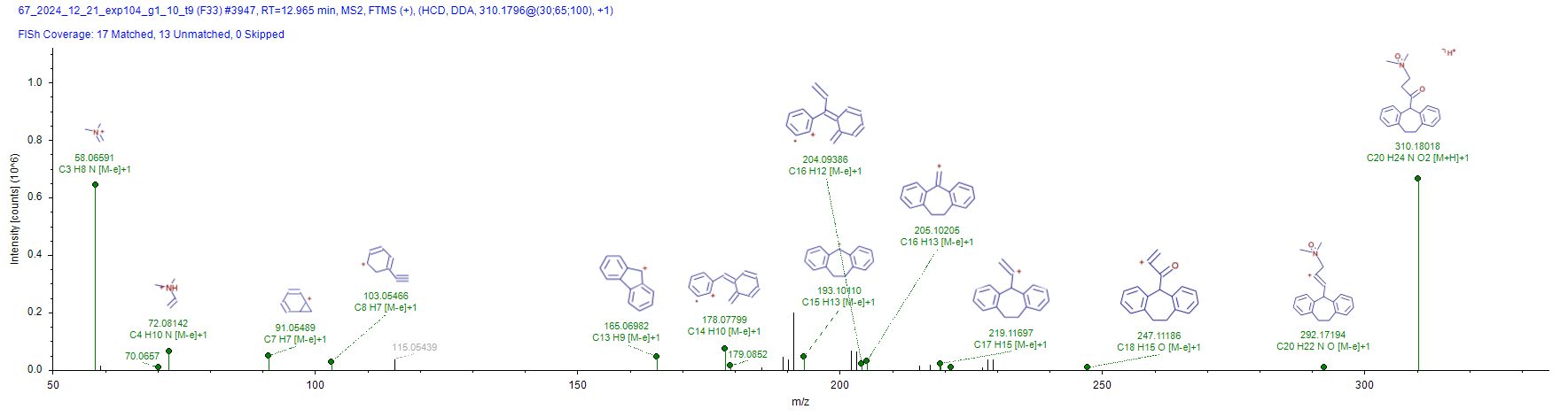


**Figure S20. FISh coverage of AMI-310B MS^2^ fragmentation**.


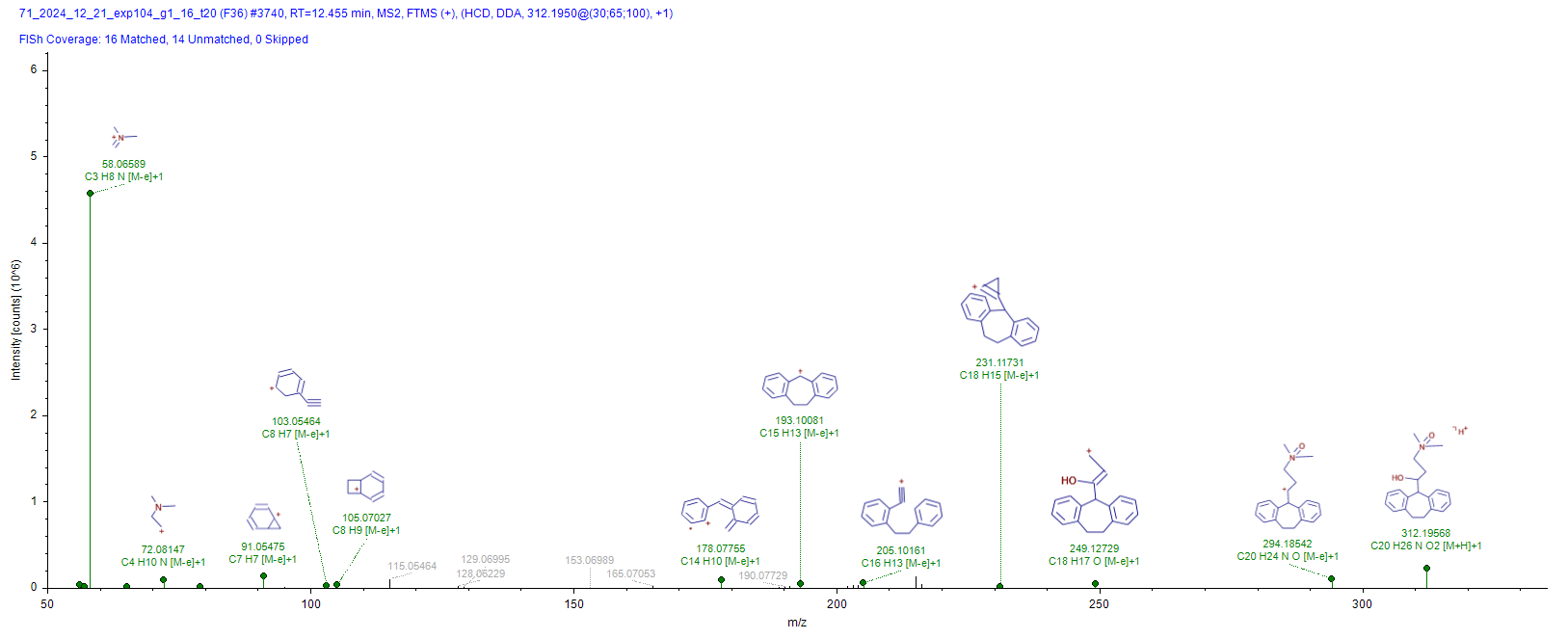


**Figure S21. FISh coverage of AMI-312 MS^2^ fragmentation.**


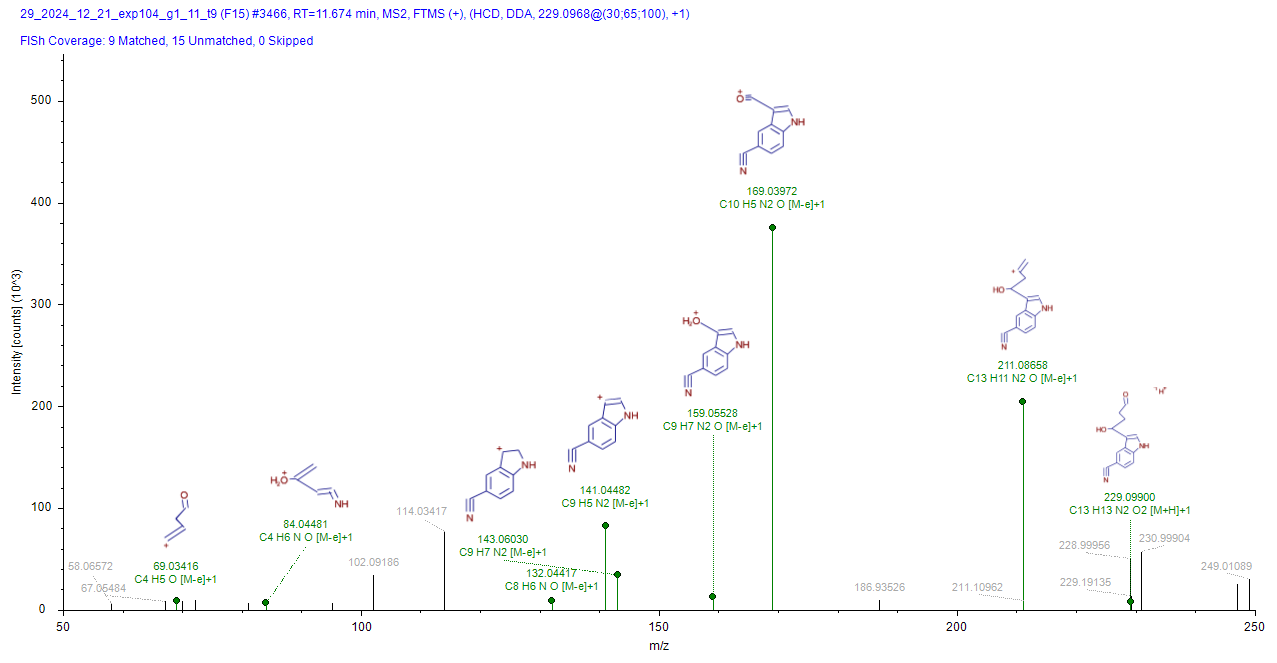


**Figure S22. FISh coverage of VIL-229 MS^2^ fragmentation.** The 114.0341 m/z fragment corresponds to a benzonitrile ion (C_8_H_4_N).


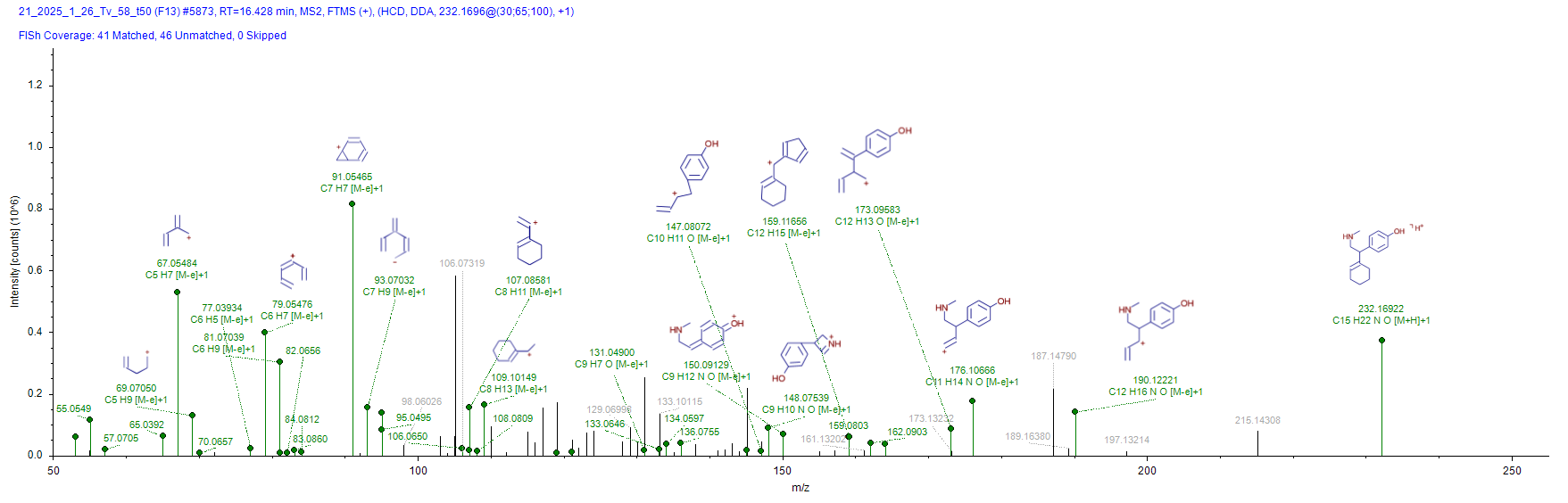


**Figure S23. FISh coverage of DVEN-232 MS^2^ fragmentation.**


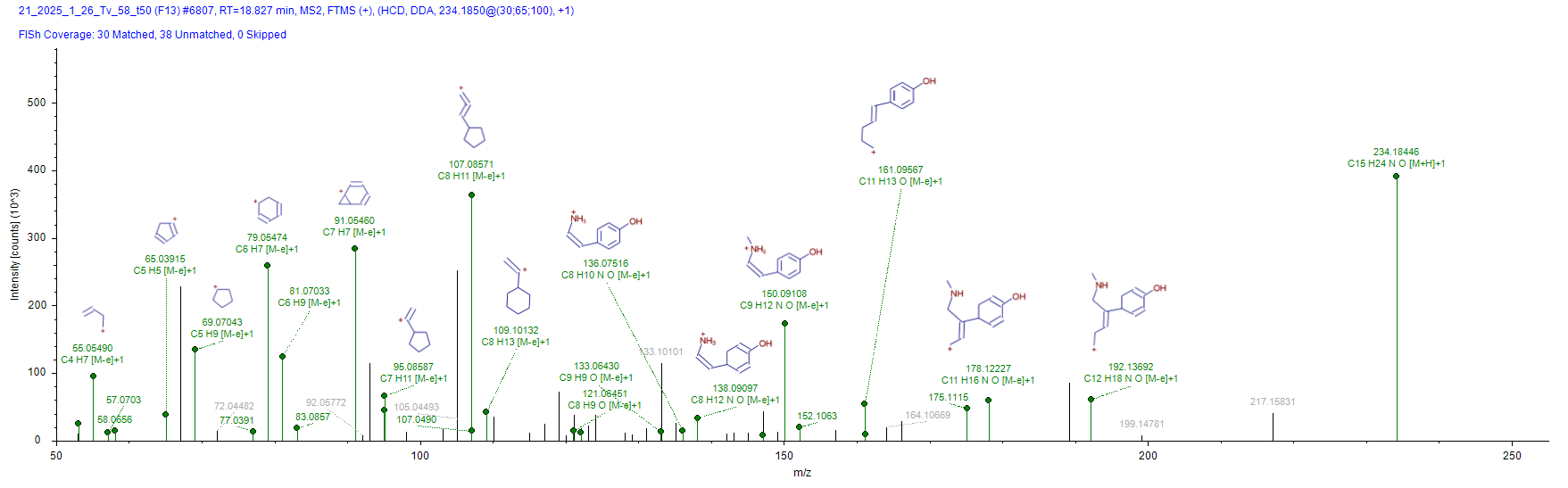


**Figure S24. FISh coverage of DVEN-234 MS^2^ fragmentation.**


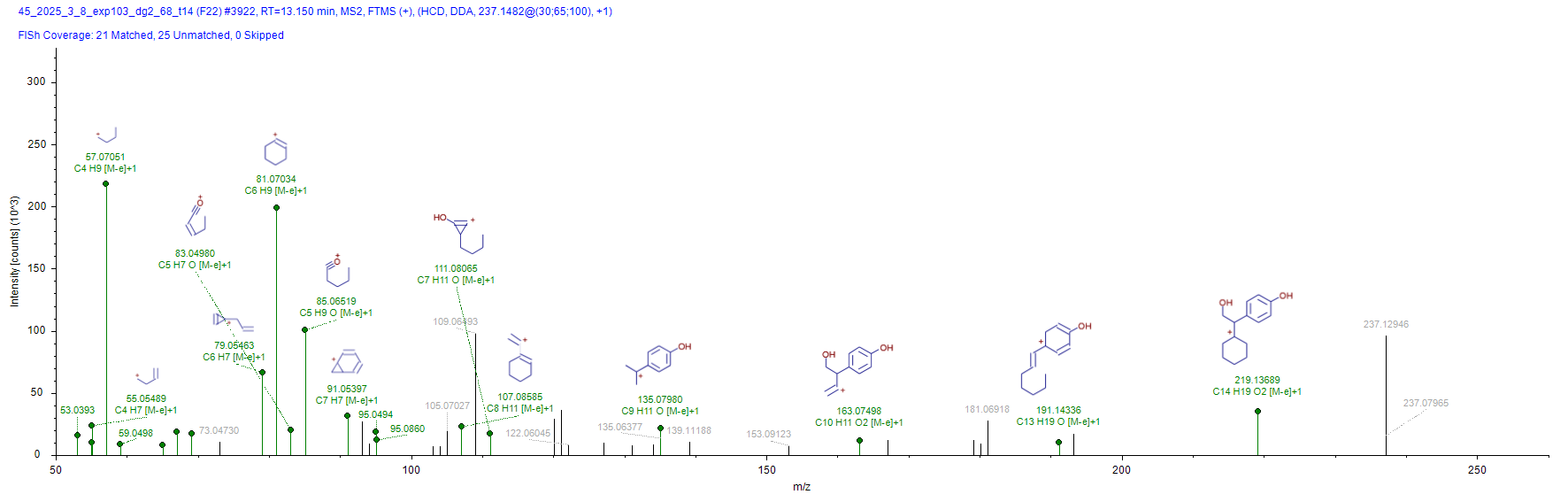


**Figure S25. FISh coverage of DVEN-236 MS^2^ fragmentation.**


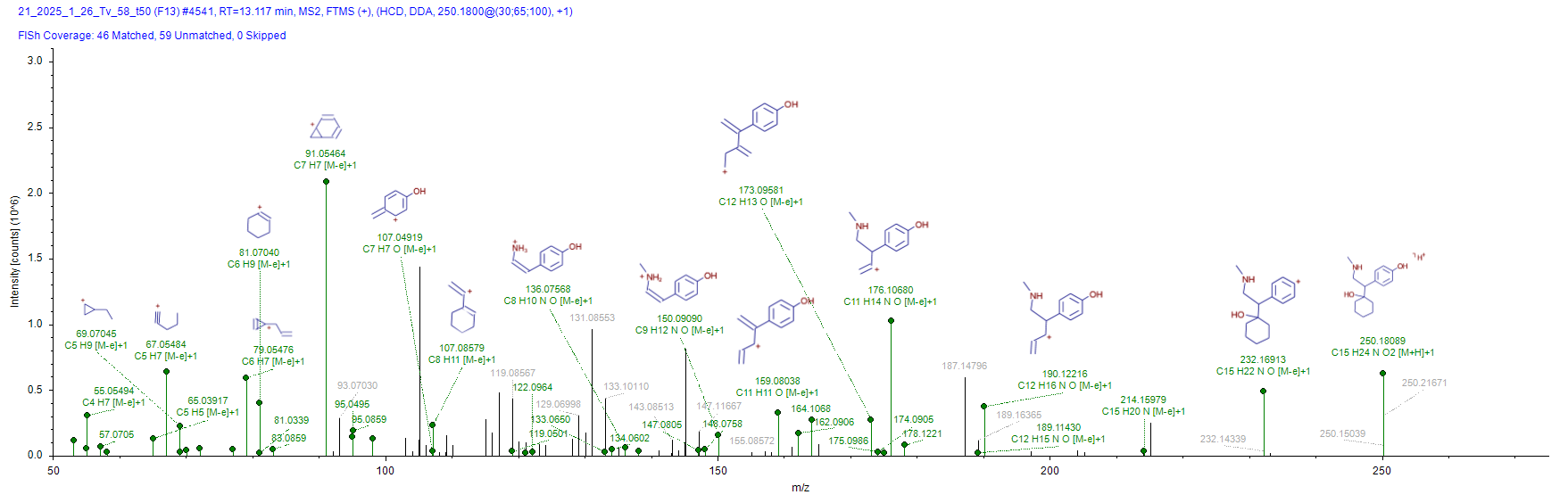


**Figure S26. FISh coverage of DVEN-250 MS^2^ fragmentation.**


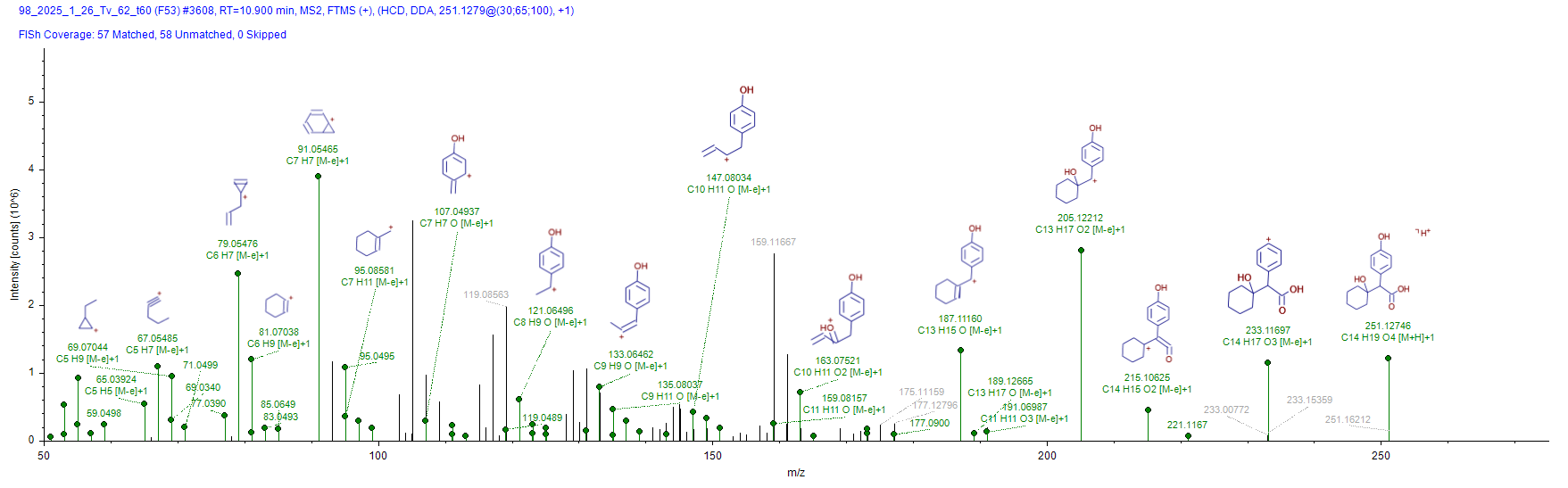


**Figure S27. FISh coverage of DVEN-251 MS^2^ fragmentation.**


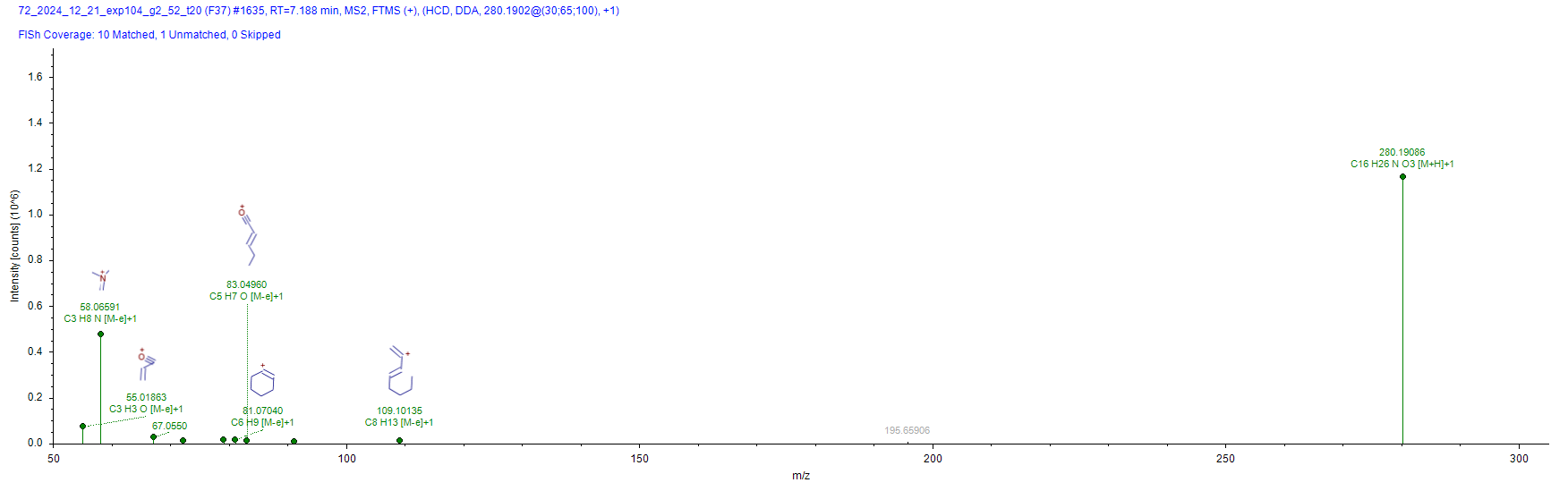


**Figure S28. FISh coverage of DVEN-280 MS^2^ fragmentation.**


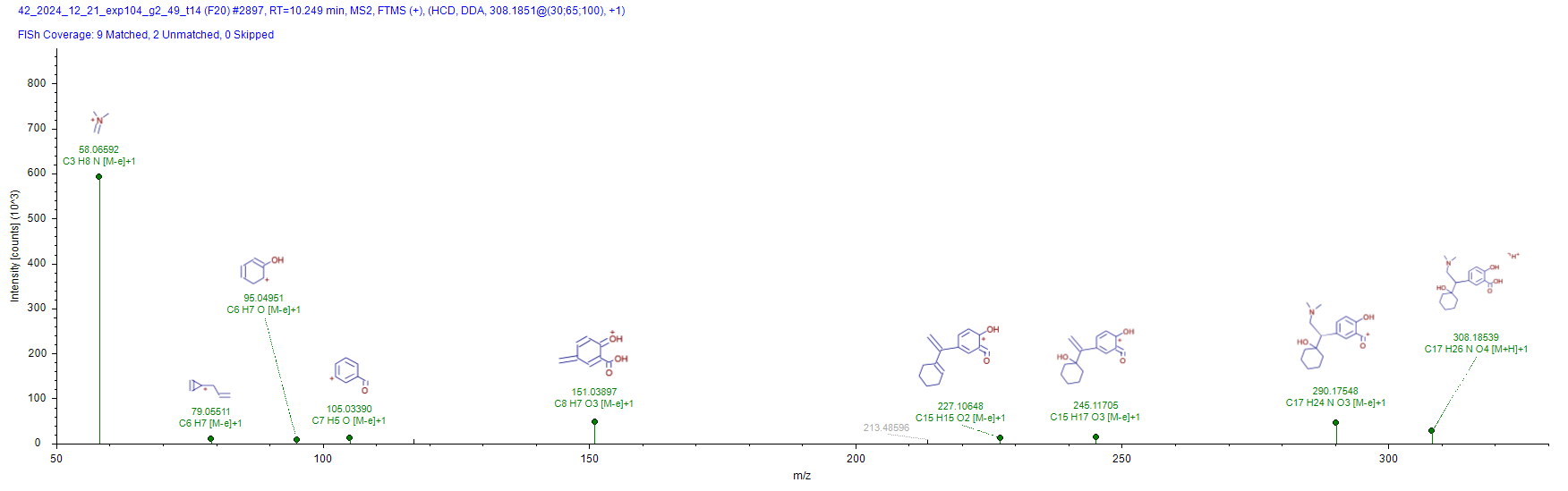


**Figure S29. FISh coverage of DVEN-308 MS^2^ fragmentation.**


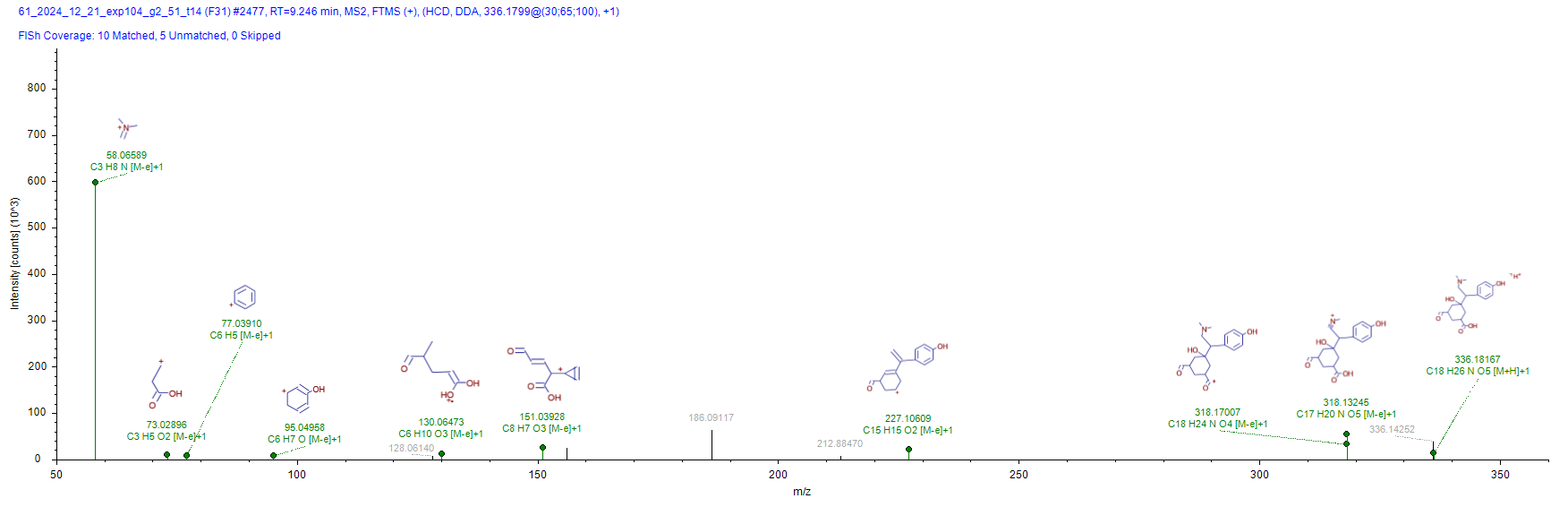


**Figure S30. FISh coverage of DVEN-336 MS^2^ fragmentation.**


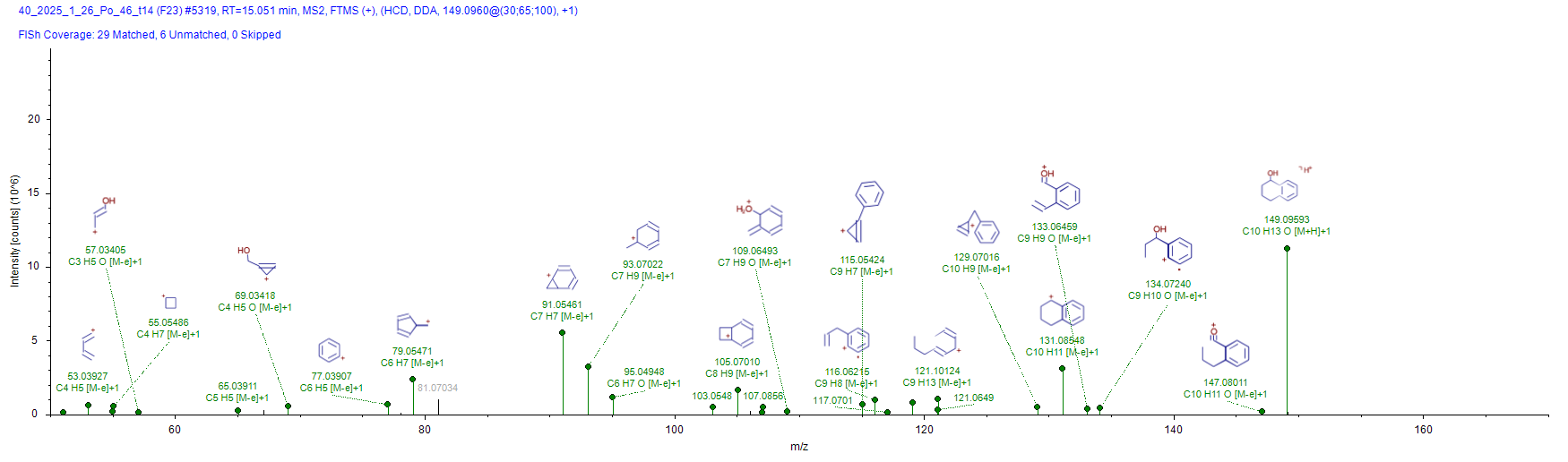


**Figure S31. FISh coverage of SER-148 MS^2^ fragmentation.**


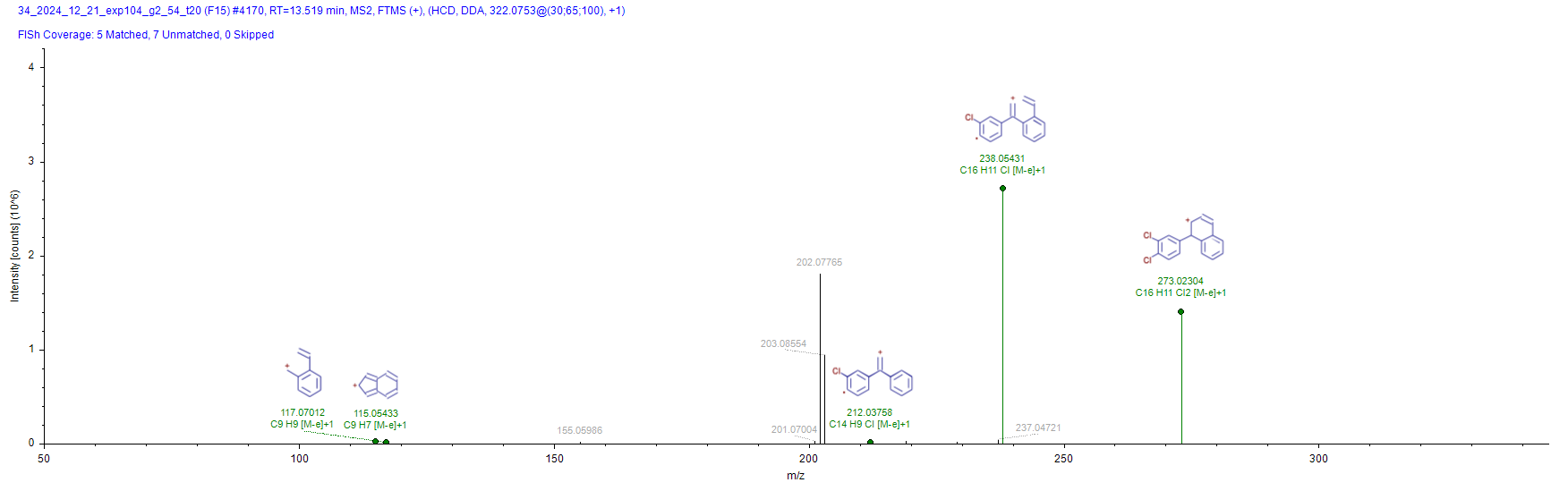


**Figure S32. FISh coverage of SER-321A MS^2^ fragmentation.** The 202.077 and 203.085 m/z fragments are C_16_H_10_ and C_16_H_11_ ions which correspond to sertraline’s ring backbone without chlorine or the amine group.

x


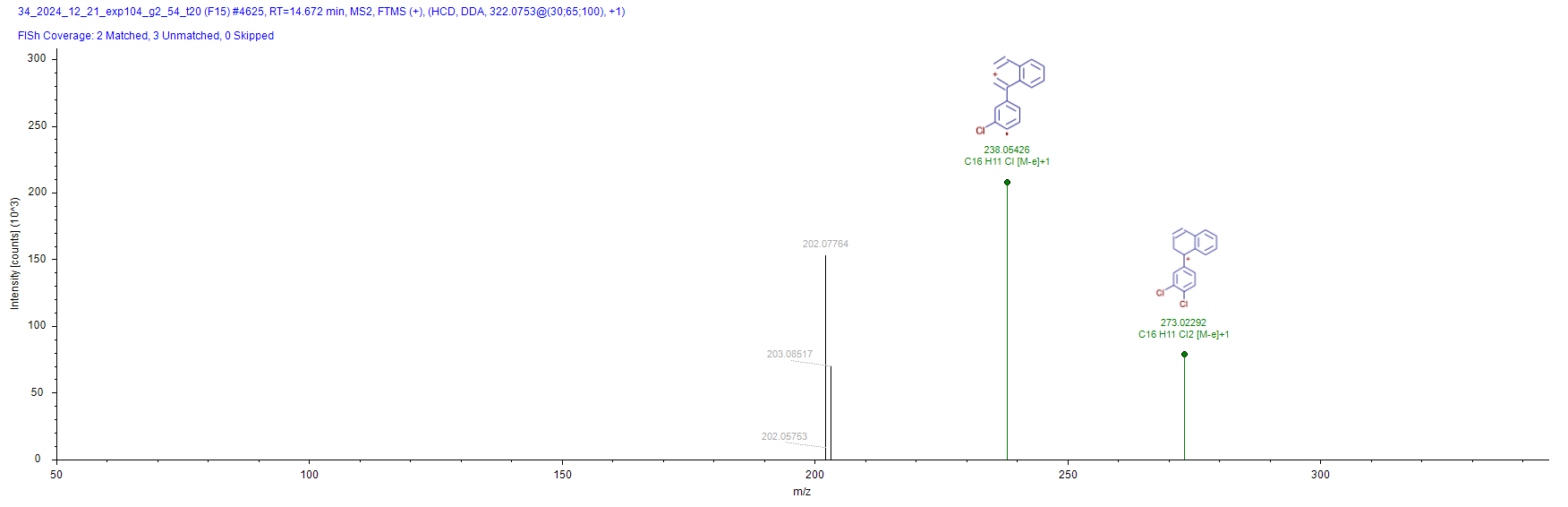


**Figure S33. FISh coverage of SER-321B MS^2^ fragmentation.** See Figure S34 for details on 202 and 203 m/z fragments.


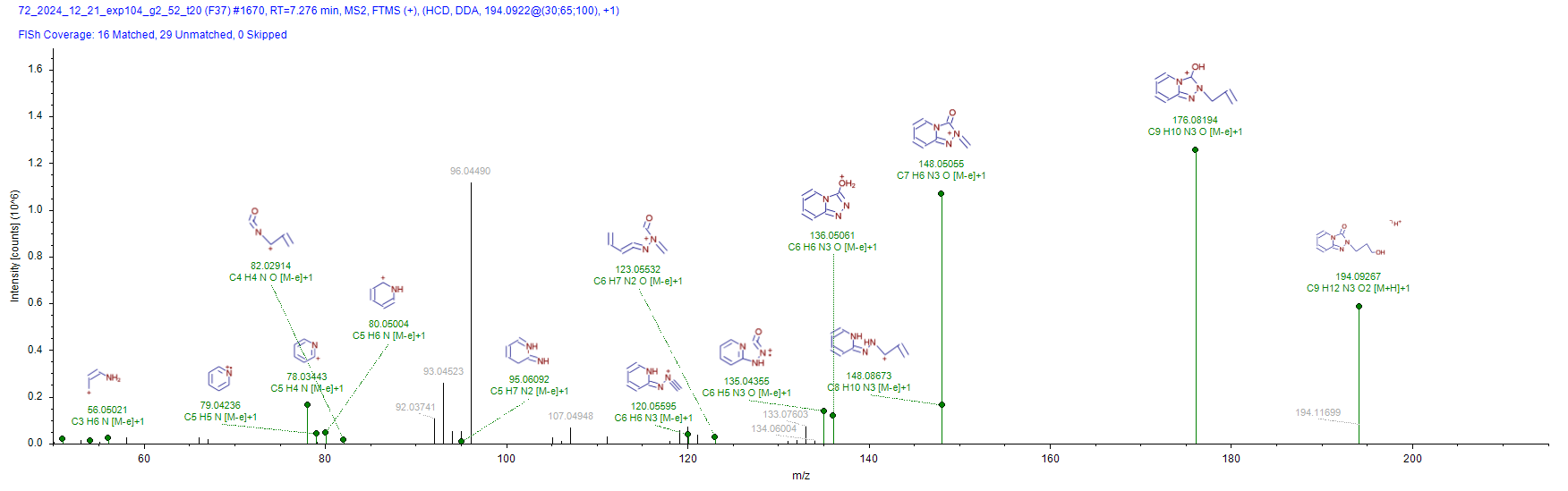


**Figure S34. FISh coverage of TRA-194 MS^2^ fragmentation.** The prominent 96.0449 fragment is a C_5_H_6_NO ion that is present in other trazodone metabolite MS2 (see Figure S37).


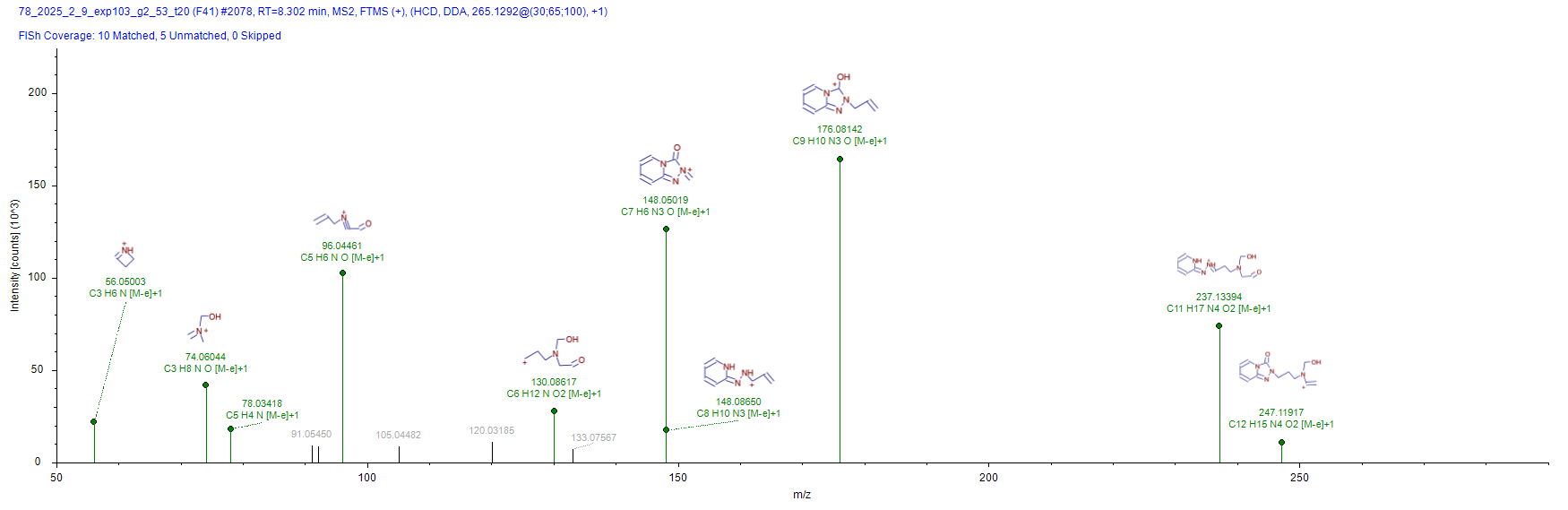


**Figure S35. FISh coverage of TRA-265 MS^2^ fragmentation.**


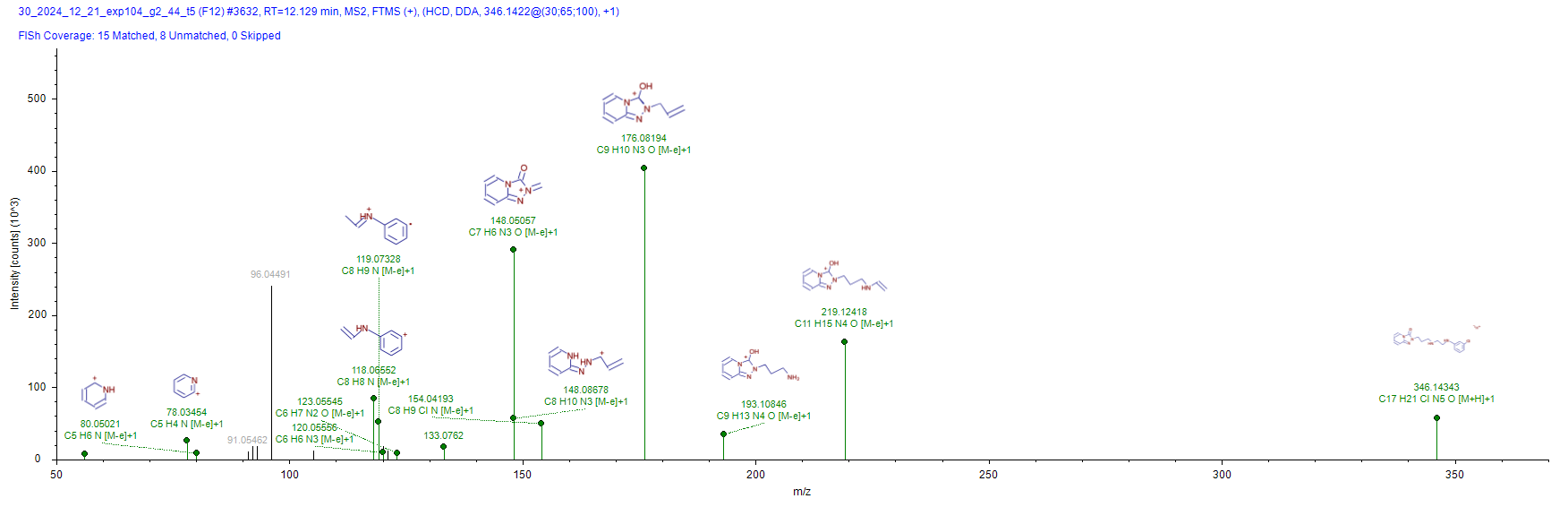


**Figure S36. FISh coverage of TRA-346 MS^2^ fragmentation.**


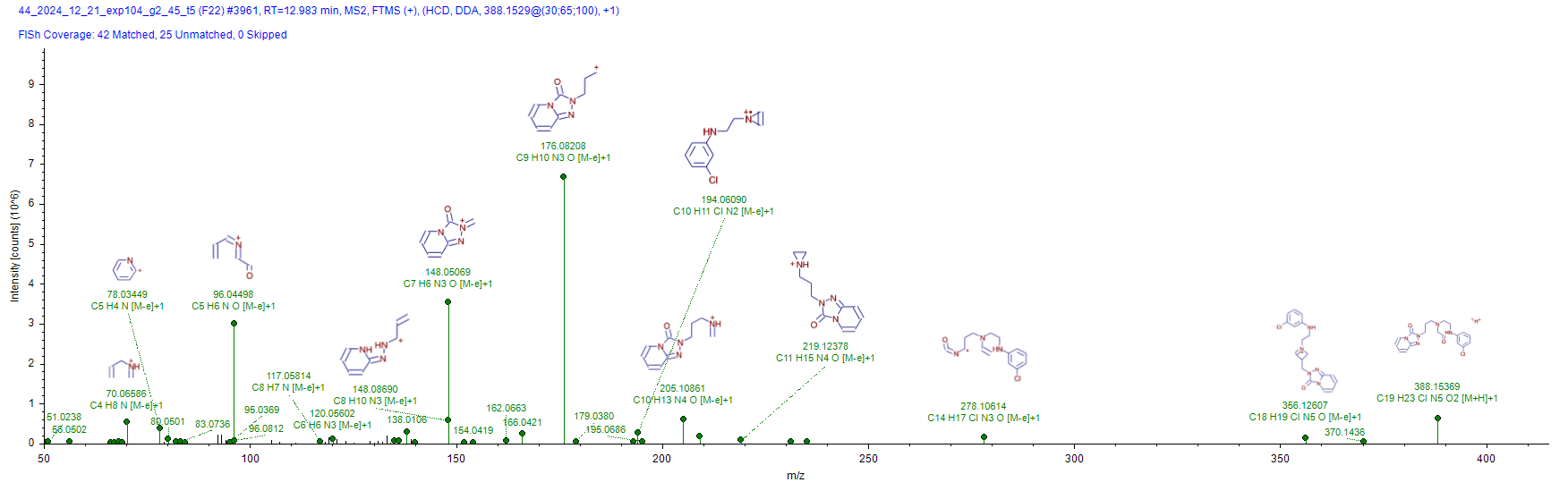


**Figure S37. FISh coverage of TRA-388 MS^2^ fragmentation.**


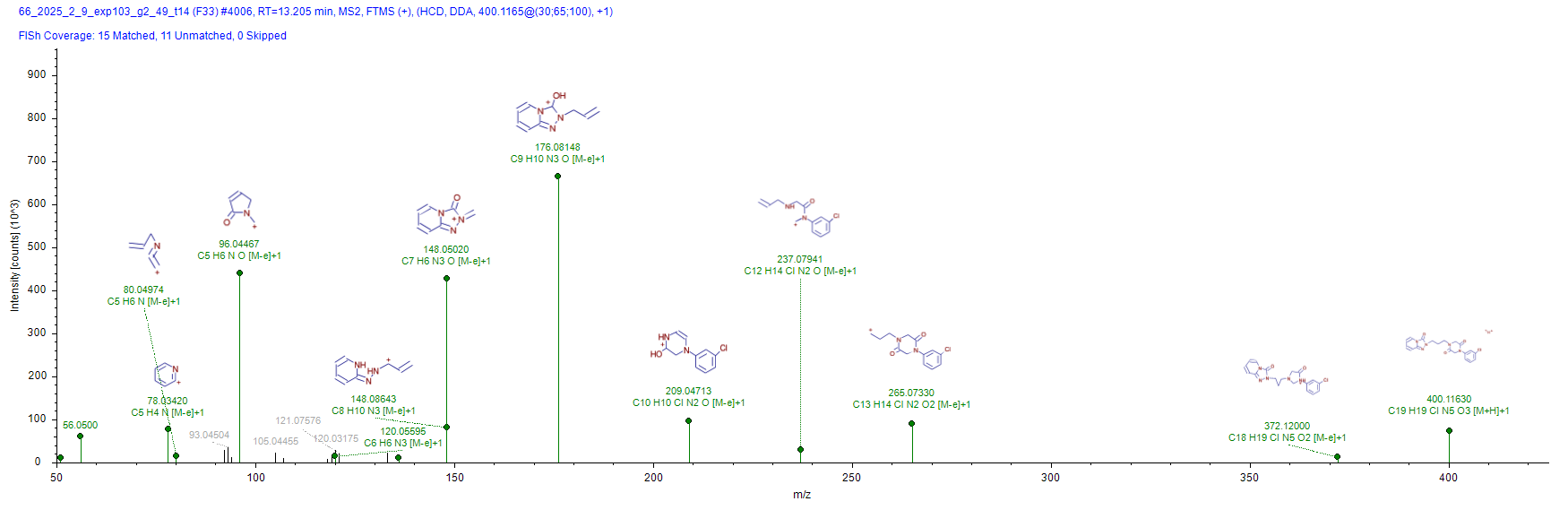


**Figure S48. FISh coverage of TRA-400 MS^2^ fragmentation.**


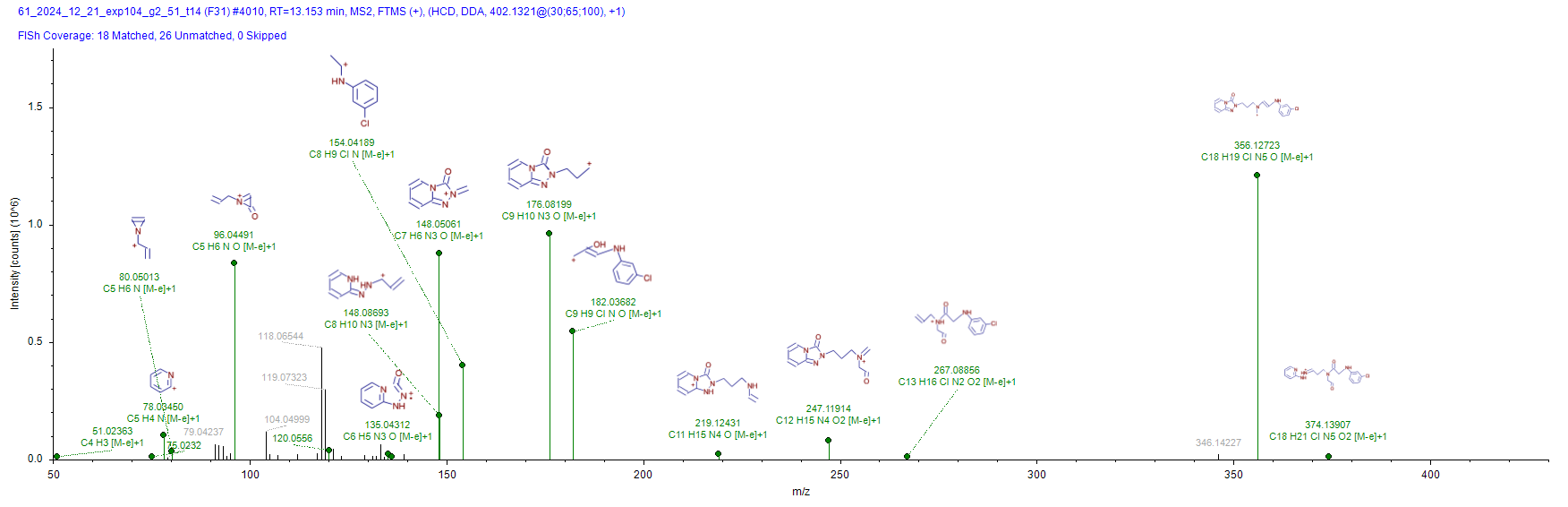


**Figure S39. FISh coverage of TRA-402 MS^2^ fragmentation.**


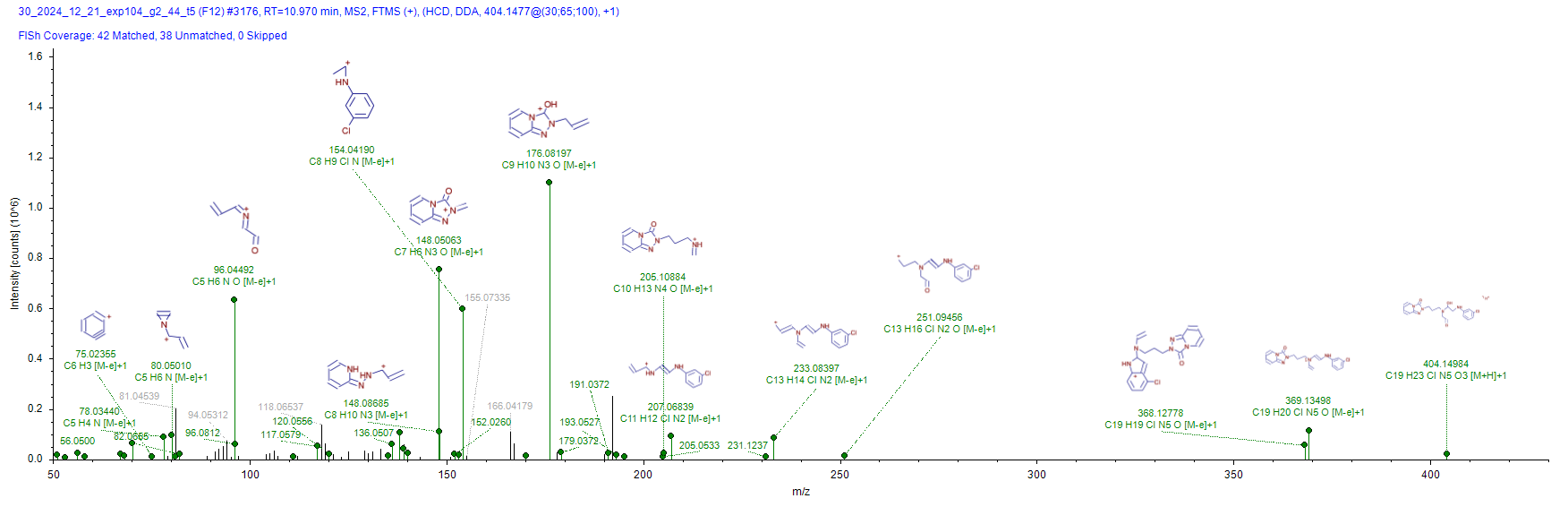


**Figure S40. FISh coverage of TRA-404 MS^2^ fragmentation.**


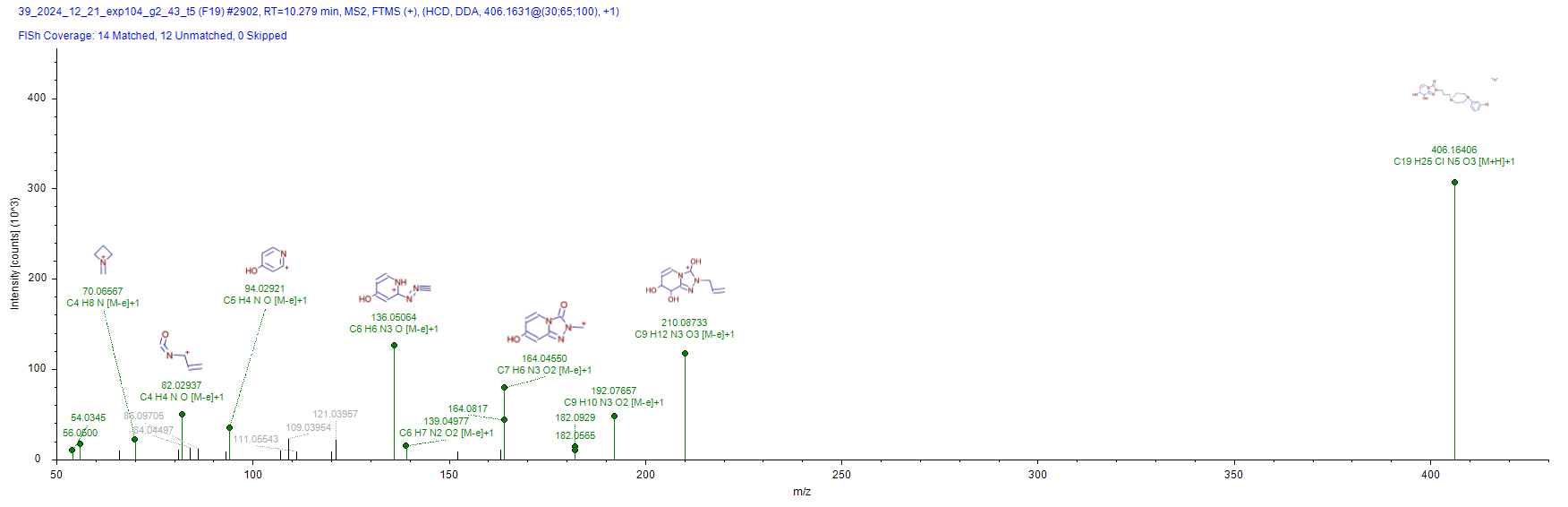


**Figure S41. FISh coverage of TRA-406 MS^2^ fragmentation.**


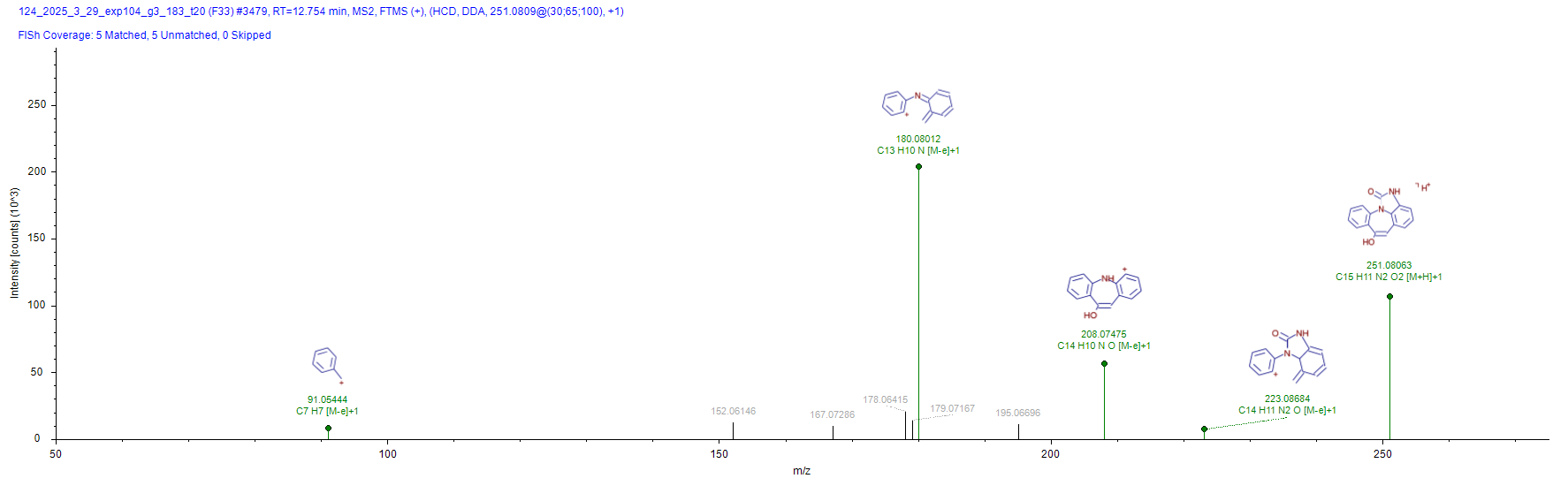


**Figure S42. FISh coverage of CBZ-250 MS^2^ fragmentation.**


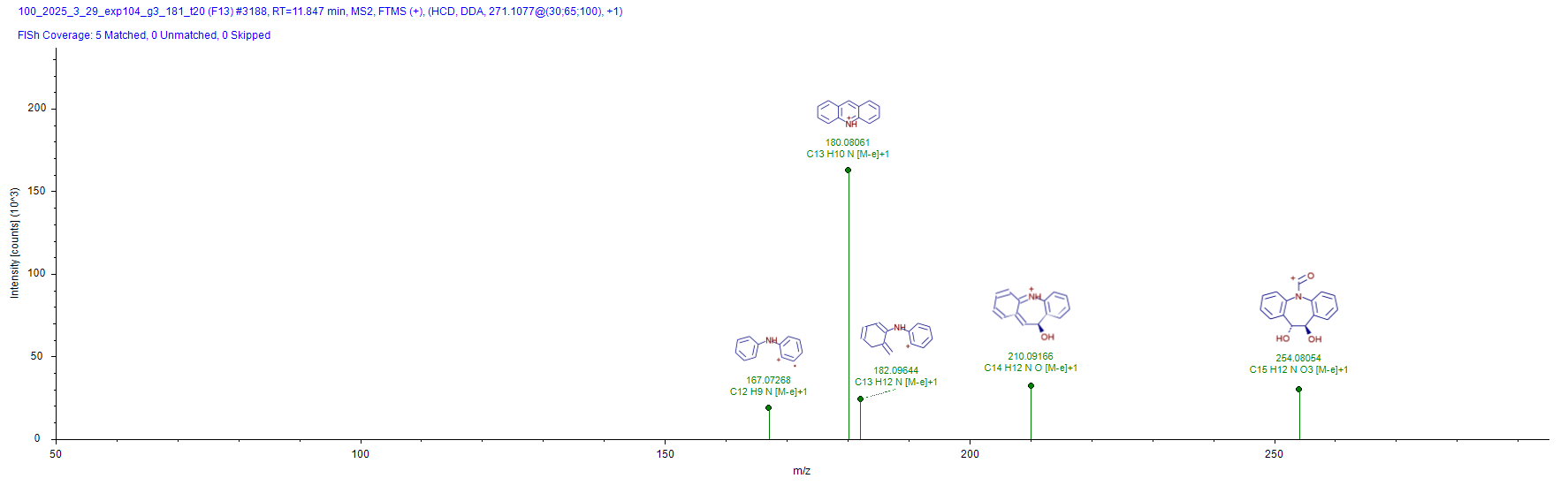


**Figure S43. FISh coverage of CBZ-271 MS^2^ fragmentation.**


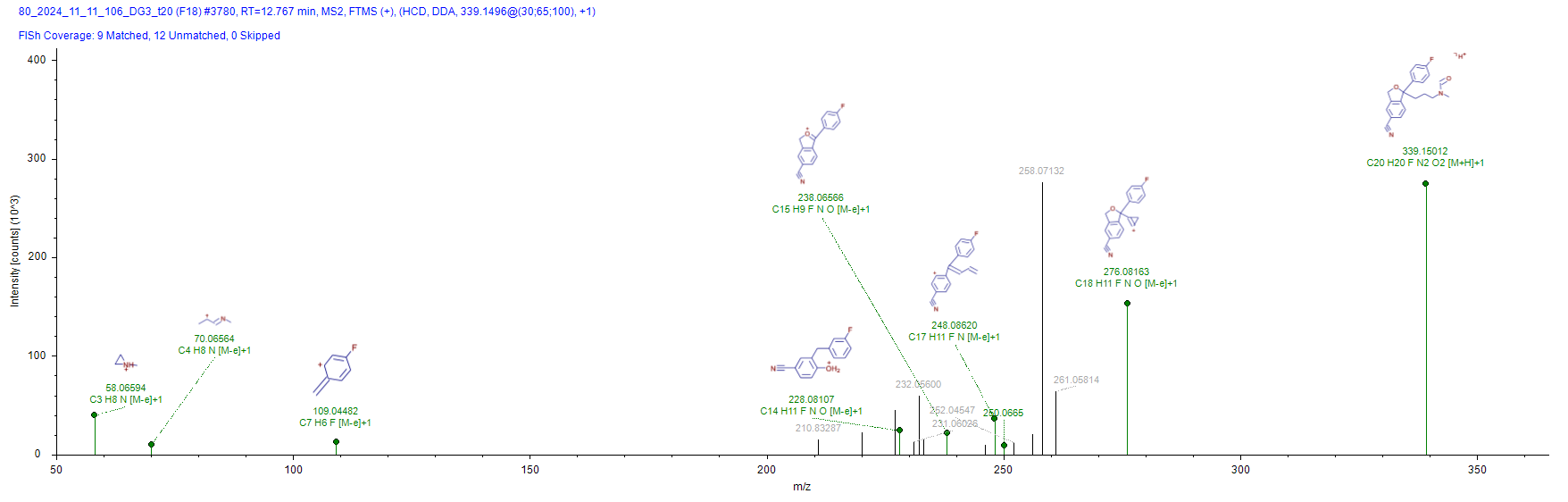


**Figure S44. FISh coverage of CTL-338 MS^2^ fragmentation.** M/z fragment ion 258.071 most likely is C_18_H_9_NF which is a result of the amine removal and the opening and removal of oxygen on the cyclopentane.


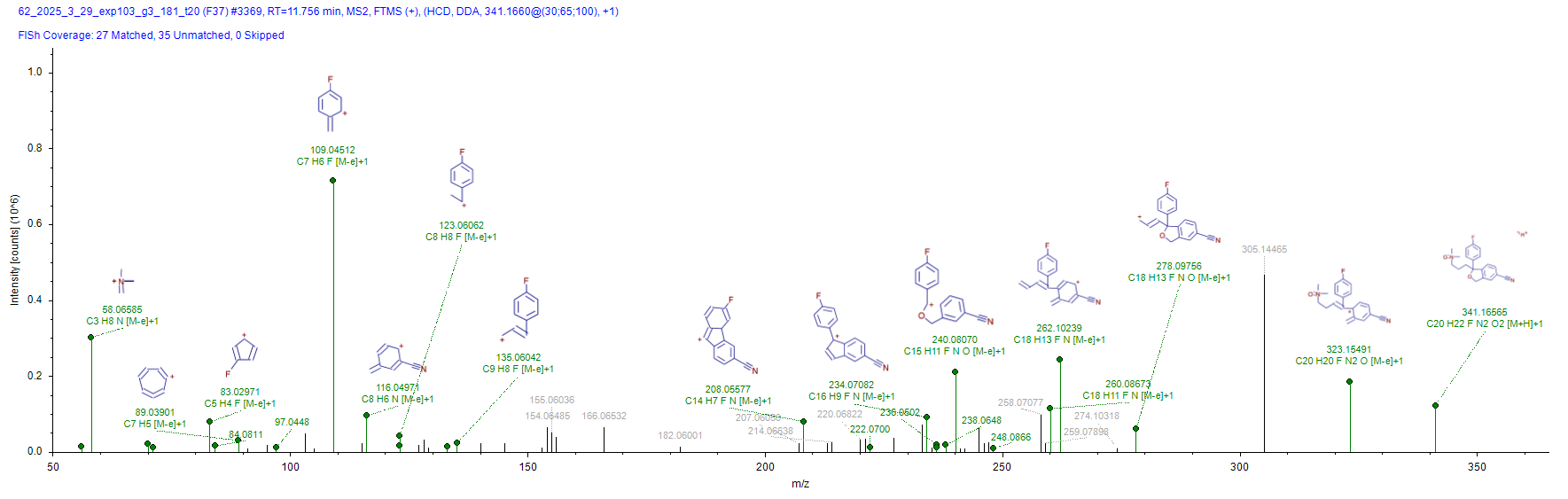


**Figure S45. FISh coverage of CTL-338 MS^2^ fragmentation.** M/z fragment ion 305.144 is likely C_20_H_18_N_2_F which is a result opening and removal of oxygen on the cyclopentane.


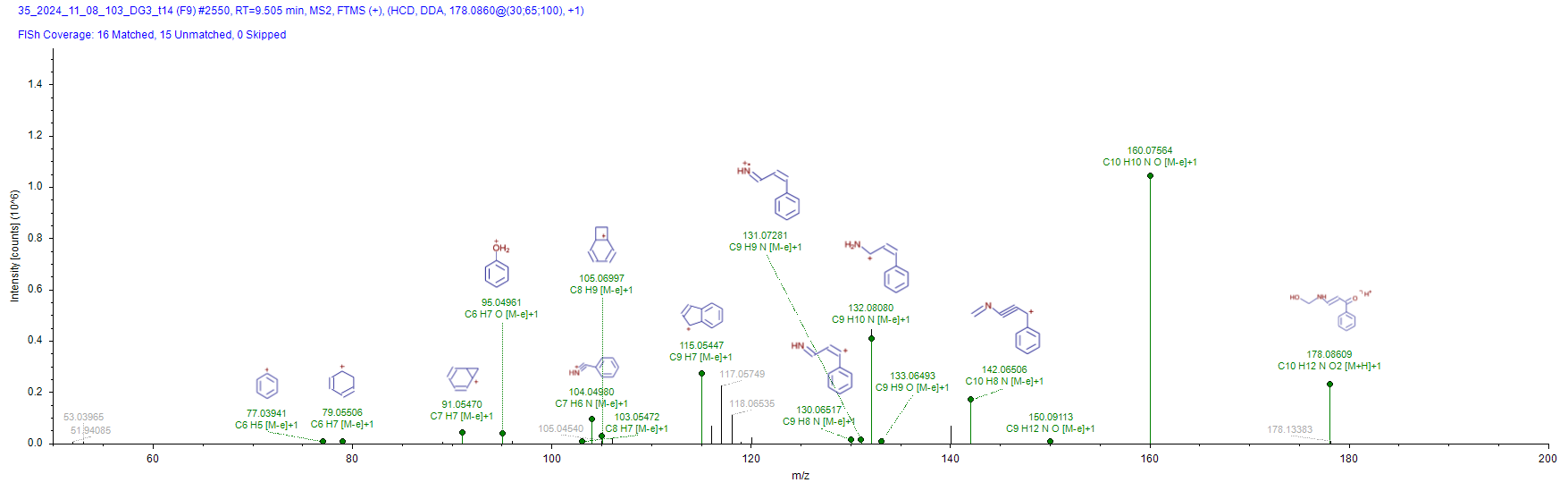


**Figure S46. FISh coverage of FLX-178 MS^2^ fragmentation.**

**References**

(1) Mercl, F.; Košnář, Z.; Maršík, P.; Vojtíšek, M.; Dušek, J.; Száková, J.; Tlustoš, P. Pyrolysis of Biosolids as an Effective Tool to Reduce the Uptake of Pharmaceuticals by Plants. *Journal of Hazardous Materials* **2021**, *405*, 124278. https://doi.org/10.1016/j.jhazmat.2020.124278.

(2) Peysson, W.; Vulliet, E. Determination of 136 Pharmaceuticals and Hormones in Sewage Sludge Using Quick, Easy, Cheap, Effective, Rugged and Safe Extraction Followed by Analysis with Liquid Chromatography–Time-of-Flight-Mass Spectrometry. *Journal of Chromatography A* **2013**, *1290*, 46–61. https://doi.org/10.1016/j.chroma.2013.03.057.

(3) Arenas, M.; Santos, J. L.; Martín, J.; Aparicio, I.; Alonso, E. Enantioselective LC-MS/MS Determination of Antidepressants, β-Blockers and Metabolites in Agricultural Soil, Compost and Digested Sewage Sludge. *Analytica Chimica Acta* **2023**, *1261*, 341224. https://doi.org/10.1016/j.aca.2023.341224.

(4) Riva, F.; Zuccato, E.; Pacciani, C.; Colombo, A.; Castiglioni, S. A Multi-Residue Analytical Method for Extraction and Analysis of Pharmaceuticals and Other Selected Emerging Contaminants in Sewage Sludge. *Anal. Methods* **2021**, *13* (4), 526–535. https://doi.org/10.1039/D0AY02027C.

(5) McClellan, K.; Halden, R. U. Pharmaceuticals and Personal Care Products in Archived U.S. Biosolids from the 2001 EPA National Sewage Sludge Survey. *Water Research* **2010**, *44* (2), 658–668. https://doi.org/10.1016/j.watres.2009.12.032.

(6) Johnson, D. J.; Sanderson, H.; Brain, R. A.; Wilson, C. J.; Bestari, K. (Jim) T.; Solomon, K. R. Exposure Assessment and Microcosm Fate of Selected Selective Serotonin Reuptake Inhibitors. *Regulatory Toxicology and Pharmacology* **2005**, *42* (3), 313–323. https://doi.org/10.1016/j.yrtph.2005.05.010.

(7) Yamamoto, H.; Hayashi, A.; Nakamura, Y.; Sekizawa, J. Fate and Partitioning of Selected Pharmaceuticals in Aquatic Environment. *Environmental Sciences* **2005**.

(8) Chefetz, B.; Marom, R.; Salton, O.; Oliferovsky, M.; Mordehay, V.; Ben-Ari, J.; Hadar, Y. Transformation of Lamotrigine by White-Rot Fungus *Pleurotus Ostreatus*. *Environmental Pollution* **2019**, *250*, 546–553. https://doi.org/10.1016/j.envpol.2019.04.057.

(9) Llorca, M.; Castellet-Rovira, F.; Farré, M.-J.; Jaén-Gil, A.; Martínez-Alonso, M.; Rodríguez-Mozaz, S.; Sarrà, M.; Barceló, D. Fungal Biodegradation of the *N*-Nitrosodimethylamine Precursors Venlafaxine and *O*-Desmethylvenlafaxine in Water. *Environmental Pollution* **2019**, *246*, 346–356. https://doi.org/10.1016/j.envpol.2018.12.008.

(10) Sośnicka, A.; Kózka, B.; Makarova, K.; Giebułtowicz, J.; Klimaszewska, M.; Turło, J. Optimization of White-Rot Fungi Mycelial Culture Components for Bioremediation of Pharmaceutical-Derived Pollutants. *Water* **2022**, *14* (9), 1374. https://doi.org/10.3390/w14091374.

(11) Kózka, B.; Sośnicka, A.; Nałęcz-Jawecki, G.; Drobniewska, A.; Turło, J.; Giebułtowicz, J. Various Species of *Basidiomycota* Fungi Reveal Different Abilities to Degrade Pharmaceuticals and Also Different Pathways of Degradation. *Chemosphere* **2023**, *338*, 139481. https://doi.org/10.1016/j.chemosphere.2023.139481.

(12) Rodarte-Morales, A. I.; Feijoo, G.; Moreira, M. T.; Lema, J. M. Degradation of Selected Pharmaceutical and Personal Care Products (PPCPs) by White-Rot Fungi. *World J Microbiol Biotechnol* **2011**, *27* (8), 1839–1846. https://doi.org/10.1007/s11274-010-0642-x.

(13) Marco-Urrea, E.; Pérez-Trujillo, M.; Vicent, T.; Caminal, G. Ability of White-Rot Fungi to Remove Selected Pharmaceuticals and Identification of Degradation Products of Ibuprofen by *Trametes Versicolor*. *Chemosphere* **2009**, *74* (6), 765–772. https://doi.org/10.1016/j.chemosphere.2008.10.040.

(14) Golan-Rozen, N.; Seiwert, B.; Riemenschneider, C.; Reemtsma, T.; Chefetz, B.; Hadar, Y. Transformation Pathways of the Recalcitrant Pharmaceutical Compound Carbamazepine by the White-Rot Fungus Pleurotus Ostreatus: Effects of Growth Conditions. *Environ. Sci. Technol.* **2015**, *49* (20), 12351–12362. https://doi.org/10.1021/acs.est.5b02222.

(15) Golan-Rozen, N.; Chefetz, B.; Ben-Ari, J.; Geva, J.; Hadar, Y. *Transformation of the Recalcitrant Pharmaceutical Compound Carbamazepine by Pleurotus ostreatus: Role of Cytochrome P450 Monooxygenase and Manganese Peroxidase*. ACS Publications. https://doi.org/10.1021/es200298t.

(16) Kózka, B.; Nałęcz-Jawecki, G.; Turło, J.; Giebułtowicz, J. Application of *Pleurotus Ostreatus* to Efficient Removal of Selected Antidepressants and Immunosuppressant. *Journal of Environmental Management* **2020**, *273*, 111131. https://doi.org/10.1016/j.jenvman.2020.111131.

(17) Vegosen, L.; Martin, T. M. An Automated Framework for Compiling and Integrating Chemical Hazard Data. *Clean Techn Environ Policy* **2020**, *22* (2), 441–458. https://doi.org/10.1007/s10098-019-01795-w.

1. [↑](#footnote-ref-1)
